# Crustal growth and reworking: A case study from the Erguna Massif, eastern Central Asian Orogenic Belt

**DOI:** 10.1038/s41598-019-54230-x

**Published:** 2019-11-27

**Authors:** Chenyang Sun, Wenliang Xu, Peter A. Cawood, Jie Tang, Shuo Zhao, Yu Li, Xiaoming Zhang

**Affiliations:** 1 College of Earth Sciences, Jilin University, Changchun, 130061 China; 2 School of Earth, Atmosphere and Environment, Monash University, Melbourne, VIC 3800 Australia; 3 Key Laboratory of Mineral Resources Evaluation in Northeast Asia, Ministry of Natural Resources of China, Changchun, 130061 China; 4 Institute of Geology, Chinese Academy of Geological Sciences, Beijing, 100037 China; 5 Institute of Geology and Geophysics, Chinese Academy of Sciences, Beijing, 100029 China

**Keywords:** Geochemistry, Tectonics

## Abstract

Despite being the largest accretionary orogen on Earth, the record of crustal growth and reworking of individual microcontinental massifs within the Central Asian Orogenic Belt (CAOB) remain poorly constrained. Here, we focus on zircon records from granitoids in the Erguna Massif to discuss its crustal evolution through time. Proterozoic–Mesozoic granitoids are widespread in the Erguna Massif, and spatiotemporal variations in their zircon ε_Hf_(t) values and T_DM2_(Hf) ages reveal the crustal heterogeneity of the massif. Crustal growth curve demonstrates that the initial crust formed in the Mesoarchean, and shows a step-like pattern with three growth periods: 2.9–2.7, 2.1–1.9, and 1.7–0.5 Ga. This suggests that microcontinental massifs in the eastern CAOB have Precambrian basement, contradicting the hypothesis of significant crustal growth during the Phanerozoic. Phases of growth are constrained by multiple tectonic settings related to supercontinent development. Calculated reworked crustal proportions and the reworking curve indicate four reworking periods at 1.86–1.78 Ga, 860–720 Ma, 500–440 Ma, and 300–120 Ma, which limited the growth rate. These periods of reworking account for the crustal heterogeneity of the Erguna Massif.

## Introduction

The Earth is perhaps unique in the solar system in having a chemically evolved felsic continental crust^1^. The growth of continental crust is a record of the volume of new crust generated from the mantle through time less the amount recycled to the mantle^2^. Estimates of the tie of the growth history of continental crust range from rapid growth early in Earth evolution^3^ to more progressive growth through time^4–9^. The Central Asian Orogenic Belt (CAOB), as the largest accretionary orogenic belt on Earth, is composed of a series of island arcs, ophiolite, oceanic islands, seamounts, accretionary wedges, oceanic plateau and microcontinents^10–12^, and provides an important natural laboratory in which to understand the crustal evolution history.

Crustal growth within the CAOB occurred mainly during the Phanerozoic, as inferred from the highly positive ε_Nd_(t) values and low initial ^87^Sr/^86^Sr (I_Sr_) values of granitoids in the belt^10,13–16^. Additional evidence suggests that this phase of crustal growth was focused in continental arc settings between microcontinental massifs, rather than within the individual microcontinents^17,18^. Kröner *et al*. however, on the basis of Nd-Hf isotope analyses on felsic magmatic rocks, argued that the production of juvenile continental crust within the CAOB has been grossly overestimated^19^.

Zircon, as an accessory mineral in igneous rocks, has been widely used to investigate the origins of magmatic rocks and document crustal evolution from the pluton- to global-scale^9^. In this study, we analyzed *in situ* zircon U-Pb and Lu-Hf isotope ratios of 66 Proterozoic–Mesozoic granitoids from the Erguna Massif by laser ablation–inductively coupled plasma–mass spectrometry (LA-ICP-MS), with the aim of constraining the record of crustal growth and reworking processes of microcontinental massifs within the orogenic belt, and exploring underlying geodynamic mechanisms. Based on these results, we established the crustal growth curve of the Erguna Massif and reveal the crustal evolution history of the study region.

## Geological Background

**Figure 1:**
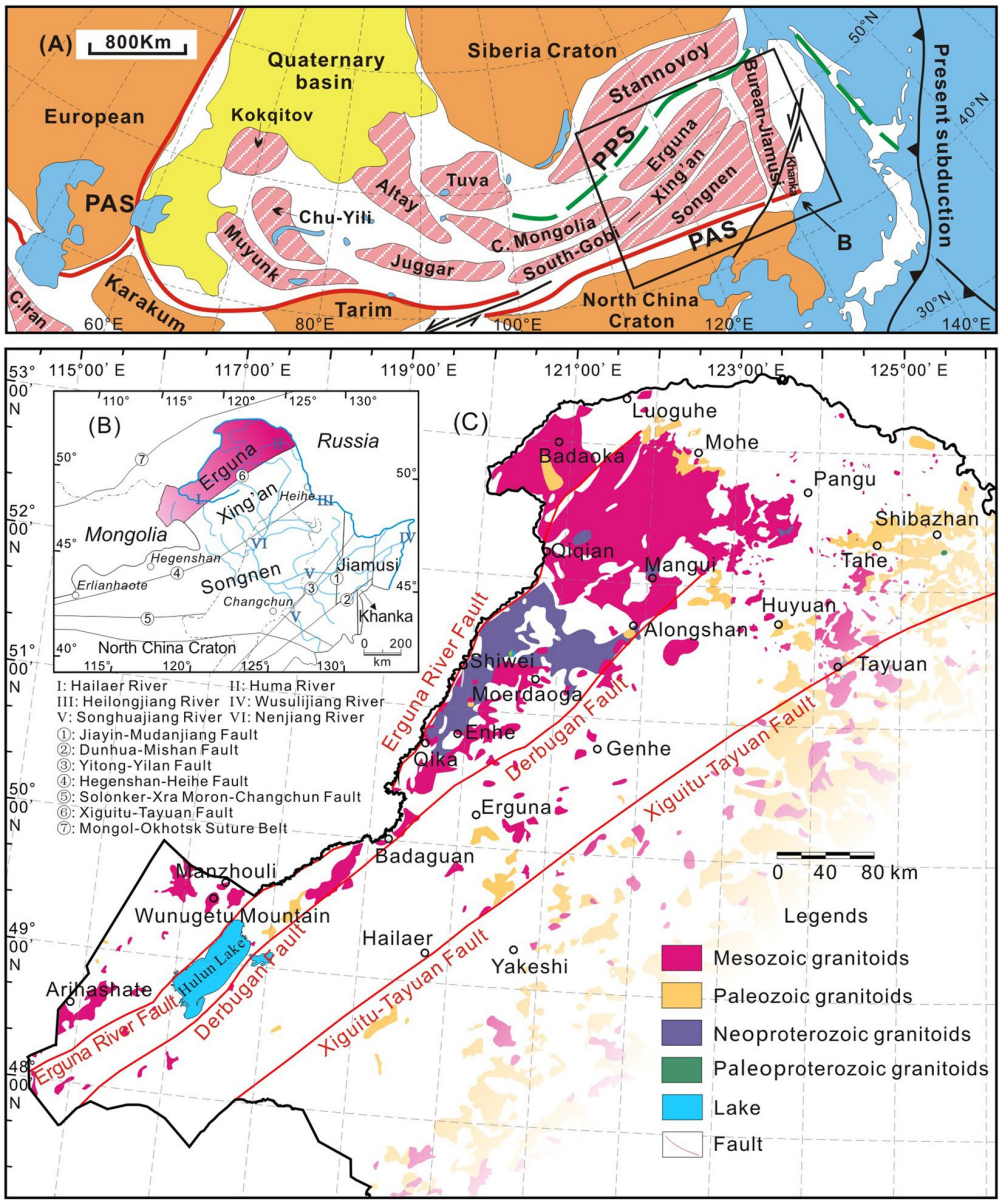
(**A**) Simplified tectonic map of the Central Asian Orogenic Belt (after Li^21^, PPS: Paleo-Pacific suture; PAS: Paleo-Asian suture). (**B**) Regional tectonic framework of NE China, showing major tectonic divisions and location of the study area (after Wu *et al*.^20^). (**C**) Distribution of granitoids in the Erguna Massif (sample locations are summarized in Supplementary Table S1).

The CAOB extends from the Ural Mountains to the Pacific, and from the Siberian and East European (Baltica) cratons to the North China and Tarim cratons (Fig. 1A). The eastern CAOB runs through NE China, where it comprises a series of microcontinental massifs (including, from west to east, the Erguna, Xing’an, Songnen, Jiamusi and Khanka massifs) separated by suture belts, and with voluminous granitoids occurring in both^10,20^ (Fig. 1B). The Paleozoic tectonic evolution of NE China was dominated by the closure of the Paleo-Asian Ocean and the amalgamation of the microcontinental massifs, whereas its Mesozoic tectonic evolution was characterized by the overprinting of the circum-Pacific and Mongol-Okhotsk tectonic regimes^20–23^.

The Erguna Massif is bounded by the Xiguitu-Tayuan Fault to the southeast and the Mongol-Okhotsk suture belt to the northwest (Fig. 1B). Granitoids are exposed across some 40,000 km^2^ of the massif, most of which lie to the north of the Derbugan Fault, and north of Erguna (Fig. 1C). Proterozoic granitoids are exposed mainly in the north–central part of the massif, including the Qiqian, Mangui, Shiwei and Shibazhan areas, whereas Paleozoic granitoids are exposed mainly to the northeast of the massif. Mesozoic granitoids are widespread in the Erguna Massif and are inferred to have formed during southward convergence of the Mongol-Okhotsk Ocean^17^.

## Sample and Method

Granitoids dominate the magmatic record preserved in the CAOB and are similar in composition to the bulk continental crust^24^. In this paper, zircon data from granitoids are used as a proxy for felsic crustal growth and reworking. To eliminate bias arising from cluster sampling, sample selection was based on the following criteria: (1) granitoids of the same age should be spatially separated; (2) granitoids with different ages should be selected from the same region; (3) the different age proportions relative to the entire data should be equal to the exposed proportion of granitoids with corresponding age relative to the total area.

**Figure 2:**
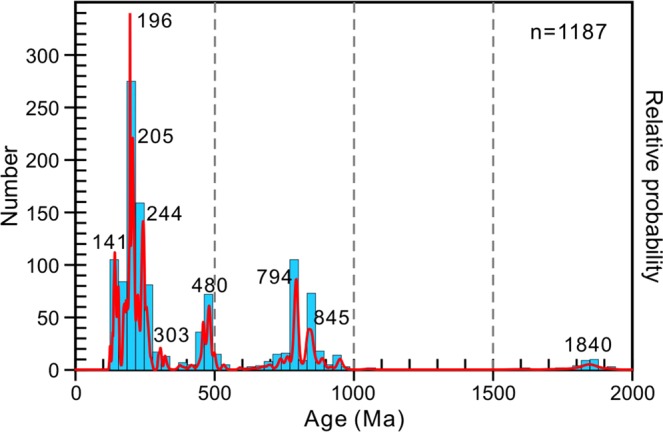
Histogram and relative probability plot of U-Pb ages of granitoids in the Erguna Massif. Age data and references are listed in Supplementary Tables S1 and S2, respectively.

1187 magmatic zircon U-Pb dating results obtained from 66 representative granitoids within the Erguna Massif indicate that these granitoids were emplaced during the Paleoproterozoic, Neoproterozoic, Paleozoic, and Mesozoic, with ages ranging from 1860 to 125 Ma (Fig. 2). The locations, ages and reference citations for the 66 samples are listed in Supplementary Table S1, and zircon U-Pb dating results are summarized in Supplementary Table S2. Geochemically, these granitoids are mostly subalkaline, and include A- and I-type granitoids as well as minor adakitic rocks. In addition, they have high concentrations of SiO_2_ (>65%) and Al_2_O_3_, and low concentrations of Mg^#^, ^T^Fe_2_O_3_, Cr, Co and Ni, thereby excluding the possibility of mixing between the granitic melts and mantle-derived mafic magma. The geochemical features of these granitoids (e.g., enrichment in light rare-earth elements and large ion lithophile elements, and depletion in heavy rare-earth elements and high field-strength elements) indicate that their primary magmas were derived from partial melting of the lower continental crust.

The zircon Hf isotopic data obtained from 582 dated zircons are summarized in Supplementary Table S3. Initial ^176^Hf/^177^Hf values are expressed as ε_Hf_(t), which is calculated using a decay constant value of 1.867 × 10^−11^ yr^−1 25^, giving ^176^Hf/^177^Hf_(CHUR, today)_ and ^176^Lu/^177^Hf_(CHUR, today)_ of 0.282772 and 0.0332, respectively^26^. For the calculation of Hf isotopic model ages, the global depleted mantle value is used as the juvenile end-member, with ^176^Hf/^177^Hf = 0.28325 and ^176^Lu/^177^Hf = 0.0384^27^, and ^176^Lu/^177^Hf_(Crust, today)_ value of 0.015 is used for the average continental crust^28^.

## Discussion

### Heterogeneity of the continental crust in the Erguna Massif

**Figure 3:**
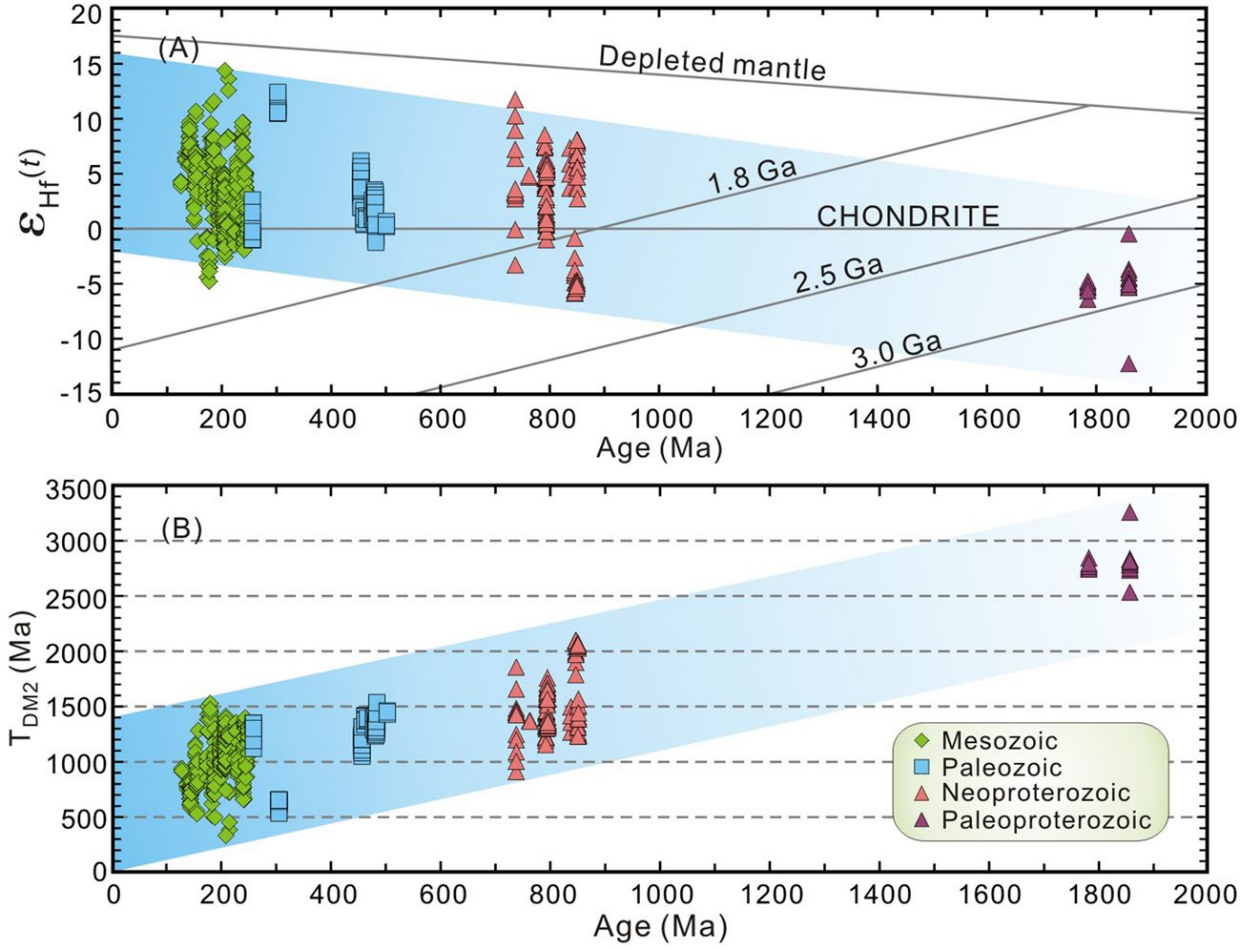
Plots of temporal variations in zircon Hf isotopic compositions (data from Supplementary Table S3, n = 582). (**A**) Zircon ε_Hf_(t) values versus crystallization ages. (**B**) Zircon T_DM2_ (Hf) ages versus crystallization ages.

The ε_Hf_(t) values and Hf two-stage model (T_DM2_) ages of zircons from the Proterozoic, Paleozoic and Mesozoic granitoids in the Erguna Massif are summarized in Fig. 3. Zircon ε_Hf_(t) values gradually increase through time (Fig. 3A), whereas T_DM2_ (Hf) ages gradually decrease (Fig. 3B). This implies a change in the granitoid magma source, from the melting of ancient crust to the melting of juvenile crust.

**Figure 4:**
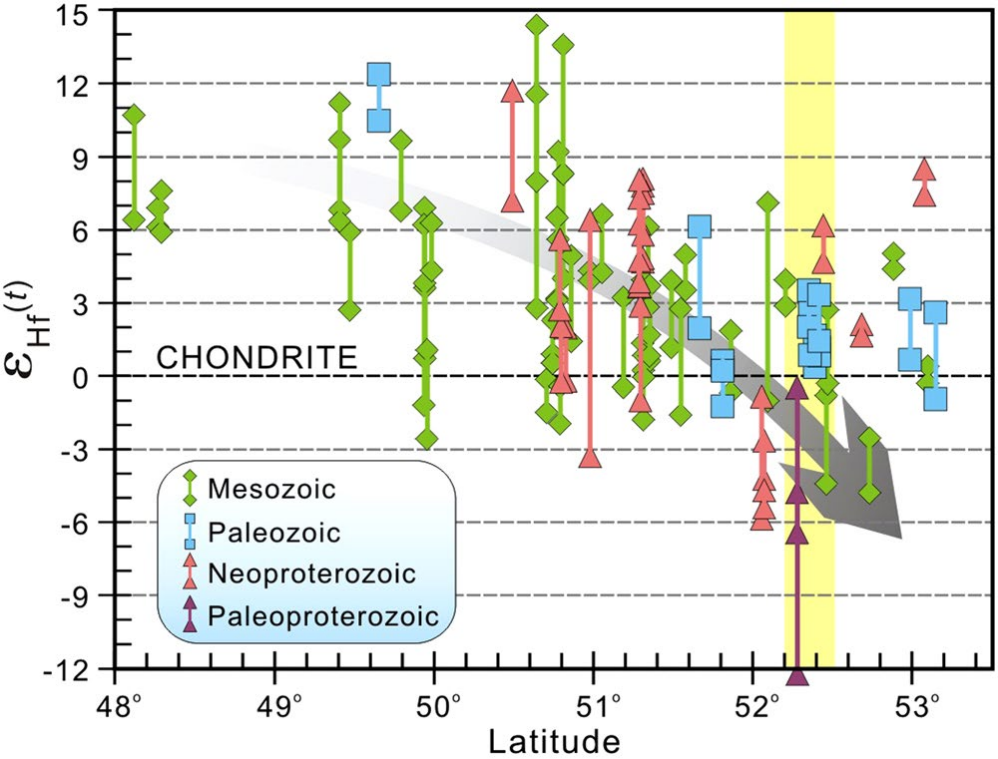
Plot of zircon ε_Hf_(t) values versus latitude of sample locations, showing lateral and vertical heterogeneities in the continental crust of the Erguna Massif.

Spatial variations are also observed from the zircon Hf isotopes, i.e., ε_Hf_(t) values gradually decrease northward (Fig. 4), indicating an increasing component of ancient crustal material from south to north. The inference is supported by the occurrence of the Neoproterozoic and Paleoproterozoic granitoids mainly in the central–north part of the Erguna Massif. Furthermore, there is a wide range in zircon Hf isotopic compositions at given latitude (yellow shading in Fig. 4). Taken together, the spatiotemporal variations in zircon Hf isotopic compositions indicate a heterogeneous lower continental crust beneath the Erguna Massif.

### Crustal growth within the Erguna Massif

The model age of zircon Hf-isotope of granitoids reflect the timing of older mantle-derived material emplaced into the crust (i.e. crustal generation), which were remelted to produce the host magmas of the younger zircons, whereas U-Pb ages reflect the timing of later crustal magmatic events (i.e. crustal reworking). Since the volume and rate of crustal recycling are difficult to calculate separately or simulate over time from crustal generation and reworking, we consider that the net increase in crustal volume (crustal growth) during magmatic episodes depends on the proportions of newly formed and reworked crust that are preserved over time^6,8^. The approach of Belousova *et al*.^6^ has been applied to estimate the crustal volume of the Erguna Massif through time based on zircon U-Pb ages and their Hf-isotope ratios.

**Figure 5:**
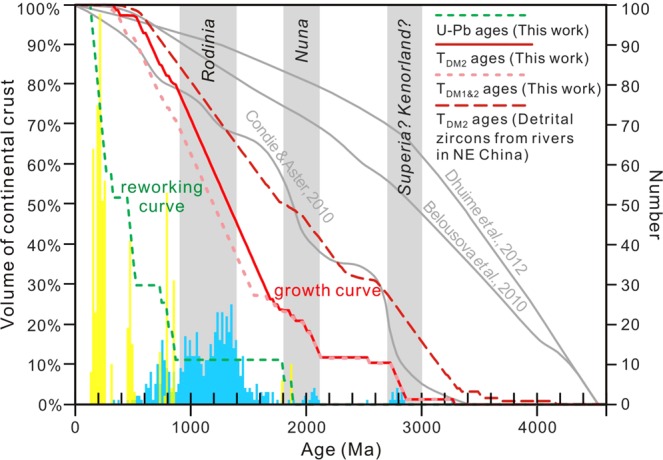
Distribution of zircon T_DM2_ (Hf) ages (blue bars) and U-Pb ages (yellow bars) from the Erguna Massif, and crustal evolution curves using different datasets. The histograms were calculated using bins of 20 Myr. Two crustal growth curves and the reworking curve of the Erguna Massif are shown in solid red, dashed pink, and dashed green, respectively. For comparison, also shown are the crustal growth curve (dashed red curve) established from detrital zircons in modern rivers of NE China (using the method of this study and data from Li^31^) and three crustal growth curves^6–8^ using global datasets and calculated by different methods (grey curves).

According to the classification scheme for granitoid lineage^29^, it is stated that a “superunit” is the largest rock unit to form during a single magmatic event, the duration of which does not exceed 20 Myr. Thus, we calculated the proportions of the total age data that fell within 20-Myr time intervals. For each interval, *N*_*U-Pb age*_ is the number of zircons that yield crystallization ages coinciding with the interval, and *N*_*model age*_ is the number of zircons with T_DM2_ (Hf) ages coinciding with the interval (Fig. 5). Because both reworked and juvenile crust could have existed in a given period, we apply the obtained proportions of zircon T_DM2_ (Hf) ages from granitoids in the Erguna Massif to calculate the distributions of juvenile crust through time. According to Belousova *et al*.^6^, the juvenile proportion (*X*_*juv*_) is estimated as:

1 $${X}_{juv}=100{\rm{ \% }}\times {N}_{modelage}/({N}_{U-Pbage}+{N}_{modelage}).$$

The obtained values of *X*_*juv*_ yield a crustal growth curve for the Erguna Massif (solid red curve in Fig. 5) that shows the increase in crustal volume over time. This curve to some degree circumvents the calculation bias of the geological record in a single type toward zircon crystallization ages or model ages. These granitoids record three stages of crustal development in the Erguna Massif: Archean crust formation at 2.9–2.7 Ga, a second growth event at 2.1–1.9 Ga, and a major and protracted growth process at 1.7–0.5 Ga, resulting in a step-like crustal growth curve (Fig. 5).

Note that, most of the crustal models, including that in this paper, are based on zircon records that focus on felsic crustal evolution and have not considered the mafic crustal compositions^2,30^. Furthermore, T_DM2_ (Hf) ages from granitoids are often used to represent the formation time of juvenile mafic crust, although some younger results might be produced by mixing between melts with more radiogenic Hf and older crustal material, especially the data with ε_Hf_(t) values between 0 and depleted mantle values. In order to better understand the potential influence of these factors, we not only summarised the data from mafic igneous rocks in the Erguna Massif (Supplementary Table S4), but also made some changes when we used the model ages. Firstly, the Hf/Nd isotopic single-stage model ages from mafic igneous rocks range from Mesoproterozoic to Neoproterozoic, corresponding to the rapid increase of the crustal volume from 1.7 Ga (~26%) to 0.5 Ga (~97%) in the growth curve of the Erguna Massif (Fig. 5). This highlights that the Meso-Neoproterozoic was the major period of crustal generation and growth of the Erguna Massif, rather than the Phanerozoic as suggested in previous studies^14,15^. Secondly, we reprocessed zircon Hf isotopic data, such that single-stage “mantle” model Hf ages (T_DM1_) are calculated when their ε_Hf_(t) values are positive, whereas two-stage model ages are used when their ε_Hf_(t) values are negative. The resultant growth curve is shown as the dashed pink line in Fig. 5, which can be considered as minimum estimate of crustal growth. There is no obvious change in this growth curve from solid red line, except a delay in crustal growth around 1.7~1.6 Ga (Fig. 5). Thus, we do not consider that mixing process significantly biased the average crustal growth pattern^6^.

To understand the bias between igneous and sedimentary zircon records during the process of crustal growth in the study region, another additional crustal growth curve has been calculated (dashed red curve in Fig. 5), based on Hf isotope data of detrital zircon grains from major river systems (the Hailaer, Huma, Heilongjiang, Wusulijiang, Songhuajiang, and Nenjiang rivers) in NE China^31^ (Fig. 1B). This also shows a step-like pattern, reflecting three crustal growth periods at 3.3–2.6, 2.4–2.0, and 1.8–0.5 Ga (Fig. 5). Relative to the growth curve of the Erguna Massif, detrital zircons from these rivers contain more information about T_DM2_ (Hf) ages of 2.2–2.4 and >3.0 Ga. However, rock units of these ages are widely distributed in the North China Craton, and the Songhuajiang River incorporates data from the craton^31^ (Fig. 1B).

Compared with growth curves compiled from worldwide data, the starting time in the Erguna Massif curve is “delayed” to 3.2–2.9 Ga. This reflects the small area of Precambrian rocks exposed in the study area. If ancient crust had been present, it must have been eroded or recycled to the mantle. Although igneous provinces are by definition restricted in space and time, and the growth curve established from granitoids in the Erguna Massif may provide an under-estimate of the crustal growth rate, the evidence from detrital zircon data from modern rivers in NE China yields a similar delayed start and step-like growth pattern of crustal evolution. Thus, we propose that the contribution of ancient crust to the growth of the overall continental crust in this region is not significant.

### Crustal reworking within the Erguna Massif

Unlike the model ages of zircon Hf isotopes, the proportion of reworked crust through time is given by the distribution of zircon crystallization ages. Histograms of zircon U-Pb ages and T_DM2_ (Hf) ages of granitoids in the Erguna Massif are shown in Fig. 5 (yellow and blue bars, respectively). The two group of ages overlap in the range of 860–720 Ma, indicating that both crustal growth and reworking occurred during this period.

The reworked proportion (*X*_*rew*_) can therefore be calculated as:

2 $${X}_{rew}=100 \% -{X}_{juv}$$

The proportion of reworked crust of the Erguna Massif also shows a step-like curve (dashed green curve in Fig. 5), reflecting four periods of crustal reworking at 1.86–1.78 Ga, 860–720 Ma, 500–440 Ma, and 300–120 Ma. The crustal evolution of the Erguna Massif was dominated by crustal reworking after 500 Ma (when the reworked proportion increased from ~34% to 100%), but not growth, which is consistent with the emplacement ages of the granitoids in the eastern CAOB.

Considering the relationship between zircon U-Pb ages and T_DM2_ (Hf) ages (Fig. 3), and the variations in ε_Hf_(t) values with latitude (Fig. 4), we suggest that reworking was an important factor leading to the heterogeneity of the continental crust, as it was controlled by several regional tectonic processes that occurred on different geological timescales. Additionally, with gradual reworking of pre-existing crust within the Erguna Massif, the model age of source materials of magmatic episodes evolved to more juvenile through time.

### Crustal evolution within the Erguna Massif and tectonic controls

The continental crust of the Erguna Massif represents only a small portion of the Earth’s crust. Therefore, compared with more global data^6,8^ (Fig. 5), the crustal curves of the Erguna Massif highlight the role of tectonics in shaping regional crustal evolution. The region records three periods of crustal development, at 2.9–2.7, 2.1–1.9, and 1.7–0.5 Ga (Fig. 5). These stages are similar to those proposed by Condie and Aster^7^, estimated from the global areal distributions of juvenile rocks with known ages (Fig. 5).

The first growth period at 2.9–2.7 Ga with the early phase of supercraton assembly (possibly Superia, Kenorland, or Vaalbara), the existence of which have been inferred from global granitoid age peaks and Nd isotope distributions^7,32,33^. The second growth period at 2.1–1.9 Ga corresponds with the formation of the Nuna supercontinent, which is characterized by widespread plutonism between 2.1 and 1.8 Ga^34^. The pause in crustal growth at 1.9–1.8 Ga is likely caused by crustal reworking, which related to collisional assembly of the core components of the Nuna supercontinent^34,35^. Detrital zircon data from the rivers in NE China, also suggests that the rate of crustal growth slows during 1.9–1.7 Ga, indicating the recycling rate of continental crust approaches the production rate as reflected in the gentle slope of the curve (Fig. 5). The curve inflection at ~1.7 Ga is considered a regional signal in the crustal evolution of the Erguna Massif, indicating a decrease in the rate of crustal destruction, coincident with the onset of the longest period of rapid growth (1.7–0.5 Ga) during the Mesoproterozoic to Neoproterozoic. In addition, crustal reworking represented by a bimodal igneous rock association of gabbro-diorites and granitoids of A-type affinities formed in an extensional environment related to the breakup of the Rodinia supercontinent (860–720 Ma)^36^, and resulted in a slight fluctuation in the crustal growth curve (Fig. 5). The overall calc-alkaline andesitic composition of continental crust suggests that most of the crust was generated by processes similar to those operating at modern-day convergent plate margins^2^, implying that magmatic arcs are the major source of continental growth^2,30,37,38^. The lateral crustal growth of the Erguna Massif along subduction zones mainly happened during the assembly of supercontinents.

Crustal growth in the Erguna Massif largely ceased after the completion of the Rodinia supercontinent cycle (i.e., after the mid-Neoproterozoic). From this time, the crustal evolution of the Erguna Massif was dominated by the reworking of pre-existing crust (Fig. 5). Studies of the Early Paleozoic igneous rocks indicate that the closure process of the branch of Paleo-Asian Ocean between the Erguna and Xing’an massifs happened during 500–440 Ma along the Xiguitu-Tayuan suture zone^22,23^. Numerous Mesozoic igneous rocks in the Erguna Massif were formed during southward subduction, collision, and post-collisional extension of the Mongol-Okhotsk oceanic plate^17,23^. Affected by these subducting slabs, the lower continental crust of the Erguna Massif was heated and partially melted, with magma emplaced into the upper crust resulting in the widespread granitoids in the Erguna Massif. The Precambrian tectono-magmatic records are strongly concealed by the arc and intraplate magmatic activities that occurred during the evolution of these two tectonic regimes, indicating that the proportion of reworked crust increased rapidly at 500–440 and 300–120 Ma (Fig. 5), and there was a change in the source of the granitic magmas from partial melting of ancient crust to juvenile crust (Fig. 3).

## Summary

Cluster sampling of granitoids, based on consideration of their spatiotemporal distribution, can be used to reveal the crustal evolution within individual regions. The evidence from zircon U-Pb ages and Hf isotopic compositions of granitoids from the Erguna Massif indicates episodic crustal growth. The growth curve for the massif commences in the Mesoarchean (3.2–2.9 Ga) and shows a step-like pattern with three growth periods at 2.9–2.7, 2.1–1.9, and 1.7–0.5 Ga. The pulses of crustal growth linked to the development of supercontinents. Although plate tectonics is a continuous process on a global scale, it can result in Erguna Massif episodic crustal evolution as recorded in the specific region. Approximately 65% of the continental crust in the massif appears to have formed in the Meso-Neoproterozoic. We show that no significant crustal growth occurred during the Phanerozoic, in contrast to previous findings, implying that microcontinental massifs (in this case the Erguna Massif) in the eastern CAOB have Precambrian basement. Four age peaks of granitoid magmatism in the Erguna Massif are identified at 1.86–1.78 Ga, 860–720 Ma, 500–440 Ma, and 300–120 Ma. These processes were linked to the reworking of pre-existing crust, which slowed the rate of crustal growth and also caused the model age of source materials of magmatic episodes to become more juvenile through time. The occurrence of spatiotemporally variable crustal reworking might have led to the heterogeneous continental crust beneath the Erguna Massif.

## Supplementary information

Supplementary Information

## Data Availability

The dataset we used in the study can be found in Supplementary Information of the manuscript.
