# Supplementary material for: Crustal growth and reworking: A case study from the Erguna Massif, eastern Central Asian Orogenic Belt
Source: Sci Rep. 2019 Nov 27;9:17671. doi: 10.1038/s41598-019-54230-x (PMC6881325; doi:10.1038/s41598-019-54230-x)
Supplement: Supplementary file 1 — Supplementary Information [file 41598_2019_54230_MOESM1_ESM.doc]

**Crustal growth and reworking: A case study from the Erguna Massif,** **eastern Central Asian Orogenic Belt**

**Chenyang Sun^1,2^, Wenliang Xu^1^****^,3^,**^*^**, Peter A. Cawood^2^, Jie Tang^1,3^, Shuo Zhao^4^, Yu Li^5^, and Xiaoming Zhang^1^**

^1^College of Earth Sciences, Jilin University, Changchun 130061, China

^2^School of Earth, Atmosphere and Environment, Monash University, Melbourne, VIC 3800, Australia

^3^Key Laboratory of Mineral Resources Evaluation in Northeast Asia, Ministry of Natural Resources of China, Changchun 130061, China

^4^Institute of Geology, Chinese Academy of Geological Sciences, Beijing 100037, China

^5^Institute of Geology and Geophysics, Chinese Academy of Sciences, Beijing 100029, China

^*^Corresponding author: Wenliang Xu (email: [xuwl@jlu.edu.cn](mailto:xuwl@jlu.edu.cn))

**Supplementary Table S1**. The locations, ages and reference citations for the Proterozoic–Phanerozoic granitoids in the Erguna Massif

| **Order** | **Sample** | **Latitude (N)** | **Longitude (E)** | **Location** | **Lithology** | **Age (Ma)** | **1σ** | **Age-**  **References** | **Geochemistry-**  **References** | **Hf-**  **References** |
| --- | --- | --- | --- | --- | --- | --- | --- | --- | --- | --- |
| 1 | 11ER16-6 | 50°58’38” | 119°41’05” | Jiuka | Monzogranite | **125** | **2** | Ref.^39^ | — | This paper |
| 2 | MZ14-2 | 48°15’56” | 117°05’05” | Baogedewula mountain | Syenogranite | **134** | **2** | Ref.^40^ | — | Ref.^17^ |
| 3 | 12ER8-1 | 50°46’47” | 119°31’45” | Qika | Syenogranite | **140** | **1** | Ref.^39^ | | |
| 4 | 17ER9-1 | 49°24’22” | 117°35’55” | Western Zhalainuoer | Granite porphyry | **141** | **1** | This paper | Unpublished | This paper |
| 5 | DB-01 | 48°17’10” | 117°02’41” | Baogedewula | Granite porphyry | **143** | **1** | Ref.^40^ | Unpublished | Ref.^17^ |
| 6 | 13ER48-1 | 49°56’37” | 118°50’16” | Badaguan | Syenogranite | **150** | **2** | Ref.^39^ | | |
| 7 | 12ER2-1 | 49°56’30” | 118°49’55” | Southwestern Badaguan | Syenogranite | **152** | **2** | Ref.^39^ | | |
| 8 | DB-03 | 48°07’00” | 116°45’00” | Baogedewula | Granite porphyry | **153** | **1** | Ref.^40^ | — | Ref.^17^ |
| 9 | 12ER1-2 | 49°56’13” | 118°49’50” | Badaguan | Syenogranite | **155** | **3** | Ref.^39^ | Unpublished | This paper |
| 10 | MZ23-1 | 49°28’16” | 117°00’43” | Chalataolegai mountain | Monzogranite | **171** | **2** | Ref.^40^ | — | Ref.^17^ |
| 11 | 12ER31-1 | 52°27’49” | 122°32’16” | Northern Mangui | Syenogranite | **173** | **2** | Ref.^17^ | Unpublished | Ref.^17^ |
| 12 | 14ER14-1 | 52°44’02” | 123°11’06” | Southern Amuer | Monzogranite | **177** | **1** | Ref.^17^ | Unpublished | This paper |
| 13 | MZ18-2 (12MZ2) | 49°24’27” | 117°19’13” | Wunugetu mountain | Monzogranite | **180** | **2** | Ref.^40^ | Ref.^41^ | |
| 14 | ZKS1-1 | 50°48’37” | 120°04’52” | Enhe | Monzogranite | **185** | **1** | Ref.^40^ | — | Ref.^41^ |
| 15 | 13ER14-1 (12ER32) | 52°05’41” | 121°53’36” | Mangui–Qiqian highway | Monzogranite | **185** | **2** | Ref.^17^ | Unpublished | Ref.^17^ |
| 16 | 11ER1-1 (ER6-1) | 50°38’36” | 120°09’38” | Xiahulin | Alkali feldspar granite | **186** | **3** | Ref.^40^ | Ref.^41^ | |
| 17 | 13ER21-1 | 51°18’42” | 120°57’58” | Northeastern moerdaoga | Granodiorite | **195** | **1** | Ref.^41^ | | |
| 18 | 13ER6-1 (12ER23-1) | 51°20’37” | 121°30’13” | Jinhe | Syenogranite | **196** | **1** | Ref.^41^ | | |
| 19 | 13TH1-1 | 52°28’12” | 125°50’39” | Baiyinna | Monzogranite | **196** | **1** | This paper | Unpublished | This paper |
| 20 | 12ER27-1 | 51°34’43” | 121°45’36” | Genhe–Mangui highway | Granodiorite | **197** | **2** | Ref.^41^ | | |
| 21 | 12ER9-1 (11ER10-1) | 50°44’37” | 119°31’56” | Qika | Granodiorite | **202** | **2** | Ref.^41^ | | |
| 22 | 11ER13-1 | 50°46’27” | 119°31’53” | Qika–Baka highway | Monzogranite | **203** | **3** | Ref.^41^ | Unpublished | Ref.^41^ |
| 23 | 12ER17-1 | 51°18’47” | 120°25’43” | Moerdaoga | Monzogranite | **205** | **2** | Ref.^41^ | | |
| 24 | 12ER19-1 | 51°29’21” | 120°48’47” | Moerdaoga | Syenogranite | **205** | **1** | Ref.^41^ | | |
| 25 | 14ER18-1 | 52°53’07” | 122°19’44” | Mohe | Syenogranite | **205** | **1** | This paper | Unpublished | This paper |
| 26 | 18ER12-1 | 49°57’25” | 118°53’42” | Southern Badaguan | Granitic gneiss | **206** | **1** | This paper | — | This paper |
| 27 | 12ER34-1 | 52°12’24” | 121°39’09” | Mangui | Syenogranite | **206** | **1** | Ref.^41^ | | |
| 28 | 11ER26-1 (12ER15-1) | 51°21’14” | 120°18’28” | Moerdaoga | Monzogranite | **206** | **2** | Ref.^41^ | | |
| 29 | 12ER16-3 (11ER27-1) | 51°19’06” | 120°22’00” | Moerdaoga | Monzogranite | **206** | **2** | Ref.^41^ | | |
| 30 | 18ER19-1 | 53°06’03” | 122°06’22” | Mohe | Syenogranite | **211** | **1** | This paper | Unpublished | This paper |
| 31 | 11ER9-1 (13ER45-1) | 50°42’25” | 119°34’09” | Enhe-Manzhouli highway | Syenogranite | **224** | **2** | Ref.^41^ | | |
| 32 | 13ER46-1 | 49°59’02” | 119°08’23” | Badaguan Cu-Mo deposit | Granodiorite | **228** | **2** | Ref.^41^ | | |
| 33 | ER12-1 (ER22-1) | 50°51’39” | 120°01’31” | Enhe–Shiwei highway | Syenogranite | **229** | **4** | Ref.^40^ | Ref.^41^ | |
| 34 | 15ER12-1 | 49°47’32” | 119°55’37” | Qiyi pasture | Monzogranite | **238** | **1** | Ref.^42^ | | This paper |
| 35 | 15ER4-1 | 51°32’57” | 120°44’54” | Northern Moerdaoga | Monzogranite | **239** | **3** | Ref.^42^ | | |
| 36 | ER15-1 (11ER5-1) | 50°47’29” | 120°05’36” | Shanghulin–Enhe highway | Granodiorite | **241** | **1** | Ref.^40^ | Ref.^43^ | |
| 37 | 11ER17-1 (12ER10-1) | 51°03’14” | 120°02’19” | Enhe–Shiwei highway | Syenogranite | **242** | **3** | Ref.^43^ | | |
| 38 | 13ER31-1 | 51°51’46” | 120°55’52” | Xiniuerhe | Granodiorite | **246** | **1** | Ref.^41^ | — | Ref.^41^ |
| 39 | 11ER18-1 (12ER11-1) | 51°11’18” | 120°02’01” | Enhe–Shiwei highway | Monzogranite | **247** | **2** | Ref.^43^ | | |
| 40 | 14ER16-1 | 53°08’59” | 121°52’18” | Mohe–Luoguhe highway | Granodiorite | **256** | **1** | Ref.^42^ | | |
| 41 | 15ER1-1 | 49°39’13” | 119°45’45” | Halatusumu | Granodiorite | **303** | **2** | Ref.^42^ | | |
| 42 | 12ER36-1 | 51°40’04” | 121°49’28” | Alongshan | Gneissic monzogranite | **455** | **10** | Ref.^44^ | | |
| 43 | 18ER23-1 | 52°23’28” | 125°52’34” | Shibazhan-Huma highway | Syenogranite | **460** | **1** | This paper | Unpublished | This paper |
| 44 | 14ER10-1 | 52°25’10” | 123°36’53” | Southern Pangu | Gneissic monzogranite | **465** | **5** | This paper | Unpublished | This paper |
| 45 | 14ER6-1 (13TH9-1) | 52°21’43” | 124°47’44” | Tahe | Alkali feldspar granite | **476** | **4** | This paper | Unpublished | This paper |
| 46 | 14ER15-1 | 52°59’23” | 122°30’27” | Mohe | Monzogranite | **479** | **2** | This paper | Unpublished | This paper |
| 47 | 14ER7-5 | 52°21’12” | 124°42’22” | Tahe | Monzogranite | **479** | **3** | This paper | Unpublished | This paper |
| 48 | 14ER5-1 | 52°25’03” | 125°05’55” | Shibazhan | Monzogranite | **481** | **3** | This paper | Unpublished | This paper |
| 49 | 13ER30-1 | 51°48’43” | 120°56’17” | Genhe–Baiyinna highway | Gneissic monzogranite | **482** | **3** | This paper | Unpublished | This paper |
| 50 | ER13-1 (ER23-1) | 50°58’44” | 120°02’22” | Shanghulin–Enhe highway | Syenogranite | **737** | **5** | Ref.^36^ | | |
| 51 | 12ER7-1 | 50°29’31” | 119°15’13” | Northern Wuka village | Biotite monzogranite | **737** | **7** | Ref.^45^ | | |
| 52 | 11ER24-1 (12ER14-1) | 51°18’41” | 120°15’38” | Eastern Shiwei | Monzogranite | **762** | **6** | Ref.^36^ | Ref.^45^ | Ref.^36^ |
| 53 | 14ER17-1 | 53°04’47” | 122°21’33” | Mohe | Syenogranite | **791** | **5** | Ref.^45^ | | |
| 54 | ER24-1 | 51°17’19” | 120°08’33” | Labudalin–Moerdaoga highway | Syenogranite | **792** | **4** | Ref.^36^ | | |
| 55 | 11ER23-1 | 51°17’19” | 120°08’33” | Labudalin–Moerdaoga highway | Syenogranite | **792** | **7** | Ref.^36^ | | |
| 56 | 14ER13-1 | 52°41’08” | 123°10’27” | Southern Amuer | Biotite monzogranite | **793** | **4** | Ref.^45^ | | |
| 57 | 13ER41-1 | 51°17’46” | 119°58’33” | Shiwei | Granodiorite | **794** | **4** | Ref.^46^ | Unpublished | Ref.^45^ |
| 58 | 13ER43-1 | 50°49’33” | 119°53’44” | Enhe village | Syenogranite | **794** | **4** | Ref.^46^ | — | Ref.^45^ |
| 59 | 13ER44-1 | 50°47’49” | 119°52’44” | Enhe village | Syenogranite | **794** | **4** | Ref.^46^ | Unpublished | Ref.^45^ |
| 60 | 14ER11-1 | 52°26’40” | 123°39’58” | Northern Bishui | Alkali feldspar granite | **795** | **4** | Ref.^45^ | | |
| 61 | 13ER12-1 | 52°04’20” | 122°04’18” | Northern Mangui | Monzogranite | **846** | **5** | Ref.^45^ | — | Ref.^45^ |
| 62 | 13ER13-1 | 52°03’26” | 122°05’33” | Northeastern Mangui | Syenogranite | **846** | **5** | Ref.^45^ | | |
| 63 | 12ER28-1 | 52°04’20” | 122°04’18” | Northern Mangui | Biotite monzogranite | **850** | **9** | Ref.^45^ | | |
| 64 | ER7-1 (ER21-1) | 50°47’24” | 120°05’53” | Shanghulin–Enhe highway | Syenogranite | **851** | **6** | Ref.^36^ | | |
| 65 | 14ER3-6 | 52°16’50” | 125°29’57” | Southern Shibazhan | Granodiorite | **1785** | **45** | This paper | Unpublished | This paper |
| 66 | 14ER3-1 | 52°16’50” | 125°29’57” | Southern Shibazhan | Gneiss | **1860** | **20** | This paper | Unpublished | This paper |

**References**

1. Sun, C. Y., Tang, J., Xu, W. L, Li, Y. & Zhao, S. Crustal accretion and reworking processes of micro-continental massifs within orogenic belt: A case study of the Erguna Massif, NE China. *Sci. China Earth Sci*. **60**(7), 1256–1267, https://doi.org/10.1007/s11430-016-9033-5 (2017).
2. Tang, J. et al. Geochronology and geochemistry of Neoproterozoic magmatism in the Erguna Massif, NE China: Petrogenesis and implications for the breakup of the Rodinia supercontinent. *Precambrian Res*. **224**, 597–611, https://doi.org/10.1016/j.precamres.2012.10.019 (2013).
3. Tang, J., Xu, W. L., Wang, F., Zhao, S. & Li, Y. Geochronology, geochemistry, and deformation history of Late Jurassic-Early Cretaceous intrusive rocks in the Erguna Massif, NE China: Constraints on the late Mesozoic tectonic evolution of the Mongol-Okhotsk orogenic belt. *Tectonophysics*. **658**, 91–110, https://doi.org/10.1016/j.tecto.2015.07.012 (2015).
4. Wang, W., Xu, W. L., Wang, F. & Meng, E. Zircon U-Pb chronology and assemblages of Mesozoic granitoids in the Manzhouli-Erguna area, NE China: Constraints on the regional tectonic evolution. *Geol. J. China Uni*. **18**(1), 88–105 (2012).
5. Tang, J., Xu, W. L., Wang, F., Zhao, S. & Wang, W. Early Mesozoic southwards subduction history of the Mongol-Okhotsk oceanic plate: Evidence from geochronology and geochemistry of Early Mesozoic intrusive rocks in the Erguna massif, NE China. *Gondwana Res*. **31**, 218–240, https://doi.org/10.1016/j.gr.2014.12.010 (2016).
6. Li, Y. et al. Geochronology and geochemistry of late Paleozoic–early Mesozoic igneous rocks of the Erguna Massif, NE China: Implications for the early evolution of the Mongol-Okhotsk tectonic regime. *J. Asian Earth Sci*. **144**, 205–244, https://doi.org/10.1016/j.jseaes.2016.12.005 (2017).
7. Tang, J. et al. Geochronology and geochemistry of Early-Middle Triassic magmatism in the Erguna Massif, NE China: Constraints on the tectonic evolution of the Mongol-Okhotsk Ocean. *Lithos*. **184–187**, 1–16, https://doi.org/10.1016/j.lithos.2013.10.024 (2014).
8. Zhao, S., Xu, W. L., Wang, W., Tang, J. & Zhang, Y. H. Geochronology and geochemistry of Middle-Late Ordovician granites and gabbros in the Erguna region, NE China: Implications for the tectonic evolution of the Erguna Massif. *J. Earth Sci-China*. **25**(5), 841–853, https://doi.org/10.1007/s12583-014-0476-9 (2014).
9. Zhao, S., Xu, W. L., Tang, J., Li, Y. & Guo, P. Neoproterozoic magmatic events and tectonic attribution of the Erguna Massif: Constraints from geochronological, geochemical and Hf isotopic data of intrusive rocks. *Earth Sci*. **41**(11), 1803–1829, https://doi.org/10.3799/dqkx.2016.550 (2016).
10. Zhao, S. et al. Neoproterozoic magmatisms in the Erguna massif, NE China: Evidence from zircon U-Pb geochronology. *Geotectonica et Metallogenia*. **40**(3), 559–573 (2016).

**Supplementary Table S2**. Zircon U-Pb dating results for the Proterozoic–Phanerozoic granitoids in the Erguna Massif

| Sample | ^207^Pb/^206^Pb | | ^207^Pb/^235^U | | ^206^Pb/^238^U | | ^207^Pb/^206^Pb | | ^207^Pb/^235^U | | ^206^Pb/^238^U | |
| --- | --- | --- | --- | --- | --- | --- | --- | --- | --- | --- | --- | --- |
|  | Ratio | 1σ | Ratio | 1σ | Ratio | 1σ | Age (Ma) | 1σ | Age (Ma) | 1σ | Age (Ma) | 1σ |
| 11ER16-6-01 | 0.06490 | 0.00605 | 0.06490 | 0.00605 | 0.02522 | 0.00059 | 771 | 146 | 202 | 16 | 161 | 4 |
| 11ER16-6-02 | 0.05822 | 0.00417 | 0.05822 | 0.00417 | 0.02119 | 0.00035 | 538 | 162 | 159 | 10 | 135 | 2 |
| 11ER16-6-03 | 0.04918 | 0.00117 | 0.04918 | 0.00117 | 0.01965 | 0.00016 | 157 | 57 | 127 | 3 | 125 | 1 |
| 11ER16-6-04 | 0.06462 | 0.00266 | 0.06462 | 0.00266 | 0.07646 | 0.00099 | 762 | 89 | 528 | 16 | 475 | 6 |
| 11ER16-6-05 | 0.06998 | 0.00211 | 0.06998 | 0.00211 | 0.03045 | 0.00040 | 928 | 49 | 264 | 8 | 193 | 3 |
| 11ER16-6-06 | 0.05505 | 0.00211 | 0.05505 | 0.00211 | 0.02447 | 0.00031 | 414 | 64 | 173 | 6 | 156 | 2 |
| 11ER16-6-07 | 0.05156 | 0.00155 | 0.05156 | 0.00155 | 0.02103 | 0.00025 | 266 | 56 | 143 | 4 | 134 | 2 |
| 11ER16-6-08 | 0.04996 | 0.00145 | 0.04996 | 0.00145 | 0.01938 | 0.00027 | 193 | 43 | 128 | 4 | 124 | 2 |
| 11ER16-6-09 | 0.04741 | 0.00189 | 0.04741 | 0.00189 | 0.01973 | 0.00033 | 70 | 57 | 123 | 4 | 126 | 2 |
| 11ER16-6-10 | 0.06110 | 0.00695 | 0.06110 | 0.00695 | 0.01929 | 0.00029 | 643 | 255 | 153 | 16 | 123 | 2 |
| MZ14-2-01 | 0.04902 | 0.00221 | 0.14669 | 0.00640 | 0.02171 | 0.00019 | 149 | 84 | 139 | 6 | 138 | 1 |
| MZ14-2-02 | 0.06791 | 0.00386 | 0.19361 | 0.01079 | 0.02068 | 0.00023 | 866 | 121 | 180 | 9 | 132 | 1 |
| MZ14-2-03 | 0.06116 | 0.00217 | 0.17201 | 0.00577 | 0.02040 | 0.00020 | 645 | 55 | 161 | 5 | 130 | 1 |
| MZ14-2-04 | 0.05865 | 0.00187 | 0.16772 | 0.00523 | 0.02074 | 0.00013 | 554 | 71 | 157 | 5 | 132.3 | 0.8 |
| MZ14-2-05 | 0.05043 | 0.00178 | 0.15205 | 0.00509 | 0.02187 | 0.00020 | 215 | 61 | 144 | 4 | 139 | 1 |
| MZ14-2-06 | 0.05514 | 0.00116 | 0.15971 | 0.00299 | 0.02101 | 0.00014 | 418 | 30 | 150 | 3 | 134 | 0.9 |
| MZ14-2-08 | 0.05178 | 0.00106 | 0.14998 | 0.00296 | 0.02101 | 0.00011 | 275 | 48 | 142 | 3 | 134 | 0.7 |
| MZ14-2-09 | 0.04949 | 0.00274 | 0.14673 | 0.00804 | 0.02150 | 0.00018 | 171 | 127 | 139 | 7 | 137 | 1 |
| MZ14-2-10 | 0.04947 | 0.00221 | 0.14689 | 0.00648 | 0.02154 | 0.00016 | 170 | 104 | 139 | 6 | 137 | 1 |
| MZ14-2-12 | 0.05526 | 0.00112 | 0.15714 | 0.00306 | 0.02062 | 0.00012 | 423 | 46 | 148 | 3 | 131.6 | 0.8 |
| MZ14-2-13 | 0.05450 | 0.00208 | 0.16125 | 0.00602 | 0.02146 | 0.00017 | 392 | 88 | 152 | 5 | 137 | 1 |
| MZ14-2-13 | 0.04908 | 0.00153 | 0.14194 | 0.00433 | 0.02098 | 0.00014 | 151 | 74 | 135 | 4 | 133.4 | 0.9 |
| 12ER8-1-01 | 0.04843 | 0.00352 | 0.13175 | 0.00913 | 0.01960 | 0.00033 | 121 | 123 | 126 | 8 | 125 | 2 |
| 12ER8-1-02 | 0.05356 | 0.00266 | 0.15952 | 0.00728 | 0.02177 | 0.00028 | 353 | 80 | 150 | 6 | 139 | 2 |
| 12ER8-1-03 | 0.06634 | 0.00246 | 0.19865 | 0.00703 | 0.02169 | 0.00027 | 817 | 53 | 184 | 6 | 138 | 2 |
| 12ER8-1-04 | 0.05944 | 0.00251 | 0.18058 | 0.00746 | 0.02206 | 0.00028 | 583 | 68 | 169 | 6 | 141 | 2 |
| 12ER8-1-05 | 0.05394 | 0.00346 | 0.14362 | 0.00916 | 0.01958 | 0.00043 | 369 | 104 | 136 | 8 | 125 | 3 |
| 12ER8-1-06 | 0.04940 | 0.00247 | 0.14798 | 0.00730 | 0.02179 | 0.00038 | 167 | 82 | 140 | 6 | 139 | 2 |
| 12ER8-1-07 | 0.04726 | 0.00495 | 0.13162 | 0.01465 | 0.01964 | 0.00046 | 63.0 | 202 | 126 | 13 | 125 | 3 |
| 12ER8-1-08 | 0.04712 | 0.00262 | 0.14800 | 0.00843 | 0.02259 | 0.00029 | 55.0 | 101 | 140 | 7 | 144 | 2 |
| 12ER8-1-09 | 0.05223 | 0.00445 | 0.15986 | 0.01344 | 0.02220 | 0.00031 | 295 | 195 | 151 | 12 | 142 | 2 |
| 12ER8-1-10 | 0.05238 | 0.00457 | 0.15647 | 0.01327 | 0.02166 | 0.00044 | 302 | 199 | 148 | 12 | 138 | 3 |
| 12ER8-1-11 | 0.05766 | 0.00289 | 0.15673 | 0.00781 | 0.01953 | 0.00032 | 517 | 81 | 148 | 7 | 125 | 2 |
| 12ER8-1-12 | 0.04810 | 0.00162 | 0.14956 | 0.00501 | 0.02240 | 0.00027 | 104 | 56 | 142 | 4 | 143 | 2 |
| 12ER8-1-13 | 0.05027 | 0.00251 | 0.15360 | 0.00758 | 0.02212 | 0.00032 | 208 | 87 | 145 | 7 | 141 | 2 |
| 12ER8-1-14 | 0.04958 | 0.00232 | 0.15166 | 0.00708 | 0.02198 | 0.00029 | 175 | 84 | 143 | 6 | 140 | 2 |
| 12ER8-1-15 | 0.05006 | 0.00264 | 0.14729 | 0.00746 | 0.02154 | 0.00033 | 198 | 89 | 140 | 7 | 137 | 2 |
| 12ER8-1-16 | 0.05050 | 0.00205 | 0.15077 | 0.00617 | 0.02148 | 0.00028 | 218 | 71 | 143 | 5 | 137 | 2 |
| 12ER8-1-17 | 0.05378 | 0.00202 | 0.16438 | 0.00611 | 0.02199 | 0.00027 | 362 | 61 | 155 | 5 | 140 | 2 |
| 12ER8-1-18 | 0.04752 | 0.00316 | 0.14528 | 0.00920 | 0.02260 | 0.00043 | 76.0 | 104 | 138 | 8 | 144 | 3 |
| 12ER8-1-19 | 0.04605 | 0.00400 | 0.13995 | 0.01175 | 0.02204 | 0.00049 |  | 192 | 133 | 10 | 141 | 3 |
| 12ER8-1-20 | 0.05899 | 0.00279 | 0.17913 | 0.00822 | 0.02196 | 0.00038 | 567 | 70 | 167 | 7 | 140 | 2 |
| 12ER8-1-21 | 0.06200 | 0.00306 | 0.19174 | 0.00929 | 0.02239 | 0.00034 | 674 | 78 | 178 | 8 | 143 | 2 |
| 12ER8-1-22 | 0.05459 | 0.00421 | 0.16660 | 0.01229 | 0.02244 | 0.00040 | 395 | 134 | 156 | 11 | 143 | 3 |
| 17ER9-1-01 | 0.05014 | 0.01036 | 0.13493 | 0.02328 | 0.02186 | 0.00087 | 202 | 288 | 129 | 21 | 139 | 5 |
| 17ER9-1-02 | 0.05933 | 0.00948 | 0.18260 | 0.02386 | 0.02192 | 0.00080 | 579 | 224 | 170 | 20 | 140 | 5 |
| 17ER9-1-03 | 0.05660 | 0.00569 | 0.16164 | 0.01360 | 0.02183 | 0.00048 | 476 | 148 | 152 | 12 | 139 | 3 |
| 17ER9-1-04 | 0.04897 | 0.00357 | 0.14830 | 0.00921 | 0.02221 | 0.00040 | 147 | 107 | 140 | 8 | 142 | 3 |
| 17ER9-1-05 | 0.04916 | 0.00358 | 0.15049 | 0.00968 | 0.02224 | 0.00037 | 156 | 115 | 142 | 9 | 142 | 2 |
| 17ER9-1-06 | 0.04892 | 0.00376 | 0.14332 | 0.00904 | 0.02223 | 0.00048 | 144 | 103 | 136 | 8 | 142 | 3 |
| 17ER9-1-07 | 0.04897 | 0.00661 | 0.12733 | 0.01018 | 0.02218 | 0.00055 | 147 | 133 | 122 | 9 | 141 | 3 |
| 17ER9-1-08 | 0.06792 | 0.00384 | 0.20599 | 0.01072 | 0.02227 | 0.00030 | 866 | 86 | 190 | 9 | 142 | 2 |
| 17ER9-1-09 | 0.04895 | 0.00529 | 0.14945 | 0.01303 | 0.02233 | 0.00049 | 145 | 154 | 141 | 12 | 142 | 3 |
| 17ER9-1-10 | 0.04771 | 0.00172 | 0.14459 | 0.00497 | 0.02210 | 0.00023 | 85 | 60 | 137 | 4 | 141 | 1 |
| 17ER9-1-11 | 0.05729 | 0.00309 | 0.17140 | 0.00811 | 0.02209 | 0.00029 | 503 | 81 | 161 | 7 | 141 | 2 |
| 17ER9-1-12 | 0.05219 | 0.01063 | 0.16883 | 0.03336 | 0.02198 | 0.00089 | 294 | 335 | 158 | 29 | 140 | 6 |
| 17ER9-1-13 | 0.04992 | 0.00434 | 0.14950 | 0.01179 | 0.02182 | 0.00036 | 191 | 147 | 141 | 10 | 139 | 2 |
| 17ER9-1-14 | 0.05543 | 0.00712 | 0.16047 | 0.01501 | 0.02182 | 0.00059 | 430 | 161 | 151 | 13 | 139 | 4 |
| 17ER9-1-15 | 0.05179 | 0.00372 | 0.15724 | 0.01084 | 0.02230 | 0.00037 | 276 | 127 | 148 | 10 | 142 | 2 |
| 17ER9-1-16 | 0.05003 | 0.00420 | 0.14772 | 0.00966 | 0.02210 | 0.00054 | 196 | 105 | 140 | 9 | 141 | 3 |
| 17ER9-1-17 | 0.04930 | 0.00179 | 0.15175 | 0.00537 | 0.02235 | 0.00020 | 162 | 66 | 143 | 5 | 142 | 1 |
| 17ER9-1-18 | 0.04924 | 0.00448 | 0.14317 | 0.01052 | 0.02210 | 0.00042 | 159 | 130 | 136 | 9 | 141 | 3 |
| 17ER9-1-19 | 0.04935 | 0.00388 | 0.14987 | 0.01089 | 0.02206 | 0.00039 | 165 | 131 | 142 | 10 | 141 | 2 |
| 17ER9-1-20 | 0.04901 | 0.00449 | 0.14720 | 0.01086 | 0.02222 | 0.00045 | 148 | 128 | 139 | 10 | 142 | 3 |
| DB-01-01 | 0.04833 | 0.00169 | 0.14798 | 0.00516 | 0.02231 | 0.00039 | 115 | 50 | 140 | 5 | 142 | 2 |
| DB-01-02 | 0.05227 | 0.00375 | 0.17004 | 0.01381 | 0.02306 | 0.00053 | 297 | 142 | 159 | 12 | 147 | 3 |
| DB-01-03 | 0.05206 | 0.00351 | 0.15776 | 0.01032 | 0.02212 | 0.00036 | 288 | 120 | 149 | 9 | 141 | 2 |
| DB-01-04 | 0.05382 | 0.00323 | 0.16944 | 0.01148 | 0.02282 | 0.00051 | 364 | 112 | 159 | 10 | 145 | 3 |
| DB-01-05 | 0.05091 | 0.00196 | 0.15619 | 0.00586 | 0.02243 | 0.00028 | 237 | 63 | 147 | 5 | 143 | 2 |
| DB-01-06 | 0.06312 | 0.00352 | 0.19882 | 0.01162 | 0.02287 | 0.00047 | 712 | 89 | 184 | 10 | 146 | 3 |
| DB-01-07 | 0.04634 | 0.00461 | 0.13820 | 0.01352 | 0.02163 | 0.00039 | 16 | 208 | 131 | 12 | 138 | 2 |
| DB-01-08 | 0.04989 | 0.00203 | 0.14899 | 0.00568 | 0.02200 | 0.00027 | 190 | 66 | 141 | 5 | 140 | 2 |
| DB-01-09 | 0.04605 | 0.00318 | 0.14268 | 0.00912 | 0.02247 | 0.00058 |  | 152 | 135 | 8 | 143 | 4 |
| DB-01-10 | 0.05060 | 0.00222 | 0.15523 | 0.00654 | 0.02243 | 0.00024 | 223 | 78 | 147 | 6 | 143 | 2 |
| DB-01-11 | 0.05128 | 0.00270 | 0.15224 | 0.00731 | 0.02211 | 0.00026 | 253 | 89 | 144 | 6 | 141 | 2 |
| DB-01-12 | 0.04929 | 0.00148 | 0.15363 | 0.00443 | 0.02277 | 0.00024 | 162 | 48 | 145 | 4 | 145 | 1 |
| DB-01-13 | 0.04770 | 0.00080 | 0.14821 | 0.00262 | 0.02252 | 0.00017 | 84 | 28 | 140 | 2 | 144 | 1 |
| DB-01-14 | 0.04950 | 0.00211 | 0.15242 | 0.00638 | 0.02264 | 0.00026 | 172 | 76 | 144 | 6 | 144 | 2 |
| DB-01-15 | 0.04847 | 0.00137 | 0.14704 | 0.00409 | 0.02204 | 0.00018 | 122 | 50 | 139 | 4 | 141 | 1 |
| DB-01-16 | 0.04916 | 0.00215 | 0.15293 | 0.00685 | 0.02268 | 0.00029 | 155 | 80 | 144 | 6 | 145 | 2 |
| 13ER48-1-01 | 0.05052 | 0.00254 | 0.15699 | 0.00712 | 0.02302 | 0.00034 | 219 | 78 | 148 | 6 | 147 | 2 |
| 13ER48-1-02 | 0.05122 | 0.00175 | 0.17000 | 0.00582 | 0.02399 | 0.00023 | 251 | 61 | 159 | 5 | 153 | 1 |
| 13ER48-1-03 | 0.04886 | 0.00337 | 0.15321 | 0.01029 | 0.02309 | 0.00033 | 141 | 124 | 145 | 9 | 147 | 2 |
| 13ER48-1-04 | 0.05905 | 0.00551 | 0.18336 | 0.01653 | 0.02290 | 0.00057 | 569 | 154 | 171 | 14 | 146 | 4 |
| 13ER48-1-05 | 0.05061 | 0.00478 | 0.15440 | 0.01296 | 0.02273 | 0.00056 | 223 | 145 | 146 | 11 | 145 | 4 |
| 13ER48-1-06 | 0.05019 | 0.00186 | 0.16633 | 0.00604 | 0.02402 | 0.00025 | 204 | 65 | 156 | 5 | 153 | 2 |
| 13ER48-1-07 | 0.04784 | 0.00251 | 0.15510 | 0.00778 | 0.02386 | 0.00034 | 91 | 85 | 146 | 7 | 152 | 2 |
| 13ER48-1-08 | 0.05133 | 0.00166 | 0.16598 | 0.00514 | 0.02342 | 0.00022 | 256 | 54 | 156 | 4 | 149 | 1 |
| 13ER48-1-09 | 0.06493 | 0.00486 | 0.20564 | 0.01497 | 0.02297 | 0.00040 | 772 | 163 | 190 | 13 | 146 | 3 |
| 13ER48-1-10 | 0.05306 | 0.00273 | 0.17558 | 0.00865 | 0.02422 | 0.00030 | 331 | 90 | 164 | 7 | 154 | 2 |
| 13ER48-1-11 | 0.05551 | 0.00353 | 0.17181 | 0.01024 | 0.02281 | 0.00038 | 433 | 103 | 161 | 9 | 145 | 2 |
| 12ER2-1-01 | 0.05120 | 0.00820 | 0.16103 | 0.02547 | 0.02281 | 0.00059 | 250 | 325 | 152 | 22 | 145 | 4 |
| 12ER2-1-02 | 0.04835 | 0.00322 | 0.15943 | 0.01023 | 0.02396 | 0.00042 | 117 | 110 | 150 | 9 | 153 | 3 |
| 12ER2-1-03 | 0.05247 | 0.00314 | 0.17719 | 0.01033 | 0.02428 | 0.00034 | 306 | 108 | 166 | 9 | 155 | 2 |
| 12ER2-1-04 | 0.06640 | 0.00623 | 0.21258 | 0.01657 | 0.02440 | 0.00067 | 819 | 117 | 196 | 14 | 155 | 4 |
| 12ER2-1-05 | 0.05659 | 0.00544 | 0.18007 | 0.01603 | 0.02311 | 0.00048 | 476 | 162 | 168 | 14 | 147 | 3 |
| 12ER2-1-06 | 0.04830 | 0.00227 | 0.16157 | 0.00732 | 0.02420 | 0.00032 | 114 | 78 | 152 | 6 | 154 | 2 |
| 12ER2-1-07 | 0.04979 | 0.00222 | 0.16374 | 0.00723 | 0.02373 | 0.00024 | 185 | 84 | 154 | 6 | 151 | 2 |
| 12ER2-1-08 | 0.05218 | 0.00285 | 0.17546 | 0.00979 | 0.02434 | 0.00035 | 293 | 102 | 164 | 8 | 155 | 2 |
| 12ER2-1-09 | 0.05538 | 0.00246 | 0.18153 | 0.00798 | 0.02380 | 0.00030 | 428 | 75 | 169 | 7 | 152 | 2 |
| 12ER2-1-10 | 0.06569 | 0.00572 | 0.21402 | 0.01794 | 0.02390 | 0.00066 | 796 | 130 | 197 | 15 | 152 | 4 |
| 12ER2-1-11 | 0.05106 | 0.00332 | 0.16060 | 0.01005 | 0.02292 | 0.00036 | 243 | 115 | 151 | 9 | 146 | 2 |
| 12ER2-1-12 | 0.05044 | 0.00207 | 0.16599 | 0.00656 | 0.02394 | 0.00029 | 215 | 69 | 156 | 6 | 152 | 2 |
| 12ER2-1-13 | 0.06003 | 0.00422 | 0.19119 | 0.01300 | 0.02283 | 0.00056 | 605 | 105 | 178 | 11 | 146 | 4 |
| 12ER2-1-14 | 0.04916 | 0.00327 | 0.15506 | 0.00968 | 0.02314 | 0.00030 | 155 | 117 | 146 | 9 | 147 | 2 |
| 12ER2-1-15 | 0.05608 | 0.00228 | 0.18921 | 0.00757 | 0.02442 | 0.00029 | 455 | 68 | 176 | 6 | 156 | 2 |
| 12ER2-1-16 | 0.06609 | 0.00384 | 0.21536 | 0.01284 | 0.02345 | 0.00045 | 809 | 93 | 198 | 11 | 149 | 3 |
| 12ER2-1-17 | 0.05265 | 0.00318 | 0.17375 | 0.00976 | 0.02432 | 0.00037 | 314 | 100 | 163 | 8 | 155 | 2 |
| 12ER2-1-18 | 0.04890 | 0.00262 | 0.15757 | 0.00825 | 0.02321 | 0.00032 | 143 | 94 | 149 | 7 | 148 | 2 |
| 12ER2-1-19 | 0.04986 | 0.00168 | 0.16795 | 0.00592 | 0.02413 | 0.00029 | 189 | 59 | 158 | 5 | 154 | 2 |
| 12ER2-1-20 | 0.05358 | 0.00318 | 0.17760 | 0.00972 | 0.02420 | 0.00050 | 353 | 86 | 166 | 8 | 154 | 3 |
| 12ER2-1-21 | 0.04945 | 0.00196 | 0.16375 | 0.00632 | 0.02387 | 0.00026 | 169 | 70 | 154 | 6 | 152 | 2 |
| 12ER2-1-22 | 0.05440 | 0.00396 | 0.18083 | 0.01417 | 0.02433 | 0.00066 | 387 | 127 | 169 | 12 | 155 | 4 |
| 12ER2-1-23 | 0.04827 | 0.00345 | 0.16612 | 0.01214 | 0.02484 | 0.00057 | 112 | 121 | 156 | 11 | 158 | 4 |
| 12ER2-1-24 | 0.05222 | 0.00264 | 0.17537 | 0.00861 | 0.02452 | 0.00034 | 295 | 87 | 164 | 7 | 156 | 2 |
| 12ER2-1-25 | 0.04952 | 0.00267 | 0.16367 | 0.00842 | 0.02398 | 0.00028 | 173 | 97 | 154 | 7 | 153 | 2 |
| 12ER2-1-26 | 0.05712 | 0.00518 | 0.17661 | 0.01582 | 0.02242 | 0.00033 | 496 | 207 | 165 | 14 | 143 | 2 |
| 12ER2-1-27 | 0.05249 | 0.00203 | 0.17575 | 0.00664 | 0.02419 | 0.00026 | 307 | 66 | 164 | 6 | 154 | 2 |
| DB-03-01 | 0.05860 | 0.00185 | 0.19724 | 0.00862 | 0.02430 | 0.00068 | 552 | 50 | 183 | 7 | 155 | 4 |
| DB-03-02 | 0.06534 | 0.00069 | 0.21786 | 0.00747 | 0.02421 | 0.00082 | 785 | 33 | 200 | 6 | 154 | 5 |
| DB-03-03 | 0.04605 | 0.00421 | 0.15322 | 0.01380 | 0.02413 | 0.00038 |  | 198 | 145 | 12 | 154 | 2 |
| DB-03-04 | 0.07666 | 0.00276 | 0.25818 | 0.01085 | 0.02431 | 0.00034 | 1112 | 62 | 233 | 9 | 155 | 2 |
| DB-03-05 | 0.05813 | 0.00266 | 0.19607 | 0.00909 | 0.02436 | 0.00039 | 535 | 73 | 182 | 8 | 155 | 2 |
| DB-03-06 | 0.04795 | 0.00303 | 0.15784 | 0.00983 | 0.02387 | 0.00024 | 97 | 141 | 149 | 9 | 152 | 2 |
| DB-03-07 | 0.04617 | 0.00469 | 0.15193 | 0.01505 | 0.02386 | 0.00053 | 7 | 213 | 144 | 13 | 152 | 3 |
| DB-03-08 | 0.05466 | 0.00478 | 0.18568 | 0.01596 | 0.02464 | 0.00041 | 398 | 200 | 173 | 14 | 157 | 3 |
| 12ER1-2-01 | 0.05063 | 0.00214 | 0.17030 | 0.00694 | 0.02438 | 0.00033 | 224 | 69 | 160 | 6 | 155 | 2 |
| 12ER1-2-02 | 0.05405 | 0.00228 | 0.18276 | 0.00728 | 0.02447 | 0.00030 | 373 | 67 | 170 | 6 | 156 | 2 |
| 12ER1-2-03 | 0.05156 | 0.00264 | 0.21359 | 0.01069 | 0.03005 | 0.00049 | 266 | 85 | 197 | 9 | 191 | 3 |
| 12ER1-2-04 | 0.05033 | 0.00167 | 0.23841 | 0.00776 | 0.03401 | 0.00036 | 210 | 56 | 217 | 6 | 216 | 2 |
| 12ER1-2-05 | 0.04998 | 0.00188 | 0.24933 | 0.00996 | 0.03575 | 0.00061 | 194 | 61 | 226 | 8 | 226 | 4 |
| 12ER1-2-06 | 0.05916 | 0.00950 | 0.18998 | 0.02719 | 0.02392 | 0.00068 | 573 | 267 | 177 | 23 | 152 | 4 |
| 12ER1-2-07 | 0.06322 | 0.00185 | 1.21453 | 0.03776 | 0.13747 | 0.00187 | 716 | 43 | 807 | 17 | 830 | 11 |
| 12ER1-2-08 | 0.06333 | 0.00188 | 1.21180 | 0.03715 | 0.13715 | 0.00188 | 719 | 42 | 806 | 17 | 829 | 11 |
| 12ER1-2-09 | 0.05655 | 0.00343 | 0.19102 | 0.01110 | 0.02442 | 0.00039 | 474 | 100 | 177 | 9 | 156 | 2 |
| 12ER1-2-10 | 0.06246 | 0.00304 | 0.37323 | 0.01713 | 0.04307 | 0.00068 | 690 | 71 | 322 | 13 | 272 | 4 |
| 12ER1-2-11 | 0.06242 | 0.00167 | 1.29233 | 0.03291 | 0.14855 | 0.00173 | 689 | 35 | 842 | 15 | 893 | 10 |
| 12ER1-2-12 | 0.05218 | 0.00324 | 0.18213 | 0.01112 | 0.02530 | 0.00047 | 293 | 106 | 170 | 10 | 161 | 3 |
| 12ER1-2-13 | 0.04826 | 0.00162 | 0.16650 | 0.00531 | 0.02477 | 0.00026 | 112 | 55 | 156 | 5 | 158 | 2 |
| 12ER1-2-14 | 0.06063 | 0.00262 | 0.66938 | 0.02810 | 0.07942 | 0.00109 | 626 | 67 | 520 | 17 | 493 | 6 |
| 12ER1-2-15 | 0.05084 | 0.00318 | 0.16965 | 0.01022 | 0.02404 | 0.00038 | 234 | 110 | 159 | 9 | 153 | 2 |
| 12ER1-2-16 | 0.06270 | 0.00210 | 0.90473 | 0.02848 | 0.10465 | 0.00121 | 698 | 73 | 654 | 15 | 642 | 7 |
| 12ER1-2-17 | 0.04703 | 0.00218 | 0.15286 | 0.00697 | 0.02326 | 0.00030 | 51 | 75 | 144 | 6 | 148 | 2 |
| MZ23-1-01 | 0.05157 | 0.00153 | 0.22352 | 0.00528 | 0.03143 | 0.00033 | 266 | 35 | 205 | 4 | 199 | 2 |
| MZ23-1-02 | 0.04956 | 0.00246 | 0.21623 | 0.00984 | 0.03164 | 0.00044 | 174 | 80 | 199 | 8 | 201 | 3 |
| MZ23-1-03 | 0.07609 | 0.00195 | 0.33518 | 0.00615 | 0.03195 | 0.00033 | 1097 | 21 | 294 | 5 | 203 | 2 |
| MZ23-1-04 | 0.06576 | 0.00162 | 0.28628 | 0.00491 | 0.03158 | 0.00031 | 799 | 20 | 256 | 4 | 200 | 2 |
| MZ23-1-05 | 0.05016 | 0.00126 | 0.21876 | 0.00389 | 0.03163 | 0.00031 | 202 | 24 | 201 | 3 | 201 | 2 |
| MZ23-1-06 | 0.05111 | 0.00181 | 0.18986 | 0.00644 | 0.02694 | 0.00028 | 246 | 84 | 177 | 5 | 171 | 2 |
| MZ23-1-07 | 0.04926 | 0.00190 | 0.18144 | 0.00671 | 0.02671 | 0.00029 | 160 | 91 | 169 | 6 | 170 | 2 |
| MZ23-1-08 | 0.06186 | 0.00209 | 0.23231 | 0.00653 | 0.02724 | 0.00031 | 669 | 41 | 212 | 5 | 173 | 2 |
| MZ23-1-09 | 0.05872 | 0.00255 | 0.21980 | 0.00854 | 0.02715 | 0.00036 | 557 | 61 | 202 | 7 | 173 | 2 |
| MZ23-1-10 | 0.05350 | 0.00318 | 0.19960 | 0.01152 | 0.02706 | 0.00039 | 350 | 138 | 185 | 10 | 172 | 2 |
| MZ23-1-11 | 0.05316 | 0.00313 | 0.19861 | 0.01139 | 0.02710 | 0.00037 | 335 | 137 | 184 | 10 | 172 | 2 |
| MZ23-1-12 | 0.05000 | 0.00143 | 0.21357 | 0.00475 | 0.03098 | 0.00032 | 195 | 33 | 197 | 4 | 197 | 2 |
| MZ23-1-13 | 0.05791 | 0.00194 | 0.21669 | 0.00690 | 0.02714 | 0.00028 | 526 | 75 | 199 | 6 | 173 | 2 |
| MZ23-1-14 | 0.05017 | 0.00150 | 0.21676 | 0.00609 | 0.03133 | 0.00032 | 203 | 71 | 199 | 5 | 199 | 2 |
| MZ23-1-15 | 0.04952 | 0.00142 | 0.18481 | 0.00409 | 0.02707 | 0.00028 | 173 | 32 | 172 | 4 | 172 | 2 |
| MZ23-1-16 | 0.06245 | 0.00220 | 0.23550 | 0.00700 | 0.02735 | 0.00032 | 690 | 43 | 215 | 6 | 174 | 2 |
| MZ23-1-17 | 0.05195 | 0.00122 | 0.22494 | 0.00342 | 0.03140 | 0.00030 | 283 | 18 | 206 | 3 | 199 | 2 |
| MZ23-1-18 | 0.06292 | 0.00173 | 0.27639 | 0.00569 | 0.03186 | 0.00033 | 706 | 26 | 248 | 5 | 202 | 2 |
| MZ23-1-19 | 0.05144 | 0.00191 | 0.22297 | 0.00793 | 0.03144 | 0.00034 | 260 | 87 | 204 | 7 | 200 | 2 |
| MZ23-1-20 | 0.05070 | 0.00170 | 0.19079 | 0.00607 | 0.02729 | 0.00028 | 227 | 79 | 177 | 5 | 174 | 2 |
| 12ER31-1-01 | 0.05095 | 0.00269 | 0.21426 | 0.01169 | 0.03038 | 0.00053 | 239 | 94 | 197 | 10 | 193 | 3 |
| 12ER31-1-02 | 0.05786 | 0.00300 | 0.23592 | 0.01219 | 0.02935 | 0.00041 | 525 | 89 | 215 | 10 | 186 | 3 |
| 12ER31-1-03 | 0.06482 | 0.00251 | 0.28607 | 0.01101 | 0.03183 | 0.00042 | 769 | 59 | 255 | 9 | 202 | 3 |
| 12ER31-1-04 | 0.05235 | 0.00219 | 0.21557 | 0.00900 | 0.03000 | 0.00035 | 301 | 74 | 198 | 8 | 191 | 2 |
| 12ER31-1-05 | 0.04605 | 0.00303 | 0.18858 | 0.01174 | 0.02970 | 0.00064 |  | 145 | 175 | 10 | 189 | 4 |
| 12ER31-1-06 | 0.04605 | 0.00760 | 0.19492 | 0.03181 | 0.03070 | 0.00078 |  | 290 | 181 | 27 | 195 | 5 |
| 12ER31-1-07 | 0.05331 | 0.00289 | 0.22464 | 0.01212 | 0.03055 | 0.00053 | 342 | 91 | 206 | 10 | 194 | 3 |
| 12ER31-1-08 | 0.05250 | 0.00352 | 0.21698 | 0.01390 | 0.03031 | 0.00044 | 307 | 120 | 199 | 12 | 192 | 3 |
| 12ER31-1-09 | 0.04838 | 0.00393 | 0.20989 | 0.01682 | 0.03146 | 0.00043 | 118 | 183 | 193 | 14 | 200 | 3 |
| 12ER31-1-10 | 0.05160 | 0.00338 | 0.21183 | 0.01340 | 0.03024 | 0.00047 | 268 | 117 | 195 | 11 | 192 | 3 |
| 12ER31-1-11 | 0.05713 | 0.00281 | 0.21564 | 0.01083 | 0.02707 | 0.00042 | 497 | 83 | 198 | 9 | 172 | 3 |
| 12ER31-1-12 | 0.05404 | 0.00319 | 0.22973 | 0.01373 | 0.03045 | 0.00048 | 373 | 106 | 210 | 11 | 193 | 3 |
| 12ER31-1-13 | 0.05295 | 0.00264 | 0.21963 | 0.01047 | 0.03014 | 0.00038 | 327 | 85 | 202 | 9 | 191 | 2 |
| 12ER31-1-14 | 0.05339 | 0.00248 | 0.22259 | 0.01000 | 0.03012 | 0.00044 | 346 | 75 | 204 | 8 | 191 | 3 |
| 12ER31-1-15 | 0.05334 | 0.00217 | 0.21303 | 0.00839 | 0.02877 | 0.00031 | 343 | 70 | 196 | 7 | 183 | 2 |
| 12ER31-1-17 | 0.05245 | 0.00196 | 0.22044 | 0.00806 | 0.03025 | 0.00033 | 305 | 63 | 202 | 7 | 192 | 2 |
| 12ER31-1-18 | 0.06420 | 0.00485 | 0.24327 | 0.01773 | 0.02740 | 0.00055 | 748 | 121 | 221 | 14 | 174 | 3 |
| 12ER31-1-19 | 0.04866 | 0.00810 | 0.18076 | 0.02987 | 0.02694 | 0.00052 | 131 | 310 | 169 | 26 | 171 | 3 |
| 12ER31-1-20 | 0.07182 | 0.00799 | 0.26859 | 0.02881 | 0.02726 | 0.00049 | 981 | 193 | 242 | 23 | 173 | 3 |
| 12ER31-1-22 | 0.05584 | 0.00403 | 0.20568 | 0.01421 | 0.02689 | 0.00060 | 446 | 114 | 190 | 12 | 171 | 4 |
| 12ER31-1-24 | 0.04750 | 0.00357 | 0.17549 | 0.01303 | 0.02680 | 0.00033 | 74 | 168 | 164 | 11 | 170 | 2 |
| 12ER31-1-25 | 0.05690 | 0.00227 | 0.21404 | 0.00857 | 0.02705 | 0.00045 | 488 | 59 | 197 | 7 | 172 | 3 |
| 14ER14-1-01 | 0.05155 | 0.00277 | 0.19681 | 0.01022 | 0.02773 | 0.00036 | 265 | 96 | 182 | 9 | 176 | 2 |
| 14ER14-1-02 | 0.05169 | 0.00183 | 0.19824 | 0.00709 | 0.02770 | 0.00027 | 272 | 64 | 184 | 6 | 176 | 2 |
| 14ER14-1-03 | 0.05199 | 0.00219 | 0.19860 | 0.00821 | 0.02781 | 0.00033 | 285 | 72 | 184 | 7 | 177 | 2 |
| 14ER14-1-04 | 0.04987 | 0.00294 | 0.19079 | 0.01136 | 0.02779 | 0.00035 | 189 | 113 | 177 | 10 | 177 | 2 |
| 14ER14-1-05 | 0.05035 | 0.00271 | 0.19053 | 0.00924 | 0.02771 | 0.00039 | 211 | 87 | 177 | 8 | 176 | 2 |
| 14ER14-1-06 | 0.05412 | 0.00280 | 0.20750 | 0.01065 | 0.02776 | 0.00038 | 376 | 91 | 191 | 9 | 176 | 2 |
| 14ER14-1-07 | 0.05101 | 0.00170 | 0.19752 | 0.00666 | 0.02786 | 0.00028 | 241 | 59 | 183 | 6 | 177 | 2 |
| 14ER14-1-08 | 0.05020 | 0.00241 | 0.19181 | 0.00881 | 0.02780 | 0.00033 | 204 | 85 | 178 | 8 | 177 | 2 |
| 14ER14-1-09 | 0.04987 | 0.00249 | 0.19149 | 0.00948 | 0.02771 | 0.00032 | 189 | 93 | 178 | 8 | 176 | 2 |
| 14ER14-1-10 | 0.05388 | 0.00224 | 0.20939 | 0.00900 | 0.02787 | 0.00029 | 366 | 78 | 193 | 8 | 177 | 2 |
| MZ18-2-01 | 0.05004 | 0.00353 | 0.19803 | 0.01323 | 0.02869 | 0.00052 | 197 | 119 | 183 | 11 | 182 | 3 |
| MZ18-2-05 | 0.04996 | 0.00178 | 0.19818 | 0.00609 | 0.02878 | 0.00033 | 193 | 50 | 184 | 5 | 183 | 2 |
| MZ18-2-03 | 0.04935 | 0.00161 | 0.19214 | 0.00521 | 0.02824 | 0.00030 | 164 | 43 | 178 | 4 | 180 | 2 |
| MZ18-2-04 | 0.04792 | 0.00341 | 0.17975 | 0.01245 | 0.02720 | 0.00044 | 95 | 160 | 168 | 11 | 173 | 3 |
| MZ18-2-03 | 0.04935 | 0.00161 | 0.19214 | 0.00521 | 0.02824 | 0.00030 | 164 | 43 | 178 | 4 | 180 | 2 |
| MZ18-2-06 | 0.04983 | 0.00204 | 0.19339 | 0.00704 | 0.02816 | 0.00035 | 187 | 61 | 180 | 6 | 179 | 2 |
| MZ18-2-07 | 0.05042 | 0.00337 | 0.19397 | 0.01226 | 0.02791 | 0.00049 | 214 | 112 | 180 | 10 | 177 | 3 |
| MZ18-2-08 | 0.05765 | 0.00165 | 0.22745 | 0.00523 | 0.02863 | 0.00030 | 516 | 32 | 208 | 4 | 182 | 2 |
| MZ18-2-09 | 0.07601 | 0.00373 | 0.28900 | 0.01367 | 0.02758 | 0.00036 | 1095 | 101 | 258 | 11 | 175 | 2 |
| MZ18-2-10 | 0.05933 | 0.00330 | 0.22700 | 0.01178 | 0.02776 | 0.00046 | 579 | 84 | 208 | 10 | 177 | 3 |
| MZ18-2-11 | 0.05693 | 0.00394 | 0.22051 | 0.01445 | 0.02810 | 0.00055 | 489 | 110 | 202 | 12 | 179 | 3 |
| MZ18-2-12 | 0.04966 | 0.00327 | 0.19150 | 0.01196 | 0.02797 | 0.00050 | 179 | 109 | 178 | 10 | 178 | 3 |
| MZ18-2-13 | 0.04996 | 0.00248 | 0.19152 | 0.00880 | 0.02781 | 0.00040 | 193 | 80 | 178 | 7 | 177 | 3 |
| MZ18-2-14 | 0.05008 | 0.00128 | 0.19665 | 0.00392 | 0.02848 | 0.00028 | 199 | 28 | 182 | 3 | 181 | 2 |
| MZ18-2-15 | 0.04939 | 0.00153 | 0.19311 | 0.00507 | 0.02836 | 0.00031 | 166 | 41 | 179 | 4 | 180 | 2 |
| ZKS1-1-01 | 0.04959 | 0.00121 | 0.19632 | 0.00462 | 0.02883 | 0.00028 | 176 | 37 | 182 | 4 | 183 | 2 |
| ZKS1-1-12 | 0.05059 | 0.00133 | 0.23108 | 0.00669 | 0.03304 | 0.00040 | 222 | 44 | 211 | 6 | 210 | 3 |
| ZKS1-1-13 | 0.05588 | 0.00113 | 0.25709 | 0.00573 | 0.03328 | 0.00024 | 448 | 36 | 232 | 5 | 211 | 2 |
| ZKS1-1-14 | 0.05246 | 0.00134 | 0.24187 | 0.00914 | 0.03333 | 0.00080 | 305 | 45 | 220 | 7 | 211 | 5 |
| ZKS1-1-15 | 0.05264 | 0.00148 | 0.24201 | 0.00734 | 0.03338 | 0.00056 | 313 | 39 | 220 | 6 | 212 | 3 |
| ZKS1-1-16 | 0.05247 | 0.00107 | 0.24244 | 0.00472 | 0.03351 | 0.00030 | 306 | 28 | 220 | 4 | 212 | 2 |
| ZKS1-1-17 | 0.05252 | 0.00067 | 0.24223 | 0.00384 | 0.03346 | 0.00040 | 308 | 17 | 220 | 3 | 212 | 2 |
| ZKS1-1-18 | 0.05213 | 0.00097 | 0.24159 | 0.00514 | 0.03362 | 0.00050 | 291 | 24 | 220 | 4 | 213 | 3 |
| ZKS1-1-19 | 0.05215 | 0.00114 | 0.24176 | 0.00555 | 0.03360 | 0.00032 | 292 | 35 | 220 | 5 | 213 | 2 |
| ZKS1-1-20 | 0.05269 | 0.00109 | 0.24393 | 0.00561 | 0.03359 | 0.00045 | 316 | 29 | 222 | 5 | 213 | 3 |
| ZKS1-1-21 | 0.05202 | 0.00139 | 0.27107 | 0.00670 | 0.03792 | 0.00062 | 286 | 29 | 244 | 5 | 240 | 4 |
| ZKS1-1-22 | 0.05044 | 0.00108 | 0.26455 | 0.00742 | 0.03793 | 0.00060 | 215 | 37 | 238 | 6 | 240 | 4 |
| ZKS1-1-23 | 0.05305 | 0.00350 | 0.24413 | 0.01572 | 0.03338 | 0.00047 | 331 | 153 | 222 | 13 | 212 | 3 |
| ZKS1-1-24 | 0.05312 | 0.00162 | 0.24709 | 0.00777 | 0.03354 | 0.00037 | 334 | 51 | 224 | 6 | 213 | 2 |
| ZKS1-1-25 | 0.05018 | 0.00187 | 0.23124 | 0.00847 | 0.03328 | 0.00043 | 203 | 61 | 211 | 7 | 211 | 3 |
| ZKS1-1-26 | 0.05703 | 0.00307 | 0.23037 | 0.01198 | 0.02914 | 0.00033 | 493 | 95 | 211 | 10 | 185 | 2 |
| ZKS1-1-27 | 0.05426 | 0.00207 | 0.21960 | 0.00813 | 0.02923 | 0.00030 | 382 | 65 | 202 | 7 | 186 | 2 |
| ZKS1-1-28 | 0.04888 | 0.00468 | 0.19270 | 0.01810 | 0.02859 | 0.00051 | 142 | 217 | 179 | 15 | 182 | 3 |
| ZKS1-1-29 | 0.05489 | 0.00208 | 0.22163 | 0.00774 | 0.02917 | 0.00047 | 408 | 49 | 203 | 6 | 185 | 3 |
| ZKS1-1-30 | 0.04610 | 0.00165 | 0.18563 | 0.00651 | 0.02908 | 0.00049 | 3 | 43 | 173 | 6 | 185 | 3 |
| ZKS1-1-31 | 0.05743 | 0.00270 | 0.26299 | 0.01136 | 0.03344 | 0.00045 | 508 | 71 | 237 | 9 | 212 | 3 |
| ZKS1-1-32 | 0.05301 | 0.00228 | 0.21250 | 0.00877 | 0.02907 | 0.00035 | 329 | 100 | 196 | 7 | 185 | 2 |
| ZKS1-1-33 | 0.05513 | 0.00189 | 0.22065 | 0.00717 | 0.02894 | 0.00046 | 417 | 45 | 202 | 6 | 184 | 3 |
| ZKS1-1-34 | 0.05261 | 0.00242 | 0.21226 | 0.00984 | 0.02910 | 0.00037 | 312 | 82 | 195 | 8 | 185 | 2 |
| ZKS1-1-35 | 0.04813 | 0.00505 | 0.19204 | 0.01986 | 0.02894 | 0.00051 | 105 | 231 | 178 | 17 | 184 | 3 |
| ZKS1-1-36 | 0.05466 | 0.00272 | 0.21861 | 0.01005 | 0.02901 | 0.00048 | 399 | 73 | 201 | 8 | 184 | 3 |
| 13ER14-1-03 | 0.05059 | 0.00112 | 0.18980 | 0.00407 | 0.02713 | 0.00020 | 222 | 36 | 176 | 3 | 173 | 1 |
| 13ER14-1-05 | 0.04948 | 0.00100 | 0.19692 | 0.00388 | 0.02886 | 0.00024 | 171 | 31 | 183 | 3 | 183 | 1 |
| 13ER14-1-06 | 0.05160 | 0.00103 | 0.21128 | 0.00462 | 0.02954 | 0.00025 | 268 | 35 | 195 | 4 | 188 | 2 |
| 13ER14-1-09 | 0.05201 | 0.00245 | 0.20854 | 0.00944 | 0.02935 | 0.00034 | 286 | 83 | 192 | 8 | 186 | 2 |
| 13ER14-1-10 | 0.04847 | 0.00186 | 0.18846 | 0.00688 | 0.02831 | 0.00028 | 122 | 67 | 175 | 6 | 180 | 2 |
| 13ER14-1-11 | 0.05365 | 0.00090 | 0.21352 | 0.00388 | 0.02866 | 0.00020 | 356 | 28 | 197 | 3 | 182 | 1 |
| 13ER14-1-12 | 0.04969 | 0.00182 | 0.20247 | 0.00728 | 0.02960 | 0.00037 | 181 | 60 | 187 | 6 | 188 | 2 |
| 13ER14-1-13 | 0.05081 | 0.00158 | 0.20670 | 0.00652 | 0.02943 | 0.00030 | 232 | 54 | 191 | 5 | 187 | 2 |
| 13ER14-1-16 | 0.05298 | 0.00284 | 0.21244 | 0.01125 | 0.02908 | 0.00042 | 328 | 94 | 196 | 9 | 185 | 3 |
| 13ER14-1-17 | 0.05302 | 0.00123 | 0.21295 | 0.00445 | 0.02913 | 0.00024 | 330 | 32 | 196 | 4 | 185 | 2 |
| 13ER14-1-18 | 0.04991 | 0.00096 | 0.20423 | 0.00384 | 0.02954 | 0.00017 | 191 | 33 | 189 | 3 | 188 | 1 |
| ER6-1-01 | 0.04965 | 0.00363 | 0.19143 | 0.01350 | 0.02797 | 0.00054 | 178 | 167 | 178 | 12 | 178 | 3 |
| ER6-1-02 | 0.05038 | 0.00542 | 0.19950 | 0.02095 | 0.02872 | 0.00068 | 212 | 242 | 185 | 18 | 183 | 4 |
| ER6-1-03 | 0.05552 | 0.00363 | 0.22131 | 0.01384 | 0.02891 | 0.00055 | 433 | 150 | 203 | 12 | 184 | 3 |
| ER6-1-04 | 0.06098 | 0.00210 | 0.24990 | 0.00820 | 0.02975 | 0.00034 | 639 | 51 | 226 | 7 | 189 | 2 |
| ER6-1-05 | 0.05281 | 0.00530 | 0.20298 | 0.02017 | 0.02788 | 0.00039 | 321 | 229 | 188 | 17 | 177 | 2 |
| ER6-1-06 | 0.05533 | 0.00291 | 0.24524 | 0.01265 | 0.03215 | 0.00033 | 426 | 120 | 223 | 10 | 204 | 2 |
| ER6-1-07 | 0.05692 | 0.00377 | 0.22520 | 0.01441 | 0.02870 | 0.00050 | 488 | 151 | 206 | 12 | 182 | 3 |
| ER6-1-08 | 0.05019 | 0.00302 | 0.19900 | 0.01174 | 0.02875 | 0.00033 | 204 | 138 | 184 | 10 | 183 | 2 |
| ER6-1-09 | 0.05102 | 0.00120 | 0.23015 | 0.00510 | 0.03272 | 0.00025 | 242 | 55 | 210 | 4 | 208 | 2 |
| ER6-1-10 | 0.05843 | 0.00304 | 0.24444 | 0.01242 | 0.03034 | 0.00034 | 546 | 117 | 222 | 10 | 193 | 2 |
| ER6-1-11 | 0.05610 | 0.00414 | 0.23205 | 0.01685 | 0.03000 | 0.00041 | 456 | 169 | 212 | 14 | 191 | 3 |
| ER6-1-12 | 0.05646 | 0.00415 | 0.25079 | 0.01797 | 0.03221 | 0.00052 | 471 | 168 | 227 | 15 | 204 | 3 |
| ER6-1-13 | 0.05511 | 0.00172 | 0.21977 | 0.00587 | 0.02904 | 0.00048 | 417 | 31 | 202 | 5 | 185 | 3 |
| ER6-1-14 | 0.06093 | 0.00274 | 0.25828 | 0.01338 | 0.03044 | 0.00034 | 637 | 93 | 233 | 11 | 193 | 2 |
| 13ER21-1-01 | 0.05015 | 0.00072 | 0.21332 | 0.00313 | 0.03070 | 0.00019 | 202 | 22 | 196 | 3 | 195 | 1 |
| 13ER21-1-02 | 0.05221 | 0.00137 | 0.21718 | 0.00595 | 0.03004 | 0.00031 | 295 | 43 | 200 | 5 | 191 | 2 |
| 13ER21-1-03 | 0.04832 | 0.00141 | 0.20589 | 0.00631 | 0.03073 | 0.00032 | 115 | 52 | 190 | 5 | 195 | 2 |
| 13ER21-1-04 | 0.04882 | 0.00083 | 0.20513 | 0.00346 | 0.03031 | 0.00019 | 139 | 28 | 189 | 3 | 193 | 1 |
| 13ER21-1-05 | 0.04955 | 0.00085 | 0.21392 | 0.00400 | 0.03113 | 0.00031 | 174 | 25 | 197 | 3 | 198 | 2 |
| 13ER21-1-06 | 0.04926 | 0.00107 | 0.21060 | 0.00469 | 0.03084 | 0.00028 | 160 | 35 | 194 | 4 | 196 | 2 |
| 13ER21-1-07 | 0.04865 | 0.00098 | 0.20727 | 0.00419 | 0.03077 | 0.00031 | 131 | 29 | 191 | 4 | 195 | 2 |
| 13ER21-1-08 | 0.04903 | 0.00083 | 0.20634 | 0.00352 | 0.03031 | 0.00019 | 149 | 28 | 190 | 3 | 193 | 1 |
| 13ER21-1-09 | 0.05012 | 0.00091 | 0.21669 | 0.00423 | 0.03113 | 0.00027 | 201 | 29 | 199 | 4 | 198 | 2 |
| 13ER21-1-10 | 0.05023 | 0.00078 | 0.21199 | 0.00344 | 0.03049 | 0.00027 | 205 | 21 | 195 | 3 | 194 | 2 |
| 13ER21-1-11 | 0.05025 | 0.00102 | 0.21600 | 0.00433 | 0.03105 | 0.00025 | 207 | 32 | 199 | 4 | 197 | 2 |
| 13ER21-1-12 | 0.05023 | 0.00136 | 0.21503 | 0.00606 | 0.03102 | 0.00052 | 206 | 36 | 198 | 5 | 197 | 3 |
| 13ER21-1-13 | 0.05085 | 0.00162 | 0.21663 | 0.00684 | 0.03103 | 0.00039 | 234 | 50 | 199 | 6 | 197 | 2 |
| 13ER21-1-14 | 0.05017 | 0.00133 | 0.21487 | 0.00557 | 0.03106 | 0.00031 | 203 | 42 | 198 | 5 | 197 | 2 |
| 13ER21-1-15 | 0.05230 | 0.00142 | 0.21997 | 0.00590 | 0.03039 | 0.00026 | 299 | 45 | 202 | 5 | 193 | 2 |
| 13ER21-1-16 | 0.05008 | 0.00093 | 0.21294 | 0.00404 | 0.03064 | 0.00021 | 199 | 31 | 196 | 3 | 195 | 1 |
| 13ER21-1-17 | 0.05070 | 0.00186 | 0.21396 | 0.00764 | 0.03061 | 0.00027 | 227 | 87 | 197 | 6 | 194 | 2 |
| 13ER6-1-01 | 0.04862 | 0.00185 | 0.20400 | 0.00729 | 0.03064 | 0.00033 | 129 | 63 | 189 | 6 | 195 | 2 |
| 13ER6-1-02 | 0.05234 | 0.00123 | 0.22456 | 0.00502 | 0.03104 | 0.00022 | 300 | 38 | 206 | 4 | 197 | 1 |
| 13ER6-1-03 | 0.05121 | 0.00142 | 0.21884 | 0.00588 | 0.03103 | 0.00029 | 250 | 44 | 201 | 5 | 197 | 2 |
| 13ER6-1-04 | 0.04944 | 0.00103 | 0.21300 | 0.00430 | 0.03112 | 0.00022 | 169 | 34 | 196 | 4 | 198 | 1 |
| 13ER6-1-05 | 0.04843 | 0.00121 | 0.20860 | 0.00525 | 0.03112 | 0.00027 | 120 | 43 | 192 | 4 | 198 | 2 |
| 13ER6-1-06 | 0.05198 | 0.00214 | 0.21844 | 0.00897 | 0.03071 | 0.00037 | 284 | 72 | 201 | 7 | 195 | 2 |
| 13ER6-1-07 | 0.05974 | 0.00405 | 0.25080 | 0.01507 | 0.03074 | 0.00053 | 594 | 101 | 227 | 12 | 195 | 3 |
| 13ER6-1-08 | 0.05324 | 0.00173 | 0.22544 | 0.00741 | 0.03055 | 0.00031 | 339 | 56 | 206 | 6 | 194 | 2 |
| 13ER6-1-09 | 0.05035 | 0.00137 | 0.21447 | 0.00576 | 0.03074 | 0.00023 | 211 | 48 | 197 | 5 | 195 | 1 |
| 13ER6-1-10 | 0.05090 | 0.00088 | 0.21693 | 0.00359 | 0.03075 | 0.00019 | 236 | 26 | 199 | 3 | 195 | 1 |
| 13ER6-1-11 | 0.05284 | 0.00169 | 0.22732 | 0.00715 | 0.03113 | 0.00030 | 322 | 54 | 208 | 6 | 198 | 2 |
| 13ER6-1-12 | 0.05203 | 0.00162 | 0.22207 | 0.00695 | 0.03085 | 0.00033 | 287 | 52 | 204 | 6 | 196 | 2 |
| 13ER6-1-13 | 0.05402 | 0.00191 | 0.22955 | 0.00830 | 0.03074 | 0.00042 | 372 | 57 | 210 | 7 | 195 | 3 |
| 13ER6-1-14 | 0.05372 | 0.00223 | 0.22724 | 0.00928 | 0.03079 | 0.00038 | 359 | 70 | 208 | 8 | 195 | 2 |
| 13ER6-1-15 | 0.05385 | 0.00225 | 0.22961 | 0.01012 | 0.03065 | 0.00029 | 365 | 82 | 210 | 8 | 195 | 2 |
| 13ER6-1-16 | 0.05492 | 0.00207 | 0.23367 | 0.00877 | 0.03089 | 0.00032 | 409 | 66 | 213 | 7 | 196 | 2 |
| 13TH1-1-01 | 0.05026 | 0.00104 | 0.21451 | 0.00444 | 0.03097 | 0.00006 | 207 | 45 | 197 | 4 | 196.6 | 0.4 |
| 13TH1-1-02 | 0.05043 | 0.00107 | 0.21542 | 0.00460 | 0.03097 | 0.00007 | 215 | 46 | 198 | 4 | 196.6 | 0.4 |
| 13TH1-1-03 | 0.05025 | 0.00087 | 0.21475 | 0.00372 | 0.03100 | 0.00006 | 206 | 37 | 198 | 3 | 196.8 | 0.4 |
| 13TH1-1-04 | 0.05104 | 0.00138 | 0.21780 | 0.00581 | 0.03096 | 0.00007 | 243 | 58 | 200 | 5 | 196.6 | 0.5 |
| 13TH1-1-05 | 0.05107 | 0.00149 | 0.21859 | 0.00670 | 0.03093 | 0.00008 | 244 | 67 | 201 | 6 | 196.4 | 0.5 |
| 13TH1-1-06 | 0.05024 | 0.00158 | 0.21444 | 0.00681 | 0.03094 | 0.00008 | 206 | 70 | 197 | 6 | 196.4 | 0.5 |
| 13TH1-1-07 | 0.05032 | 0.00202 | 0.21525 | 0.00906 | 0.03093 | 0.00009 | 210 | 94 | 198 | 8 | 196.4 | 0.5 |
| 13TH1-1-08 | 0.05013 | 0.00120 | 0.21353 | 0.00516 | 0.03089 | 0.00006 | 201 | 53 | 197 | 4 | 196.1 | 0.4 |
| 13TH1-1-09 | 0.05010 | 0.00203 | 0.21342 | 0.00864 | 0.03090 | 0.00009 | 200 | 90 | 196 | 7 | 196.2 | 0.6 |
| 13TH1-1-10 | 0.05028 | 0.00127 | 0.21401 | 0.00550 | 0.03086 | 0.00008 | 208 | 56 | 197 | 5 | 196 | 0.5 |
| 13TH1-1-11 | 0.05045 | 0.00138 | 0.21521 | 0.00603 | 0.03090 | 0.00006 | 216 | 62 | 198 | 5 | 196.2 | 0.4 |
| 13TH1-1-12 | 0.05008 | 0.00185 | 0.21363 | 0.00781 | 0.03098 | 0.00008 | 199 | 81 | 197 | 7 | 196.7 | 0.5 |
| 13TH1-1-13 | 0.05005 | 0.00097 | 0.21312 | 0.00419 | 0.03088 | 0.00007 | 197 | 42 | 196 | 4 | 196.1 | 0.4 |
| 13TH1-1-14 | 0.05042 | 0.00108 | 0.21448 | 0.00459 | 0.03087 | 0.00007 | 215 | 46 | 197 | 4 | 196 | 0.4 |
| 13TH1-1-15 | 0.05072 | 0.00142 | 0.21650 | 0.00616 | 0.03094 | 0.00007 | 228 | 63 | 199 | 5 | 196.4 | 0.4 |
| 13TH1-1-16 | 0.05036 | 0.00120 | 0.21440 | 0.00512 | 0.03088 | 0.00007 | 212 | 52 | 197 | 4 | 196.1 | 0.4 |
| 13TH1-1-17 | 0.05056 | 0.00129 | 0.21545 | 0.00558 | 0.03090 | 0.00007 | 221 | 56 | 198 | 5 | 196.2 | 0.5 |
| 13TH1-1-18 | 0.05049 | 0.00130 | 0.21588 | 0.00575 | 0.03095 | 0.00008 | 218 | 57 | 198 | 5 | 196.5 | 0.5 |
| 13TH1-1-19 | 0.05003 | 0.00174 | 0.21332 | 0.00770 | 0.03088 | 0.00009 | 197 | 80 | 196 | 6 | 196.1 | 0.6 |
| 13TH1-1-20 | 0.05037 | 0.00167 | 0.21464 | 0.00718 | 0.03092 | 0.00008 | 212 | 74 | 197 | 6 | 196.3 | 0.5 |
| 13TH1-1-21 | 0.05070 | 0.00260 | 0.21599 | 0.01106 | 0.03089 | 0.00008 | 227 | 114 | 199 | 9 | 196.1 | 0.5 |
| 13TH1-1-22 | 0.05078 | 0.00111 | 0.21632 | 0.00466 | 0.03092 | 0.00007 | 231 | 46 | 199 | 4 | 196.3 | 0.5 |
| 12ER27-1-01 | 0.05216 | 0.00492 | 0.25446 | 0.02470 | 0.03537 | 0.00102 | 293 | 166 | 230 | 20 | 224 | 6 |
| 12ER27-1-02 | 0.05580 | 0.00301 | 0.23316 | 0.01217 | 0.03061 | 0.00045 | 444 | 90 | 213 | 10 | 194 | 3 |
| 12ER27-1-03 | 0.05035 | 0.00316 | 0.21015 | 0.01258 | 0.03031 | 0.00048 | 211 | 108 | 194 | 11 | 192 | 3 |
| 12ER27-1-04 | 0.05719 | 0.00578 | 0.22796 | 0.02161 | 0.02970 | 0.00084 | 499 | 160 | 209 | 18 | 189 | 5 |
| 12ER27-1-05 | 0.05880 | 0.00441 | 0.24491 | 0.01826 | 0.03089 | 0.00057 | 560 | 131 | 222 | 15 | 196 | 4 |
| 12ER27-1-06 | 0.05054 | 0.00270 | 0.21316 | 0.01145 | 0.03036 | 0.00044 | 220 | 98 | 196 | 10 | 193 | 3 |
| 12ER27-1-07 | 0.05582 | 0.00344 | 0.24720 | 0.01563 | 0.03213 | 0.00059 | 445 | 108 | 224 | 13 | 204 | 4 |
| 12ER27-1-08 | 0.04896 | 0.00208 | 0.20720 | 0.00842 | 0.03074 | 0.00038 | 146 | 71 | 191 | 7 | 195 | 2 |
| 12ER27-1-09 | 0.05186 | 0.00332 | 0.22318 | 0.01280 | 0.03213 | 0.00058 | 279 | 98 | 205 | 11 | 204 | 4 |
| 12ER27-1-10 | 0.05248 | 0.00341 | 0.21940 | 0.01358 | 0.03045 | 0.00068 | 306 | 100 | 201 | 11 | 193 | 4 |
| 12ER27-1-11 | 0.05743 | 0.00302 | 0.23764 | 0.01174 | 0.03017 | 0.00047 | 508 | 81 | 216 | 10 | 192 | 3 |
| 12ER27-1-12 | 0.05195 | 0.00302 | 0.22047 | 0.01190 | 0.03098 | 0.00044 | 283 | 97 | 202 | 10 | 197 | 3 |
| 12ER27-1-13 | 0.05428 | 0.00316 | 0.22834 | 0.01402 | 0.03002 | 0.00048 | 383 | 109 | 209 | 12 | 191 | 3 |
| 12ER27-1-14 | 0.05088 | 0.00269 | 0.21262 | 0.01106 | 0.03024 | 0.00044 | 235 | 93 | 196 | 9 | 192 | 3 |
| 12ER27-1-15 | 0.05852 | 0.00280 | 0.25006 | 0.01175 | 0.03104 | 0.00048 | 549 | 76 | 227 | 10 | 197 | 3 |
| 12ER27-1-16 | 0.05485 | 0.00321 | 0.23110 | 0.01313 | 0.03097 | 0.00052 | 406 | 97 | 211 | 11 | 197 | 3 |
| 12ER27-1-17 | 0.05688 | 0.00327 | 0.24905 | 0.01413 | 0.03188 | 0.00057 | 487 | 94 | 226 | 11 | 202 | 4 |
| 12ER27-1-18 | 0.05636 | 0.00376 | 0.22298 | 0.01326 | 0.03009 | 0.00056 | 467 | 99 | 204 | 11 | 191 | 3 |
| 12ER27-1-19 | 0.05339 | 0.00377 | 0.22016 | 0.01492 | 0.03104 | 0.00087 | 346 | 103 | 202 | 12 | 197 | 5 |
| 12ER27-1-20 | 0.04976 | 0.00209 | 0.20731 | 0.00828 | 0.03018 | 0.00031 | 184 | 74 | 191 | 7 | 192 | 2 |
| 12ER27-1-21 | 0.05450 | 0.00337 | 0.22663 | 0.01263 | 0.03109 | 0.00048 | 392 | 98 | 207 | 10 | 197 | 3 |
| 12ER27-1-22 | 0.04901 | 0.00303 | 0.21538 | 0.01282 | 0.03198 | 0.00045 | 148 | 108 | 198 | 11 | 203 | 3 |
| 12ER27-1-23 | 0.05174 | 0.00314 | 0.22357 | 0.01282 | 0.03202 | 0.00062 | 274 | 96 | 205 | 11 | 203 | 4 |
| 12ER27-1-24 | 0.05166 | 0.00237 | 0.22400 | 0.01001 | 0.03123 | 0.00035 | 271 | 82 | 205 | 8 | 198 | 2 |
| 12ER27-1-25 | 0.05711 | 0.00305 | 0.25595 | 0.01361 | 0.03225 | 0.00043 | 496 | 94 | 231 | 11 | 205 | 3 |
| 12ER27-1-26 | 0.05101 | 0.00316 | 0.22263 | 0.01390 | 0.03207 | 0.00048 | 241 | 116 | 204 | 12 | 203 | 3 |
| 12ER27-1-27 | 0.05222 | 0.00381 | 0.22626 | 0.01526 | 0.03239 | 0.00059 | 295 | 121 | 207 | 13 | 205 | 4 |
| 12ER27-1-28 | 0.05728 | 0.00613 | 0.27819 | 0.02635 | 0.03636 | 0.00161 | 502 | 132 | 249 | 21 | 230 | 10 |
| 12ER27-1-29 | 0.06119 | 0.00539 | 0.25964 | 0.02210 | 0.03152 | 0.00079 | 646 | 140 | 234 | 18 | 200 | 5 |
| 11ER10-1-01 | 0.05137 | 0.00188 | 0.23152 | 0.00830 | 0.03242 | 0.00041 | 258 | 59 | 211 | 7 | 206 | 3 |
| 11ER10-1-02 | 0.05045 | 0.00221 | 0.25911 | 0.01089 | 0.03700 | 0.00046 | 216 | 74 | 234 | 9 | 234 | 3 |
| 11ER10-1-03 | 0.05278 | 0.00436 | 0.27414 | 0.02235 | 0.03739 | 0.00075 | 319 | 149 | 246 | 18 | 237 | 5 |
| 11ER10-1-04 | 0.05148 | 0.00239 | 0.23027 | 0.01041 | 0.03228 | 0.00047 | 262 | 77 | 210 | 9 | 205 | 3 |
| 11ER10-1-05 | 0.05187 | 0.00324 | 0.22516 | 0.01328 | 0.03155 | 0.00053 | 279 | 104 | 206 | 11 | 200 | 3 |
| 11ER10-1-06 | 0.04931 | 0.00239 | 0.21830 | 0.01033 | 0.03205 | 0.00050 | 163 | 81 | 200 | 9 | 203 | 3 |
| 11ER10-1-07 | 0.05220 | 0.00176 | 0.23078 | 0.00747 | 0.03196 | 0.00042 | 294 | 50 | 211 | 6 | 203 | 3 |
| 11ER10-1-08 | 0.05016 | 0.00260 | 0.22055 | 0.01132 | 0.03183 | 0.00057 | 203 | 86 | 202 | 9 | 202 | 4 |
| 11ER10-1-09 | 0.05457 | 0.00181 | 0.23840 | 0.00755 | 0.03148 | 0.00035 | 395 | 51 | 217 | 6 | 200 | 2 |
| 11ER10-1-10 | 0.04984 | 0.00278 | 0.23443 | 0.01297 | 0.03386 | 0.00053 | 187 | 99 | 214 | 11 | 215 | 3 |
| 11ER10-1-11 | 0.04908 | 0.00224 | 0.26868 | 0.01219 | 0.03969 | 0.00060 | 151 | 77 | 242 | 10 | 251 | 4 |
| 11ER10-1-12 | 0.05076 | 0.00256 | 0.23577 | 0.01145 | 0.03389 | 0.00056 | 230 | 82 | 215 | 9 | 215 | 3 |
| 11ER10-1-13 | 0.05032 | 0.00229 | 0.23671 | 0.01054 | 0.03397 | 0.00049 | 210 | 76 | 216 | 9 | 215 | 3 |
| 11ER10-1-14 | 0.05060 | 0.00245 | 0.22292 | 0.01115 | 0.03171 | 0.00049 | 223 | 87 | 204 | 9 | 201 | 3 |
| 11ER10-1-15 | 0.05113 | 0.00215 | 0.22728 | 0.00981 | 0.03197 | 0.00042 | 247 | 75 | 208 | 8 | 203 | 3 |
| 11ER10-1-16 | 0.05053 | 0.00245 | 0.22104 | 0.01060 | 0.03178 | 0.00069 | 219 | 71 | 203 | 9 | 202 | 4 |
| 11ER13-1-01 | 0.05157 | 0.00105 | 0.25740 | 0.00552 | 0.03612 | 0.00029 | 266 | 34 | 233 | 4 | 229 | 2 |
| 11ER13-1-02 | 0.05141 | 0.00182 | 0.29023 | 0.00999 | 0.04115 | 0.00046 | 259 | 58 | 259 | 8 | 260 | 3 |
| 11ER13-1-03 | 0.05556 | 0.00143 | 0.31366 | 0.00791 | 0.04090 | 0.00038 | 435 | 40 | 277 | 6 | 258 | 2 |
| 11ER13-1-04 | 0.05601 | 0.00158 | 0.29206 | 0.00836 | 0.03781 | 0.00048 | 453 | 41 | 260 | 7 | 239 | 3 |
| 11ER13-1-05 | 0.04959 | 0.00232 | 0.22007 | 0.01071 | 0.03199 | 0.00047 | 176 | 86 | 202 | 9 | 203 | 3 |
| 11ER13-1-06 | 0.05441 | 0.00164 | 0.29306 | 0.00904 | 0.03884 | 0.00045 | 388 | 48 | 261 | 7 | 246 | 3 |
| 11ER13-1-07 | 0.05461 | 0.00210 | 0.28802 | 0.01068 | 0.03832 | 0.00053 | 396 | 58 | 257 | 8 | 242 | 3 |
| 11ER13-1-08 | 0.05258 | 0.00177 | 0.27880 | 0.00950 | 0.03828 | 0.00041 | 311 | 58 | 250 | 8 | 242 | 3 |
| 11ER13-1-09 | 0.05317 | 0.00361 | 0.28315 | 0.01884 | 0.03862 | 0.00051 | 336 | 157 | 253 | 15 | 244 | 3 |
| 11ER13-1-10 | 0.05358 | 0.00181 | 0.28945 | 0.00932 | 0.03923 | 0.00045 | 353 | 52 | 258 | 7 | 248 | 3 |
| 11ER13-1-11 | 0.05121 | 0.00280 | 0.22543 | 0.01177 | 0.03193 | 0.00051 | 250 | 127 | 206 | 10 | 203 | 3 |
| 11ER13-1-12 | 0.05301 | 0.00188 | 0.28201 | 0.01008 | 0.03845 | 0.00047 | 329 | 59 | 252 | 8 | 243 | 3 |
| 11ER13-1-13 | 0.05081 | 0.00209 | 0.28805 | 0.01177 | 0.04094 | 0.00050 | 232 | 72 | 257 | 9 | 259 | 3 |
| 11ER13-1-14 | 0.05501 | 0.00321 | 0.28826 | 0.01632 | 0.03840 | 0.00065 | 413 | 96 | 257 | 13 | 243 | 4 |
| 11ER13-1-15 | 0.05504 | 0.00244 | 0.24355 | 0.01051 | 0.03207 | 0.00046 | 414 | 71 | 221 | 9 | 203 | 3 |
| 11ER13-1-16 | 0.05571 | 0.00233 | 0.30034 | 0.01268 | 0.03876 | 0.00048 | 441 | 72 | 267 | 10 | 245 | 3 |
| 11ER13-1-17 | 0.05324 | 0.00258 | 0.30638 | 0.01446 | 0.04186 | 0.00062 | 339 | 80 | 271 | 11 | 264 | 4 |
| 11ER13-1-18 | 0.05002 | 0.00358 | 0.26317 | 0.01763 | 0.03816 | 0.00097 | 196 | 164 | 237 | 14 | 241 | 6 |
| 11ER13-1-19 | 0.05315 | 0.00194 | 0.29843 | 0.01038 | 0.04076 | 0.00054 | 335 | 55 | 265 | 8 | 258 | 3 |
| 11ER13-1-20 | 0.05031 | 0.00464 | 0.26753 | 0.02413 | 0.03857 | 0.00072 | 209 | 210 | 241 | 19 | 244 | 4 |
| 12ER17-1-01 | 0.05245 | 0.00186 | 0.25525 | 0.00876 | 0.03516 | 0.00033 | 305 | 61 | 231 | 7 | 223 | 2 |
| 12ER17-1-02 | 0.04798 | 0.00209 | 0.21587 | 0.00931 | 0.03253 | 0.00035 | 98 | 77 | 198 | 8 | 206 | 2 |
| 12ER17-1-03 | 0.05108 | 0.00204 | 0.22754 | 0.00924 | 0.03210 | 0.00033 | 244 | 75 | 208 | 8 | 204 | 2 |
| 12ER17-1-04 | 0.05405 | 0.00247 | 0.25947 | 0.01118 | 0.03482 | 0.00053 | 373 | 105 | 234 | 9 | 221 | 3 |
| 12ER17-1-05 | 0.04748 | 0.00176 | 0.21480 | 0.00814 | 0.03256 | 0.00041 | 73 | 62 | 198 | 7 | 207 | 3 |
| 12ER17-1-06 | 0.05195 | 0.00220 | 0.23206 | 0.00972 | 0.03234 | 0.00035 | 283 | 76 | 212 | 8 | 205 | 2 |
| 12ER17-1-07 | 0.05166 | 0.00197 | 0.23439 | 0.00894 | 0.03275 | 0.00036 | 270 | 68 | 214 | 7 | 208 | 2 |
| 12ER17-1-08 | 0.05247 | 0.00169 | 0.23717 | 0.00754 | 0.03265 | 0.00032 | 306 | 54 | 216 | 6 | 207 | 2 |
| 12ER17-1-09 | 0.05223 | 0.00195 | 0.23023 | 0.00856 | 0.03171 | 0.00033 | 295 | 66 | 210 | 7 | 201 | 2 |
| 12ER17-1-10 | 0.05125 | 0.00176 | 0.24319 | 0.00817 | 0.03435 | 0.00043 | 252 | 54 | 221 | 7 | 218 | 3 |
| 12ER17-1-11 | 0.05793 | 0.00231 | 0.25524 | 0.00988 | 0.03196 | 0.00031 | 527 | 90 | 231 | 8 | 203 | 2 |
| 12ER17-1-12 | 0.05559 | 0.00222 | 0.24632 | 0.00971 | 0.03199 | 0.00035 | 436 | 68 | 224 | 8 | 203 | 2 |
| 12ER17-1-13 | 0.05287 | 0.00175 | 0.26060 | 0.00830 | 0.03547 | 0.00033 | 323 | 55 | 235 | 7 | 225 | 2 |
| 12ER17-1-14 | 0.05178 | 0.00151 | 0.25415 | 0.00729 | 0.03523 | 0.00032 | 276 | 49 | 230 | 6 | 223 | 2 |
| 12ER17-1-15 | 0.05256 | 0.00177 | 0.25782 | 0.00858 | 0.03521 | 0.00038 | 310 | 56 | 233 | 7 | 223 | 2 |
| 12ER17-1-16 | 0.05181 | 0.00150 | 0.23565 | 0.00664 | 0.03268 | 0.00032 | 277 | 47 | 215 | 5 | 207 | 2 |
| 12ER17-1-17 | 0.05168 | 0.00275 | 0.24599 | 0.01285 | 0.03452 | 0.00036 | 271 | 125 | 223 | 10 | 219 | 2 |
| 12ER17-1-18 | 0.05768 | 0.00279 | 0.28222 | 0.01292 | 0.03550 | 0.00038 | 518 | 82 | 252 | 10 | 225 | 2 |
| 12ER17-1-19 | 0.05313 | 0.00202 | 0.23884 | 0.00884 | 0.03240 | 0.00033 | 334 | 65 | 217 | 7 | 206 | 2 |
| 12ER17-1-20 | 0.05513 | 0.00208 | 0.24178 | 0.00881 | 0.03167 | 0.00029 | 418 | 65 | 220 | 7 | 201 | 2 |
| 12ER19-1-01 | 0.04967 | 0.00146 | 0.22316 | 0.00654 | 0.03224 | 0.00037 | 179 | 47 | 205 | 5 | 205 | 2 |
| 12ER19-1-02 | 0.05072 | 0.00169 | 0.24587 | 0.00782 | 0.03488 | 0.00039 | 228 | 53 | 223 | 6 | 221 | 2 |
| 12ER19-1-03 | 0.04851 | 0.00235 | 0.21938 | 0.00997 | 0.03262 | 0.00050 | 124 | 76 | 201 | 8 | 207 | 3 |
| 12ER19-1-04 | 0.04998 | 0.00189 | 0.22660 | 0.00832 | 0.03254 | 0.00047 | 194 | 59 | 207 | 7 | 206 | 3 |
| 12ER19-1-05 | 0.04844 | 0.00187 | 0.23828 | 0.00918 | 0.03511 | 0.00048 | 121 | 64 | 217 | 8 | 222 | 3 |
| 12ER19-1-06 | 0.04973 | 0.00186 | 0.22350 | 0.00807 | 0.03224 | 0.00039 | 183 | 62 | 205 | 7 | 205 | 2 |
| 12ER19-1-07 | 0.05114 | 0.00184 | 0.23344 | 0.00851 | 0.03266 | 0.00041 | 247 | 61 | 213 | 7 | 207 | 3 |
| 12ER19-1-08 | 0.04908 | 0.00240 | 0.21659 | 0.01027 | 0.03166 | 0.00038 | 152 | 87 | 199 | 9 | 201 | 2 |
| 12ER19-1-09 | 0.05265 | 0.00223 | 0.24070 | 0.01035 | 0.03294 | 0.00046 | 314 | 72 | 219 | 8 | 209 | 3 |
| 12ER19-1-10 | 0.05051 | 0.00212 | 0.34349 | 0.01505 | 0.04885 | 0.00080 | 218 | 71 | 300 | 11 | 307 | 5 |
| 12ER19-1-11 | 0.05036 | 0.00150 | 0.22631 | 0.00724 | 0.03226 | 0.00046 | 212 | 48 | 207 | 6 | 205 | 3 |
| 12ER19-1-12 | 0.04715 | 0.00169 | 0.21414 | 0.00792 | 0.03278 | 0.00042 | 57 | 58 | 197 | 7 | 208 | 3 |
| 12ER19-1-13 | 0.05234 | 0.00446 | 0.22807 | 0.01832 | 0.03194 | 0.00082 | 300 | 136 | 209 | 15 | 203 | 5 |
| 12ER19-1-14 | 0.04884 | 0.00147 | 0.21975 | 0.00661 | 0.03241 | 0.00037 | 140 | 49 | 202 | 6 | 206 | 2 |
| 12ER19-1-15 | 0.05102 | 0.00251 | 0.24328 | 0.01179 | 0.03480 | 0.00046 | 242 | 87 | 221 | 10 | 221 | 3 |
| 12ER19-1-16 | 0.05632 | 0.00289 | 0.27416 | 0.01423 | 0.03550 | 0.00062 | 465 | 84 | 246 | 11 | 225 | 4 |
| 12ER19-1-17 | 0.04928 | 0.00199 | 0.22072 | 0.00867 | 0.03245 | 0.00036 | 161 | 71 | 203 | 7 | 206 | 2 |
| 12ER19-1-18 | 0.04658 | 0.00219 | 0.22808 | 0.01066 | 0.03535 | 0.00047 | 28 | 75 | 209 | 9 | 224 | 3 |
| 12ER19-1-19 | 0.04909 | 0.00236 | 0.23781 | 0.01109 | 0.03524 | 0.00046 | 152 | 84 | 217 | 9 | 223 | 3 |
| 12ER19-1-20 | 0.04882 | 0.00151 | 0.21827 | 0.00694 | 0.03213 | 0.00043 | 139 | 50 | 200 | 6 | 204 | 3 |
| 12ER19-1-21 | 0.05035 | 0.00158 | 0.22505 | 0.00685 | 0.03219 | 0.00030 | 211 | 53 | 206 | 6 | 204 | 2 |
| 12ER19-1-22 | 0.05068 | 0.00173 | 0.24765 | 0.00826 | 0.03530 | 0.00036 | 226 | 58 | 225 | 7 | 224 | 2 |
| 12ER19-1-23 | 0.05404 | 0.00402 | 0.25956 | 0.01877 | 0.03483 | 0.00061 | 373 | 171 | 234 | 15 | 221 | 4 |
| 12ER19-1-24 | 0.04825 | 0.00364 | 0.21382 | 0.01846 | 0.03193 | 0.00083 | 111 | 143 | 197 | 15 | 203 | 5 |
| 12ER19-1-25 | 0.05210 | 0.00232 | 0.23846 | 0.01116 | 0.03283 | 0.00048 | 290 | 80 | 217 | 9 | 208 | 3 |
| 12ER19-1-26 | 0.05411 | 0.00200 | 0.30929 | 0.01120 | 0.04130 | 0.00054 | 375 | 58 | 274 | 9 | 261 | 3 |
| 12ER19-1-27 | 0.05140 | 0.00194 | 0.24742 | 0.00900 | 0.03484 | 0.00054 | 259 | 55 | 224 | 7 | 221 | 3 |
| 12ER19-1-28 | 0.05037 | 0.00152 | 0.22802 | 0.00676 | 0.03250 | 0.00041 | 212 | 45 | 209 | 6 | 206 | 3 |
| 12ER19-1-29 | 0.05072 | 0.00210 | 0.22891 | 0.00954 | 0.03233 | 0.00047 | 228 | 69 | 209 | 8 | 205 | 3 |
| 14ER18-1-01 | 0.05202 | 0.00265 | 0.23323 | 0.01199 | 0.03253 | 0.00042 | 286 | 94 | 213 | 10 | 206 | 3 |
| 14ER18-1-02 | 0.05205 | 0.00237 | 0.22997 | 0.01024 | 0.03214 | 0.00039 | 288 | 80 | 210 | 8 | 204 | 2 |
| 14ER18-1-03 | 0.05184 | 0.00179 | 0.22872 | 0.00786 | 0.03203 | 0.00035 | 278 | 59 | 209 | 6 | 203 | 2 |
| 14ER18-1-04 | 0.05261 | 0.00280 | 0.23263 | 0.01206 | 0.03207 | 0.00041 | 312 | 95 | 212 | 10 | 204 | 3 |
| 14ER18-1-05 | 0.05272 | 0.00195 | 0.23589 | 0.00834 | 0.03250 | 0.00037 | 317 | 60 | 215 | 7 | 206 | 2 |
| 14ER18-1-06 | 0.05144 | 0.00233 | 0.23131 | 0.01023 | 0.03246 | 0.00036 | 260 | 81 | 211 | 8 | 206 | 2 |
| 14ER18-1-07 | 0.05194 | 0.00240 | 0.22939 | 0.01011 | 0.03214 | 0.00037 | 283 | 80 | 210 | 8 | 204 | 2 |
| 14ER18-1-08 | 0.05191 | 0.00139 | 0.23458 | 0.00652 | 0.03249 | 0.00031 | 281 | 46 | 214 | 5 | 206 | 2 |
| 14ER18-1-09 | 0.05170 | 0.00201 | 0.22870 | 0.00857 | 0.03219 | 0.00035 | 272 | 66 | 209 | 7 | 204 | 2 |
| 14ER18-1-10 | 0.05146 | 0.00253 | 0.22849 | 0.01105 | 0.03219 | 0.00042 | 261 | 87 | 209 | 9 | 204 | 3 |
| 14ER18-1-11 | 0.05118 | 0.00311 | 0.23066 | 0.01408 | 0.03249 | 0.00043 | 249 | 116 | 211 | 12 | 206 | 3 |
| 14ER18-1-12 | 0.05376 | 0.00247 | 0.23998 | 0.01084 | 0.03247 | 0.00041 | 361 | 79 | 218 | 9 | 206 | 3 |
| 14ER18-1-13 | 0.05340 | 0.00266 | 0.23922 | 0.01144 | 0.03244 | 0.00041 | 346 | 86 | 218 | 9 | 206 | 3 |
| 14ER18-1-14 | 0.05301 | 0.00142 | 0.23536 | 0.00606 | 0.03222 | 0.00041 | 329 | 35 | 215 | 5 | 204 | 3 |
| 14ER18-1-15 | 0.05142 | 0.00223 | 0.22745 | 0.00931 | 0.03229 | 0.00036 | 260 | 73 | 208 | 8 | 205 | 2 |
| 14ER18-1-16 | 0.05187 | 0.00289 | 0.23104 | 0.01239 | 0.03230 | 0.00040 | 280 | 101 | 211 | 10 | 205 | 2 |
| 14ER18-1-17 | 0.05270 | 0.00481 | 0.23264 | 0.01884 | 0.03243 | 0.00046 | 316 | 158 | 212 | 16 | 206 | 3 |
| 14ER18-1-18 | 0.05518 | 0.00144 | 0.24860 | 0.00641 | 0.03242 | 0.00031 | 419 | 40 | 225 | 5 | 206 | 2 |
| 14ER18-1-19 | 0.05246 | 0.00337 | 0.22989 | 0.01370 | 0.03232 | 0.00060 | 306 | 102 | 210 | 11 | 205 | 4 |
| 14ER18-1-20 | 0.05082 | 0.00280 | 0.22676 | 0.01211 | 0.03238 | 0.00040 | 233 | 101 | 208 | 10 | 205 | 2 |
| 18ER12-1-01 | 0.06325 | 0.00153 | 0.95772 | 0.02985 | 0.10932 | 0.00237 | 717 | 33 | 682 | 15 | 669 | 14 |
| 18ER12-1-02 | 0.05145 | 0.00195 | 0.22894 | 0.00853 | 0.03222 | 0.00030 | 261 | 68 | 209 | 7 | 204 | 2 |
| 18ER12-1-04 | 0.06641 | 0.00116 | 1.20207 | 0.02120 | 0.13052 | 0.00093 | 819 | 25 | 802 | 10 | 791 | 5 |
| 18ER12-1-05 | 0.06622 | 0.00145 | 0.88073 | 0.01954 | 0.09597 | 0.00077 | 813 | 33 | 641 | 11 | 591 | 5 |
| 18ER12-1-06 | 0.06113 | 0.00195 | 0.63936 | 0.02899 | 0.07434 | 0.00210 | 644 | 51 | 502 | 18 | 462 | 13 |
| 18ER12-1-07 | 0.05100 | 0.00306 | 0.23222 | 0.01374 | 0.03292 | 0.00059 | 241 | 103 | 212 | 11 | 209 | 4 |
| 18ER12-1-08 | 0.05519 | 0.00358 | 0.45026 | 0.02602 | 0.05917 | 0.00173 | 420 | 149 | 377 | 18 | 371 | 11 |
| 18ER12-1-09 | 0.05549 | 0.00184 | 0.32501 | 0.01001 | 0.04248 | 0.00051 | 432 | 76 | 286 | 8 | 268 | 3 |
| 18ER12-1-10 | 0.05083 | 0.00240 | 0.23121 | 0.01039 | 0.03290 | 0.00038 | 233 | 82 | 211 | 9 | 209 | 2 |
| 18ER12-1-11 | 0.05671 | 0.00129 | 0.42074 | 0.01410 | 0.05328 | 0.00125 | 480 | 36 | 357 | 10 | 335 | 8 |
| 18ER12-1-12 | 0.05431 | 0.00164 | 0.24678 | 0.00811 | 0.03280 | 0.00049 | 384 | 47 | 224 | 7 | 208 | 3 |
| 18ER12-1-13 | 0.05469 | 0.00375 | 0.45263 | 0.02876 | 0.06003 | 0.00154 | 399 | 158 | 379 | 20 | 376 | 9 |
| 18ER12-1-14 | 0.06394 | 0.00130 | 1.15632 | 0.02638 | 0.13022 | 0.00133 | 740 | 31 | 780 | 12 | 789 | 8 |
| 18ER12-1-15 | 0.05133 | 0.00131 | 0.23079 | 0.00588 | 0.03252 | 0.00030 | 256 | 41 | 211 | 5 | 206 | 2 |
| 18ER12-1-16 | 0.06410 | 0.00133 | 0.92076 | 0.03385 | 0.10247 | 0.00299 | 745 | 36 | 663 | 18 | 629 | 17 |
| 18ER12-1-17 | 0.05693 | 0.00136 | 0.25409 | 0.00634 | 0.03220 | 0.00032 | 489 | 38 | 230 | 5 | 204 | 2 |
| 18ER12-1-18 | 0.05334 | 0.00147 | 0.25451 | 0.00683 | 0.03449 | 0.00031 | 343 | 44 | 230 | 6 | 219 | 2 |
| 18ER12-1-19 | 0.05765 | 0.00185 | 0.26598 | 0.00903 | 0.03330 | 0.00043 | 516 | 52 | 239 | 7 | 211 | 3 |
| 18ER12-1-20 | 0.06566 | 0.00140 | 1.18810 | 0.02470 | 0.13049 | 0.00089 | 796 | 32 | 795 | 11 | 791 | 5 |
| 18ER12-1-22 | 0.05162 | 0.00213 | 0.23057 | 0.00929 | 0.03239 | 0.00029 | 269 | 97 | 211 | 8 | 206 | 2 |
| 18ER12-1-23 | 0.05294 | 0.00187 | 0.23602 | 0.00852 | 0.03227 | 0.00037 | 326 | 61 | 215 | 7 | 205 | 2 |
| 18ER12-1-24 | 0.05138 | 0.00131 | 0.22859 | 0.00579 | 0.03214 | 0.00030 | 258 | 41 | 209 | 5 | 204 | 2 |
| 18ER12-1-25 | 0.06416 | 0.00190 | 0.91161 | 0.03531 | 0.10203 | 0.00281 | 747 | 40 | 658 | 19 | 626 | 16 |
| 12ER34-1-01 | 0.05265 | 0.00252 | 0.23075 | 0.01103 | 0.03153 | 0.00037 | 314 | 88 | 211 | 9 | 200 | 2 |
| 12ER34-1-02 | 0.04692 | 0.00248 | 0.21061 | 0.01077 | 0.03253 | 0.00045 | 45 | 85 | 194 | 9 | 206 | 3 |
| 12ER34-1-03 | 0.05416 | 0.00265 | 0.23912 | 0.01121 | 0.03214 | 0.00044 | 378 | 81 | 218 | 9 | 204 | 3 |
| 12ER34-1-04 | 0.04971 | 0.00264 | 0.22374 | 0.01148 | 0.03248 | 0.00041 | 181 | 95 | 205 | 10 | 206 | 3 |
| 12ER34-1-05 | 0.05012 | 0.00263 | 0.22656 | 0.01226 | 0.03249 | 0.00051 | 201 | 96 | 207 | 10 | 206 | 3 |
| 12ER34-1-06 | 0.05009 | 0.00251 | 0.22796 | 0.01156 | 0.03252 | 0.00044 | 199 | 92 | 209 | 10 | 206 | 3 |
| 12ER34-1-07 | 0.05694 | 0.00294 | 0.25285 | 0.01219 | 0.03248 | 0.00048 | 489 | 80 | 229 | 10 | 206 | 3 |
| 12ER34-1-08 | 0.05574 | 0.00278 | 0.26374 | 0.01282 | 0.03438 | 0.00048 | 442 | 83 | 238 | 10 | 218 | 3 |
| 12ER34-1-09 | 0.06122 | 0.00333 | 0.28835 | 0.01511 | 0.03432 | 0.00050 | 647 | 88 | 257 | 12 | 218 | 3 |
| 12ER34-1-10 | 0.05292 | 0.00353 | 0.25291 | 0.01736 | 0.03443 | 0.00054 | 326 | 128 | 229 | 14 | 218 | 3 |
| 12ER34-1-11 | 0.05058 | 0.00324 | 0.22643 | 0.01487 | 0.03193 | 0.00051 | 222 | 121 | 207 | 12 | 203 | 3 |
| 12ER34-1-12 | 0.05285 | 0.00442 | 0.23701 | 0.01942 | 0.03253 | 0.00055 | 322 | 192 | 216 | 16 | 206 | 3 |
| 12ER34-1-13 | 0.05339 | 0.00272 | 0.27291 | 0.01393 | 0.03706 | 0.00051 | 345 | 91 | 245 | 11 | 235 | 3 |
| 12ER34-1-14 | 0.05567 | 0.00314 | 0.24554 | 0.01363 | 0.03258 | 0.00054 | 439 | 94 | 223 | 11 | 207 | 3 |
| 12ER34-1-15 | 0.05352 | 0.00397 | 0.23932 | 0.01650 | 0.03291 | 0.00060 | 351 | 123 | 218 | 14 | 209 | 4 |
| 12ER34-1-16 | 0.05397 | 0.00285 | 0.24026 | 0.01269 | 0.03242 | 0.00045 | 370 | 94 | 219 | 10 | 206 | 3 |
| 12ER34-1-17 | 0.05543 | 0.00269 | 0.24817 | 0.01154 | 0.03281 | 0.00043 | 430 | 80 | 225 | 9 | 208 | 3 |
| 12ER34-1-18 | 0.04712 | 0.00239 | 0.21102 | 0.01006 | 0.03274 | 0.00041 | 55 | 81 | 194 | 8 | 208 | 3 |
| 12ER34-1-19 | 0.04637 | 0.00214 | 0.21191 | 0.00965 | 0.03284 | 0.00038 | 17 | 75 | 195 | 8 | 208 | 2 |
| 12ER34-1-20 | 0.04893 | 0.00325 | 0.21314 | 0.01342 | 0.03181 | 0.00059 | 145 | 107 | 196 | 11 | 202 | 4 |
| 12ER34-1-21 | 0.04840 | 0.00238 | 0.22363 | 0.01102 | 0.03297 | 0.00056 | 119 | 81 | 205 | 9 | 209 | 3 |
| 12ER34-1-22 | 0.04796 | 0.00184 | 0.21986 | 0.00813 | 0.03295 | 0.00036 | 97 | 64 | 202 | 7 | 209 | 2 |
| 12ER34-1-23 | 0.05673 | 0.00304 | 0.25571 | 0.01346 | 0.03246 | 0.00049 | 481 | 90 | 231 | 11 | 206 | 3 |
| 12ER34-1-24 | 0.04975 | 0.00233 | 0.22164 | 0.00965 | 0.03264 | 0.00042 | 183 | 77 | 203 | 8 | 207 | 3 |
| 11ER26-1-01 | 0.05086 | 0.00202 | 0.23005 | 0.00916 | 0.03254 | 0.00037 | 234 | 71 | 210 | 8 | 206 | 2 |
| 11ER26-1-02 | 0.05071 | 0.00189 | 0.24634 | 0.00894 | 0.03511 | 0.00037 | 227 | 64 | 224 | 7 | 222 | 2 |
| 11ER26-1-03 | 0.05036 | 0.00206 | 0.22680 | 0.00919 | 0.03258 | 0.00039 | 212 | 71 | 208 | 8 | 207 | 2 |
| 11ER26-1-04 | 0.05041 | 0.00155 | 0.22791 | 0.00707 | 0.03256 | 0.00034 | 214 | 52 | 208 | 6 | 207 | 2 |
| 11ER26-1-05 | 0.05353 | 0.00302 | 0.25147 | 0.01334 | 0.03444 | 0.00050 | 351 | 94 | 228 | 11 | 218 | 3 |
| 11ER26-1-06 | 0.04689 | 0.00263 | 0.20621 | 0.01117 | 0.03216 | 0.00047 | 44 | 90 | 190 | 9 | 204 | 3 |
| 11ER26-1-07 | 0.05619 | 0.00371 | 0.26283 | 0.01577 | 0.03444 | 0.00056 | 460 | 104 | 237 | 13 | 218 | 3 |
| 11ER26-1-08 | 0.05087 | 0.00244 | 0.24355 | 0.01113 | 0.03487 | 0.00049 | 235 | 80 | 221 | 9 | 221 | 3 |
| 11ER26-1-09 | 0.05231 | 0.00237 | 0.25450 | 0.01152 | 0.03510 | 0.00041 | 299 | 82 | 230 | 9 | 222 | 3 |
| 11ER26-1-10 | 0.05246 | 0.00208 | 0.25262 | 0.00951 | 0.03496 | 0.00042 | 306 | 64 | 229 | 8 | 222 | 3 |
| 11ER26-1-11 | 0.04888 | 0.00204 | 0.23610 | 0.00971 | 0.03484 | 0.00039 | 142 | 75 | 215 | 8 | 221 | 2 |
| 11ER26-1-12 | 0.05290 | 0.00402 | 0.25107 | 0.01699 | 0.03525 | 0.00061 | 324 | 123 | 227 | 14 | 223 | 4 |
| 11ER26-1-13 | 0.05269 | 0.00397 | 0.25612 | 0.01949 | 0.03522 | 0.00065 | 315 | 140 | 232 | 16 | 223 | 4 |
| 11ER26-1-14 | 0.04911 | 0.00189 | 0.22242 | 0.00856 | 0.03248 | 0.00036 | 153 | 69 | 204 | 7 | 206 | 2 |
| 12ER16-3-01 | 0.04957 | 0.00160 | 0.22379 | 0.00715 | 0.03249 | 0.00030 | 175 | 58 | 205 | 6 | 206 | 2 |
| 12ER16-3-02 | 0.04707 | 0.00202 | 0.25967 | 0.01104 | 0.04013 | 0.00070 | 53 | 62 | 234 | 9 | 254 | 4 |
| 12ER16-3-03 | 0.05451 | 0.00187 | 0.23851 | 0.00799 | 0.03151 | 0.00031 | 392 | 57 | 217 | 7 | 200 | 2 |
| 12ER16-3-04 | 0.05083 | 0.00316 | 0.22408 | 0.01359 | 0.03198 | 0.00044 | 233 | 144 | 205 | 11 | 203 | 3 |
| 12ER16-3-05 | 0.05436 | 0.00265 | 0.29648 | 0.01382 | 0.03963 | 0.00052 | 386 | 81 | 264 | 11 | 251 | 3 |
| 12ER16-3-06 | 0.05794 | 0.00306 | 0.31792 | 0.01616 | 0.04025 | 0.00068 | 527 | 81 | 280 | 12 | 254 | 4 |
| 12ER16-3-07 | 0.06819 | 0.00178 | 1.37173 | 0.03576 | 0.14427 | 0.00157 | 874 | 36 | 877 | 15 | 869 | 9 |
| 12ER16-3-08 | 0.04883 | 0.00196 | 0.22155 | 0.00841 | 0.03284 | 0.00040 | 139 | 66 | 203 | 7 | 208 | 3 |
| 12ER16-3-09 | 0.04897 | 0.00161 | 0.22101 | 0.00713 | 0.03235 | 0.00031 | 146 | 57 | 203 | 6 | 205 | 2 |
| 12ER16-3-10 | 0.05151 | 0.00209 | 0.23684 | 0.00978 | 0.03299 | 0.00050 | 264 | 67 | 216 | 8 | 209 | 3 |
| 12ER16-3-11 | 0.05328 | 0.00191 | 0.27324 | 0.00953 | 0.03684 | 0.00041 | 341 | 59 | 245 | 8 | 233 | 3 |
| 12ER16-3-12 | 0.05439 | 0.00185 | 0.29979 | 0.00992 | 0.03973 | 0.00049 | 387 | 52 | 266 | 8 | 251 | 3 |
| 12ER16-3-13 | 0.05422 | 0.00248 | 0.44373 | 0.01953 | 0.05935 | 0.00075 | 380 | 106 | 373 | 14 | 372 | 5 |
| 12ER16-3-14 | 0.06315 | 0.00241 | 0.55161 | 0.02037 | 0.06335 | 0.00062 | 713 | 83 | 446 | 13 | 396 | 4 |
| 12ER16-3-15 | 0.05607 | 0.00188 | 0.25523 | 0.00845 | 0.03292 | 0.00033 | 455 | 56 | 231 | 7 | 209 | 2 |
| 12ER16-3-16 | 0.05210 | 0.00263 | 0.28794 | 0.01380 | 0.04008 | 0.00064 | 290 | 118 | 257 | 11 | 253 | 4 |
| 12ER16-3-17 | 0.05604 | 0.00185 | 0.46582 | 0.01592 | 0.05998 | 0.00080 | 454 | 52 | 388 | 11 | 376 | 5 |
| 12ER16-3-18 | 0.07507 | 0.00193 | 1.65596 | 0.04462 | 0.15884 | 0.00166 | 1070 | 37 | 992 | 17 | 950 | 9 |
| 12ER16-3-19 | 0.05400 | 0.00195 | 0.24390 | 0.00895 | 0.03264 | 0.00038 | 371 | 61 | 222 | 7 | 207 | 2 |
| 12ER16-3-20 | 0.05966 | 0.00577 | 0.26556 | 0.02528 | 0.03228 | 0.00055 | 591 | 218 | 239 | 20 | 205 | 3 |
| 18ER19-1-01 | 0.05402 | 0.00396 | 0.24670 | 0.01789 | 0.03312 | 0.00034 | 372 | 169 | 224 | 15 | 210 | 2 |
| 18ER19-1-02 | 0.05297 | 0.00227 | 0.24370 | 0.01017 | 0.03363 | 0.00038 | 327 | 74 | 221 | 8 | 213 | 2 |
| 18ER19-1-03 | 0.04775 | 0.00168 | 0.21876 | 0.00786 | 0.03319 | 0.00036 | 87 | 62 | 201 | 7 | 210 | 2 |
| 18ER19-1-04 | 0.05193 | 0.00253 | 0.23651 | 0.01063 | 0.03342 | 0.00037 | 282 | 83 | 216 | 9 | 212 | 2 |
| 18ER19-1-05 | 0.04935 | 0.00213 | 0.22214 | 0.00905 | 0.03303 | 0.00039 | 165 | 73 | 204 | 8 | 209 | 2 |
| 18ER19-1-06 | 0.05082 | 0.00170 | 0.23267 | 0.00739 | 0.03360 | 0.00045 | 233 | 49 | 212 | 6 | 213 | 3 |
| 18ER19-1-07 | 0.05583 | 0.00266 | 0.25482 | 0.01195 | 0.03339 | 0.00042 | 446 | 82 | 230 | 10 | 212 | 3 |
| 18ER19-1-08 | 0.05197 | 0.00220 | 0.23968 | 0.00976 | 0.03357 | 0.00039 | 284 | 72 | 218 | 8 | 213 | 2 |
| 18ER19-1-09 | 0.04927 | 0.00121 | 0.22836 | 0.00577 | 0.03349 | 0.00033 | 161 | 41 | 209 | 5 | 212 | 2 |
| 18ER19-1-10 | 0.05110 | 0.00292 | 0.23500 | 0.01294 | 0.03336 | 0.00041 | 245 | 105 | 214 | 11 | 212 | 3 |
| 18ER19-1-12 | 0.04956 | 0.00232 | 0.22674 | 0.01031 | 0.03303 | 0.00046 | 174 | 79 | 208 | 9 | 209 | 3 |
| 18ER19-1-13 | 0.04947 | 0.00176 | 0.22787 | 0.00792 | 0.03320 | 0.00038 | 170 | 60 | 208 | 7 | 211 | 2 |
| 18ER19-1-14 | 0.05151 | 0.00164 | 0.23596 | 0.00751 | 0.03296 | 0.00030 | 264 | 56 | 215 | 6 | 209 | 2 |
| 18ER19-1-15 | 0.05412 | 0.00293 | 0.25181 | 0.01373 | 0.03372 | 0.00047 | 376 | 98 | 228 | 11 | 214 | 3 |
| 18ER19-1-16 | 0.05235 | 0.00194 | 0.24032 | 0.00834 | 0.03307 | 0.00033 | 301 | 61 | 219 | 7 | 210 | 2 |
| 18ER19-1-17 | 0.05392 | 0.00221 | 0.24670 | 0.00982 | 0.03320 | 0.00039 | 368 | 69 | 224 | 8 | 211 | 2 |
| 18ER19-1-18 | 0.05349 | 0.00242 | 0.24443 | 0.01078 | 0.03306 | 0.00043 | 350 | 76 | 222 | 9 | 210 | 3 |
| 18ER19-1-19 | 0.04740 | 0.00227 | 0.22021 | 0.01044 | 0.03363 | 0.00036 | 69 | 84 | 202 | 9 | 213 | 2 |
| 18ER19-1-20 | 0.05050 | 0.00287 | 0.25658 | 0.01514 | 0.03659 | 0.00037 | 218 | 117 | 232 | 12 | 232 | 2 |
| 18ER19-1-21 | 0.05358 | 0.00172 | 0.24420 | 0.00767 | 0.03311 | 0.00035 | 354 | 52 | 222 | 6 | 210 | 2 |
| 18ER19-1-22 | 0.05288 | 0.00256 | 0.23999 | 0.01137 | 0.03305 | 0.00044 | 324 | 84 | 218 | 9 | 210 | 3 |
| 18ER19-1-23 | 0.04942 | 0.00253 | 0.22358 | 0.01094 | 0.03320 | 0.00039 | 168 | 91 | 205 | 9 | 211 | 2 |
| 18ER19-1-24 | 0.05531 | 0.00280 | 0.25377 | 0.01277 | 0.03346 | 0.00046 | 425 | 88 | 230 | 10 | 212 | 3 |
| 18ER19-1-25 | 0.05256 | 0.00185 | 0.24405 | 0.00856 | 0.03366 | 0.00033 | 310 | 62 | 222 | 7 | 213 | 2 |
| 11ER9-1-01 | 0.05820 | 0.00255 | 0.28440 | 0.01194 | 0.03540 | 0.00042 | 537 | 71 | 254 | 9 | 224 | 3 |
| 11ER9-1-02 | 0.06198 | 0.00500 | 0.34145 | 0.02721 | 0.03995 | 0.00052 | 674 | 179 | 298 | 21 | 253 | 3 |
| 11ER9-1-03 | 0.05828 | 0.00393 | 0.31205 | 0.02036 | 0.03968 | 0.00072 | 540 | 111 | 276 | 16 | 251 | 4 |
| 11ER9-1-04 | 0.05357 | 0.00360 | 0.25429 | 0.01676 | 0.03443 | 0.00046 | 353 | 156 | 230 | 14 | 218 | 3 |
| 11ER9-1-05 | 0.05439 | 0.00374 | 0.30349 | 0.02118 | 0.04080 | 0.00073 | 387 | 125 | 269 | 16 | 258 | 5 |
| 11ER9-1-06 | 0.05989 | 0.00423 | 0.33325 | 0.02305 | 0.04035 | 0.00059 | 600 | 158 | 292 | 18 | 255 | 4 |
| 11ER9-1-07 | 0.05072 | 0.00397 | 0.27755 | 0.02132 | 0.03969 | 0.00059 | 228 | 179 | 249 | 17 | 251 | 4 |
| 11ER9-1-08 | 0.05378 | 0.00434 | 0.29871 | 0.02363 | 0.04028 | 0.00065 | 362 | 186 | 265 | 18 | 255 | 4 |
| 11ER9-1-09 | 0.06440 | 0.00181 | 1.17921 | 0.03280 | 0.13134 | 0.00136 | 755 | 41 | 791 | 15 | 796 | 8 |
| 11ER9-1-10 | 0.05431 | 0.00290 | 0.30135 | 0.01567 | 0.04029 | 0.00068 | 384 | 86 | 267 | 12 | 255 | 4 |
| 11ER9-1-11 | 0.05375 | 0.00206 | 0.26859 | 0.01069 | 0.03572 | 0.00047 | 360 | 66 | 242 | 9 | 226 | 3 |
| 11ER9-1-12 | 0.06696 | 0.00671 | 0.37666 | 0.03721 | 0.04080 | 0.00068 | 837 | 217 | 325 | 27 | 258 | 4 |
| 11ER9-1-13 | 0.07790 | 0.00291 | 0.37956 | 0.01350 | 0.03504 | 0.00047 | 1144 | 49 | 327 | 10 | 222 | 3 |
| 11ER9-1-14 | 0.06891 | 0.00238 | 0.33814 | 0.01046 | 0.03545 | 0.00054 | 896 | 39 | 296 | 8 | 225 | 3 |
| 11ER9-1-15 | 0.06222 | 0.00265 | 0.30658 | 0.01206 | 0.03565 | 0.00046 | 682 | 62 | 272 | 9 | 226 | 3 |
| 11ER9-1-16 | 0.05187 | 0.00326 | 0.25543 | 0.01545 | 0.03571 | 0.00061 | 280 | 146 | 231 | 12 | 226 | 4 |
| 11ER9-1-17 | 0.05228 | 0.00245 | 0.28852 | 0.01319 | 0.03974 | 0.00056 | 298 | 78 | 257 | 10 | 251 | 3 |
| 11ER9-1-18 | 0.06197 | 0.00267 | 0.70590 | 0.03642 | 0.08161 | 0.00222 | 673 | 65 | 542 | 22 | 506 | 13 |
| 11ER9-1-19 | 0.07264 | 0.00205 | 0.36439 | 0.01128 | 0.03587 | 0.00044 | 1004 | 43 | 315 | 8 | 227 | 3 |
| 11ER9-1-20 | 0.07243 | 0.00303 | 0.34961 | 0.01195 | 0.03569 | 0.00049 | 998 | 47 | 304 | 9 | 226 | 3 |
| 11ER9-1-21 | 0.05758 | 0.00210 | 0.61068 | 0.02139 | 0.07643 | 0.00086 | 514 | 57 | 484 | 13 | 475 | 5 |
| 11ER9-1-22 | 0.06659 | 0.00188 | 1.25712 | 0.03444 | 0.13586 | 0.00147 | 825 | 39 | 827 | 15 | 821 | 8 |
| 11ER9-1-23 | 0.05360 | 0.00214 | 0.29403 | 0.01129 | 0.03989 | 0.00044 | 354 | 67 | 262 | 9 | 252 | 3 |
| 13ER46-1-01 | 0.05303 | 0.00158 | 0.26024 | 0.00760 | 0.03570 | 0.00035 | 330 | 48 | 235 | 6 | 226 | 2 |
| 13ER46-1-02 | 0.05242 | 0.00193 | 0.26105 | 0.00951 | 0.03612 | 0.00035 | 304 | 66 | 236 | 8 | 229 | 2 |
| 13ER46-1-03 | 0.05336 | 0.00168 | 0.26137 | 0.00787 | 0.03570 | 0.00032 | 344 | 52 | 236 | 6 | 226 | 2 |
| 13ER46-1-04 | 0.04947 | 0.00258 | 0.25708 | 0.01335 | 0.03803 | 0.00048 | 170 | 96 | 232 | 11 | 241 | 3 |
| 13ER46-1-05 | 0.05177 | 0.00257 | 0.24702 | 0.01183 | 0.03562 | 0.00053 | 275 | 82 | 224 | 10 | 226 | 3 |
| 13ER46-1-06 | 0.05040 | 0.00236 | 0.26192 | 0.01171 | 0.03805 | 0.00042 | 213 | 83 | 236 | 9 | 241 | 3 |
| 13ER46-1-07 | 0.05455 | 0.00199 | 0.28940 | 0.01006 | 0.03875 | 0.00044 | 394 | 58 | 258 | 8 | 245 | 3 |
| 13ER46-1-08 | 0.05305 | 0.00246 | 0.27618 | 0.01235 | 0.03822 | 0.00053 | 331 | 76 | 248 | 10 | 242 | 3 |
| 13ER46-1-09 | 0.05068 | 0.00192 | 0.27044 | 0.01055 | 0.03863 | 0.00041 | 226 | 71 | 243 | 8 | 244 | 3 |
| 13ER46-1-10 | 0.05385 | 0.00213 | 0.26545 | 0.01002 | 0.03606 | 0.00047 | 365 | 61 | 239 | 8 | 228 | 3 |
| 13ER46-1-11 | 0.05284 | 0.00187 | 0.28151 | 0.01008 | 0.03861 | 0.00042 | 322 | 61 | 252 | 8 | 244 | 3 |
| 13ER46-1-12 | 0.05337 | 0.00190 | 0.26617 | 0.00945 | 0.03617 | 0.00039 | 345 | 60 | 240 | 8 | 229 | 2 |
| 13ER46-1-13 | 0.05074 | 0.00253 | 0.26710 | 0.01334 | 0.03860 | 0.00054 | 229 | 90 | 240 | 11 | 244 | 3 |
| 13ER46-1-14 | 0.05964 | 0.00290 | 0.29453 | 0.01427 | 0.03609 | 0.00047 | 591 | 83 | 262 | 11 | 229 | 3 |
| ER12-1-01 | 0.05077 | 0.00127 | 0.24679 | 0.00584 | 0.03553 | 0.00032 | 231 | 38 | 224 | 5 | 225 | 2 |
| ER12-1-02 | 0.04958 | 0.00084 | 0.24402 | 0.00422 | 0.03585 | 0.00030 | 175 | 25 | 222 | 3 | 227 | 2 |
| ER12-1-03 | 0.05075 | 0.00161 | 0.25137 | 0.00798 | 0.03606 | 0.00050 | 230 | 48 | 228 | 6 | 228 | 3 |
| ER12-1-04 | 0.04923 | 0.00087 | 0.26558 | 0.00515 | 0.03904 | 0.00030 | 159 | 31 | 239 | 4 | 247 | 2 |
| ER12-1-05 | 0.05121 | 0.00127 | 0.27746 | 0.00673 | 0.03924 | 0.00029 | 250 | 42 | 249 | 5 | 248 | 2 |
| ER12-1-06 | 0.05229 | 0.00118 | 0.29809 | 0.00774 | 0.04124 | 0.00051 | 298 | 37 | 265 | 6 | 261 | 3 |
| ER12-1-07 | 0.05366 | 0.00096 | 0.27877 | 0.00595 | 0.03762 | 0.00040 | 357 | 29 | 250 | 5 | 238 | 2 |
| ER12-1-08 | 0.05155 | 0.00192 | 0.25371 | 0.00803 | 0.03616 | 0.00062 | 265 | 42 | 230 | 7 | 229 | 4 |
| ER12-1-09 | 0.05101 | 0.00165 | 0.29222 | 0.01271 | 0.04123 | 0.00070 | 241 | 69 | 260 | 10 | 260 | 4 |
| ER12-1-10 | 0.04838 | 0.00088 | 0.27040 | 0.00600 | 0.03905 | 0.00046 | 118 | 31 | 243 | 5 | 247 | 3 |
| ER12-1-11 | 0.04651 | 0.00161 | 0.26748 | 0.00900 | 0.03945 | 0.00041 | 24 | 52 | 241 | 7 | 249 | 3 |
| ER12-1-12 | 0.04690 | 0.00198 | 0.24984 | 0.01002 | 0.03584 | 0.00048 | 44 | 62 | 226 | 8 | 227 | 3 |
| ER12-1-13 | 0.04825 | 0.00140 | 0.25709 | 0.00709 | 0.03642 | 0.00022 | 112 | 54 | 232 | 6 | 231 | 1 |
| ER12-1-14 | 0.04798 | 0.00129 | 0.24818 | 0.00665 | 0.03579 | 0.00025 | 98 | 50 | 225 | 5 | 227 | 2 |
| ER12-1-15 | 0.04972 | 0.00187 | 0.26654 | 0.01061 | 0.03799 | 0.00030 | 182 | 79 | 240 | 9 | 240 | 2 |
| ER12-1-16 | 0.05045 | 0.00202 | 0.27188 | 0.01181 | 0.03867 | 0.00039 | 216 | 82 | 244 | 9 | 245 | 2 |
| ER12-1-17 | 0.05536 | 0.00207 | 0.29149 | 0.01132 | 0.03829 | 0.00031 | 427 | 73 | 260 | 9 | 242 | 2 |
| ER12-1-18 | 0.05196 | 0.00152 | 0.29475 | 0.00777 | 0.04156 | 0.00046 | 284 | 40 | 262 | 6 | 263 | 3 |
| ER12-1-19 | 0.05244 | 0.00159 | 0.27920 | 0.00852 | 0.03867 | 0.00044 | 305 | 49 | 250 | 7 | 245 | 3 |
| ER12-1-20 | 0.05172 | 0.00156 | 0.27615 | 0.00800 | 0.03878 | 0.00031 | 273 | 52 | 248 | 6 | 245 | 2 |
| ER12-1-21 | 0.05206 | 0.00170 | 0.27157 | 0.00861 | 0.03794 | 0.00038 | 288 | 54 | 244 | 7 | 240 | 2 |
| ER12-1-22 | 0.05163 | 0.00139 | 0.29768 | 0.00819 | 0.04189 | 0.00041 | 269 | 45 | 265 | 6 | 265 | 3 |
| ER12-1-23 | 0.05221 | 0.00160 | 0.27797 | 0.00857 | 0.03866 | 0.00032 | 295 | 55 | 249 | 7 | 245 | 2 |
| ER12-1-24 | 0.05319 | 0.00127 | 0.27703 | 0.00710 | 0.03771 | 0.00034 | 337 | 42 | 248 | 6 | 239 | 2 |
| 15ER12-1-01 | 0.04952 | 0.00319 | 0.25583 | 0.01694 | 0.03721 | 0.00050 | 173 | 125 | 231 | 14 | 236 | 3 |
| 15ER12-1-02 | 0.04960 | 0.00186 | 0.25644 | 0.00922 | 0.03768 | 0.00036 | 176 | 66 | 232 | 7 | 238 | 2 |
| 15ER12-1-03 | 0.04919 | 0.00163 | 0.25559 | 0.00839 | 0.03751 | 0.00033 | 157 | 60 | 231 | 7 | 237 | 2 |
| 15ER12-1-04 | 0.05259 | 0.00360 | 0.27802 | 0.01830 | 0.03858 | 0.00054 | 311 | 125 | 249 | 15 | 244 | 3 |
| 15ER12-1-05 | 0.05023 | 0.00289 | 0.26160 | 0.01543 | 0.03749 | 0.00038 | 206 | 117 | 236 | 12 | 237 | 2 |
| 15ER12-1-06 | 0.05085 | 0.00222 | 0.26128 | 0.01129 | 0.03741 | 0.00039 | 234 | 81 | 236 | 9 | 237 | 2 |
| 15ER12-1-07 | 0.05109 | 0.00297 | 0.26663 | 0.01499 | 0.03800 | 0.00039 | 245 | 111 | 240 | 12 | 240 | 2 |
| 15ER12-1-08 | 0.05076 | 0.00213 | 0.26328 | 0.01115 | 0.03773 | 0.00038 | 230 | 79 | 237 | 9 | 239 | 2 |
| 15ER12-1-09 | 0.05080 | 0.00185 | 0.26500 | 0.00961 | 0.03785 | 0.00032 | 232 | 68 | 239 | 8 | 239 | 2 |
| 15ER12-1-10 | 0.05115 | 0.00253 | 0.26368 | 0.01241 | 0.03776 | 0.00039 | 248 | 90 | 238 | 10 | 239 | 2 |
| 15ER12-1-11 | 0.05048 | 0.00337 | 0.26299 | 0.01696 | 0.03777 | 0.00058 | 217 | 119 | 237 | 14 | 239 | 4 |
| 15ER12-1-12 | 0.05272 | 0.00275 | 0.27765 | 0.01426 | 0.03827 | 0.00046 | 317 | 95 | 249 | 11 | 242 | 3 |
| 15ER12-1-13 | 0.04988 | 0.00280 | 0.26288 | 0.01461 | 0.03814 | 0.00055 | 189 | 101 | 237 | 12 | 241 | 3 |
| 15ER12-1-14 | 0.05023 | 0.00255 | 0.25852 | 0.01276 | 0.03752 | 0.00041 | 205 | 94 | 233 | 10 | 237 | 3 |
| 15ER12-1-15 | 0.05050 | 0.00219 | 0.25812 | 0.01146 | 0.03720 | 0.00032 | 218 | 87 | 233 | 9 | 235 | 2 |
| 15ER12-1-16 | 0.05077 | 0.00217 | 0.25971 | 0.01078 | 0.03732 | 0.00034 | 230 | 79 | 234 | 9 | 236 | 2 |
| 15ER12-1-17 | 0.05014 | 0.00194 | 0.25824 | 0.00998 | 0.03749 | 0.00036 | 202 | 72 | 233 | 8 | 237 | 2 |
| 15ER12-1-18 | 0.05157 | 0.00353 | 0.26293 | 0.01695 | 0.03749 | 0.00066 | 266 | 116 | 237 | 14 | 237 | 4 |
| 15ER12-1-19 | 0.05091 | 0.00226 | 0.26090 | 0.01074 | 0.03756 | 0.00041 | 237 | 75 | 235 | 9 | 238 | 3 |
| 15ER12-1-20 | 0.05150 | 0.00276 | 0.26946 | 0.01457 | 0.03794 | 0.00047 | 263 | 102 | 242 | 12 | 240 | 3 |
| 15ER4-1-01 | 0.05235 | 0.00207 | 0.30240 | 0.01510 | 0.04101 | 0.00062 | 301 | 86 | 268 | 12 | 259 | 4 |
| 15ER4-1-02 | 0.05504 | 0.00302 | 0.30822 | 0.01666 | 0.04075 | 0.00053 | 414 | 98 | 273 | 13 | 257 | 3 |
| 15ER4-1-03 | 0.05198 | 0.00193 | 0.31621 | 0.01133 | 0.04390 | 0.00039 | 285 | 66 | 279 | 9 | 277 | 2 |
| 15ER4-1-04 | 0.05282 | 0.00124 | 0.27138 | 0.00643 | 0.03706 | 0.00033 | 321 | 38 | 244 | 5 | 235 | 2 |
| 15ER4-1-05 | 0.05119 | 0.00202 | 0.27529 | 0.01099 | 0.03866 | 0.00040 | 250 | 73 | 247 | 9 | 245 | 2 |
| 15ER4-1-06 | 0.05084 | 0.00158 | 0.26368 | 0.00818 | 0.03733 | 0.00028 | 234 | 58 | 238 | 7 | 236 | 2 |
| 15ER4-1-07 | 0.05246 | 0.00170 | 0.27671 | 0.00969 | 0.03793 | 0.00055 | 306 | 53 | 248 | 8 | 240 | 3 |
| 15ER4-1-08 | 0.05164 | 0.00115 | 0.29167 | 0.00636 | 0.04082 | 0.00026 | 270 | 38 | 260 | 5 | 258 | 2 |
| 15ER4-1-09 | 0.05187 | 0.00154 | 0.27363 | 0.00818 | 0.03816 | 0.00037 | 280 | 51 | 246 | 7 | 241 | 2 |
| 15ER4-1-10 | 0.05412 | 0.00357 | 0.27630 | 0.01793 | 0.03703 | 0.00045 | 376 | 153 | 248 | 14 | 234 | 3 |
| 15ER4-1-11 | 0.05050 | 0.00255 | 0.25779 | 0.01279 | 0.03703 | 0.00036 | 218 | 118 | 233 | 10 | 234 | 2 |
| 15ER4-1-12 | 0.05461 | 0.00136 | 0.29153 | 0.00778 | 0.03847 | 0.00039 | 396 | 42 | 260 | 6 | 243 | 2 |
| 15ER4-1-13 | 0.05154 | 0.00226 | 0.26460 | 0.01089 | 0.03723 | 0.00056 | 265 | 103 | 238 | 9 | 236 | 3 |
| 15ER4-1-14 | 0.05813 | 0.00147 | 0.30835 | 0.00830 | 0.03820 | 0.00034 | 535 | 43 | 273 | 6 | 242 | 2 |
| 15ER4-1-15 | 0.05603 | 0.00181 | 0.32008 | 0.01020 | 0.04145 | 0.00037 | 454 | 55 | 282 | 8 | 262 | 2 |
| 15ER4-1-16 | 0.05302 | 0.00245 | 0.29792 | 0.01333 | 0.04075 | 0.00046 | 330 | 107 | 265 | 10 | 258 | 3 |
| 15ER4-1-17 | 0.05647 | 0.00243 | 0.31561 | 0.01276 | 0.04053 | 0.00060 | 471 | 98 | 279 | 10 | 256 | 4 |
| ER15-1-01 | 0.04728 | 0.00088 | 0.24837 | 0.00490 | 0.03810 | 0.00024 | 63 | 35 | 225 | 4 | 241 | 2 |
| ER15-1-02 | 0.05171 | 0.00278 | 0.27194 | 0.01459 | 0.03812 | 0.00048 | 273 | 100 | 244 | 12 | 241 | 3 |
| ER15-1-03 | 0.04762 | 0.00148 | 0.25113 | 0.00796 | 0.03817 | 0.00041 | 80 | 53 | 227 | 6 | 241 | 3 |
| ER15-1-04 | 0.04927 | 0.00110 | 0.25881 | 0.00535 | 0.03822 | 0.00053 | 161 | 24 | 234 | 4 | 242 | 3 |
| ER15-1-05 | 0.05477 | 0.00098 | 0.28735 | 0.00631 | 0.03797 | 0.00040 | 403 | 31 | 256 | 5 | 240 | 2 |
| ER15-1-06 | 0.04491 | 0.00089 | 0.23547 | 0.00524 | 0.03803 | 0.00030 | -24 | 29 | 215 | 4 | 241 | 2 |
| ER15-1-07 | 0.05457 | 0.00126 | 0.29482 | 0.00794 | 0.03810 | 0.00085 | 395 | 27 | 262 | 6 | 241 | 5 |
| ER15-1-08 | 0.04404 | 0.00117 | 0.24018 | 0.00654 | 0.03799 | 0.00028 | -69 | 45 | 219 | 5 | 240 | 2 |
| ER15-1-09 | 0.04577 | 0.00129 | 0.25400 | 0.00665 | 0.03824 | 0.00030 | -14 | 38 | 230 | 5 | 242 | 2 |
| ER15-1-10 | 0.04491 | 0.00100 | 0.24885 | 0.00529 | 0.03813 | 0.00026 | -24 | 29 | 226 | 4 | 241 | 2 |
| ER15-1-11 | 0.05115 | 0.00185 | 0.27453 | 0.01043 | 0.03815 | 0.00028 | 248 | 75 | 246 | 8 | 241 | 2 |
| ER15-1-12 | 0.04965 | 0.00179 | 0.26398 | 0.01012 | 0.03827 | 0.00033 | 179 | 73 | 238 | 8 | 242 | 2 |
| ER15-1-13 | 0.05247 | 0.00259 | 0.27493 | 0.01325 | 0.03800 | 0.00040 | 306 | 115 | 247 | 11 | 240 | 2 |
| ER15-1-14 | 0.04836 | 0.00153 | 0.25408 | 0.00842 | 0.03815 | 0.00058 | 117 | 50 | 230 | 7 | 241 | 4 |
| ER15-1-15 | 0.04991 | 0.00265 | 0.26068 | 0.01271 | 0.03816 | 0.00047 | 191 | 90 | 235 | 10 | 241 | 3 |
| ER15-1-16 | 0.05230 | 0.00160 | 0.27446 | 0.00906 | 0.03798 | 0.00044 | 298 | 54 | 246 | 7 | 240 | 3 |
| 11ER17-1-01 | 0.05486 | 0.00246 | 0.31368 | 0.01384 | 0.04153 | 0.00055 | 407 | 75 | 277 | 11 | 262 | 3 |
| 11ER17-1-02 | 0.05492 | 0.00183 | 0.28933 | 0.00929 | 0.03799 | 0.00038 | 409 | 54 | 258 | 7 | 240 | 2 |
| 11ER17-1-03 | 0.04868 | 0.00209 | 0.28182 | 0.01238 | 0.04170 | 0.00051 | 133 | 78 | 252 | 10 | 263 | 3 |
| 11ER17-1-04 | 0.05616 | 0.00295 | 0.32466 | 0.01642 | 0.04185 | 0.00055 | 459 | 89 | 285 | 13 | 264 | 3 |
| 11ER17-1-06 | 0.05064 | 0.00254 | 0.26981 | 0.01293 | 0.03864 | 0.00058 | 225 | 117 | 243 | 10 | 244 | 4 |
| 11ER17-1-07 | 0.05388 | 0.00195 | 0.28377 | 0.00911 | 0.03863 | 0.00061 | 366 | 44 | 254 | 7 | 244 | 4 |
| 11ER17-1-08 | 0.05586 | 0.00143 | 0.37974 | 0.00933 | 0.04897 | 0.00050 | 447 | 36 | 327 | 7 | 308 | 3 |
| 11ER17-1-10 | 0.05485 | 0.00319 | 0.35942 | 0.02017 | 0.04753 | 0.00072 | 406 | 134 | 312 | 15 | 299 | 4 |
| 11ER17-1-11 | 0.04786 | 0.00296 | 0.27487 | 0.01666 | 0.04210 | 0.00071 | 92 | 103 | 247 | 13 | 266 | 4 |
| 11ER17-1-12 | 0.05324 | 0.00190 | 0.30958 | 0.01098 | 0.04184 | 0.00046 | 339 | 60 | 274 | 9 | 264 | 3 |
| 11ER17-1-14 | 0.05009 | 0.00232 | 0.29022 | 0.01298 | 0.04203 | 0.00062 | 199 | 76 | 259 | 10 | 265 | 4 |
| 11ER17-1-15 | 0.05041 | 0.00383 | 0.25920 | 0.01928 | 0.03729 | 0.00060 | 214 | 174 | 234 | 16 | 236 | 4 |
| 11ER17-1-16 | 0.05206 | 0.00336 | 0.30138 | 0.02007 | 0.04156 | 0.00077 | 288 | 118 | 267 | 16 | 262 | 5 |
| 11ER17-1-17 | 0.05030 | 0.00446 | 0.29336 | 0.02503 | 0.04230 | 0.00102 | 209 | 202 | 261 | 20 | 267 | 6 |
| 11ER17-1-19 | 0.05017 | 0.00245 | 0.29416 | 0.01450 | 0.04242 | 0.00080 | 203 | 79 | 262 | 11 | 268 | 5 |
| 11ER17-1-21 | 0.05153 | 0.00211 | 0.27835 | 0.01093 | 0.03871 | 0.00042 | 265 | 70 | 249 | 9 | 245 | 3 |
| 13ER31-1-01 | 0.05183 | 0.00105 | 0.27725 | 0.00541 | 0.03877 | 0.00030 | 278 | 30 | 248 | 4 | 245 | 2 |
| 13ER31-1-02 | 0.05199 | 0.00131 | 0.28173 | 0.00699 | 0.03924 | 0.00035 | 285 | 40 | 252 | 6 | 248 | 2 |
| 13ER31-1-03 | 0.05290 | 0.00128 | 0.28351 | 0.00657 | 0.03880 | 0.00030 | 325 | 38 | 253 | 5 | 245 | 2 |
| 13ER31-1-04 | 0.05530 | 0.00133 | 0.29661 | 0.00711 | 0.03872 | 0.00032 | 424 | 39 | 264 | 6 | 245 | 2 |
| 13ER31-1-05 | 0.05166 | 0.00158 | 0.27648 | 0.00831 | 0.03872 | 0.00038 | 271 | 51 | 248 | 7 | 245 | 2 |
| 13ER31-1-06 | 0.05353 | 0.00170 | 0.28684 | 0.00955 | 0.03858 | 0.00038 | 351 | 57 | 256 | 8 | 244 | 2 |
| 13ER31-1-07 | 0.05326 | 0.00140 | 0.28344 | 0.00710 | 0.03859 | 0.00030 | 340 | 43 | 253 | 6 | 244 | 2 |
| 13ER31-1-08 | 0.05093 | 0.00101 | 0.27221 | 0.00540 | 0.03859 | 0.00027 | 238 | 33 | 244 | 4 | 244 | 2 |
| 13ER31-1-09 | 0.04899 | 0.00110 | 0.26146 | 0.00596 | 0.03854 | 0.00030 | 147 | 39 | 236 | 5 | 244 | 2 |
| 13ER31-1-10 | 0.05165 | 0.00128 | 0.27997 | 0.00687 | 0.03929 | 0.00039 | 270 | 38 | 251 | 5 | 248 | 2 |
| 13ER31-1-11 | 0.05574 | 0.00130 | 0.29723 | 0.00719 | 0.03846 | 0.00034 | 442 | 38 | 264 | 6 | 243 | 2 |
| 13ER31-1-12 | 0.05208 | 0.00110 | 0.27954 | 0.00586 | 0.03881 | 0.00028 | 289 | 35 | 250 | 5 | 245 | 2 |
| 13ER31-1-13 | 0.04980 | 0.00122 | 0.26936 | 0.00660 | 0.03920 | 0.00035 | 186 | 40 | 242 | 5 | 248 | 2 |
| 13ER31-1-14 | 0.05145 | 0.00149 | 0.27827 | 0.00841 | 0.03907 | 0.00040 | 261 | 50 | 249 | 7 | 247 | 3 |
| 13ER31-1-15 | 0.05539 | 0.00109 | 0.30168 | 0.00654 | 0.03922 | 0.00038 | 428 | 31 | 268 | 5 | 248 | 2 |
| 13ER31-1-16 | 0.05411 | 0.00133 | 0.29221 | 0.00711 | 0.03909 | 0.00034 | 376 | 39 | 260 | 6 | 247 | 2 |
| 13ER31-1-17 | 0.05191 | 0.00140 | 0.27727 | 0.00749 | 0.03870 | 0.00034 | 282 | 45 | 248 | 6 | 245 | 2 |
| 13ER31-1-18 | 0.05458 | 0.00131 | 0.29525 | 0.00718 | 0.03909 | 0.00031 | 395 | 40 | 263 | 6 | 247 | 2 |
| 13ER31-1-19 | 0.05165 | 0.00217 | 0.27843 | 0.01149 | 0.03910 | 0.00031 | 270 | 99 | 249 | 9 | 247 | 2 |
| 13ER31-1-20 | 0.05248 | 0.00132 | 0.28068 | 0.00679 | 0.03882 | 0.00029 | 306 | 42 | 251 | 5 | 246 | 2 |
| 11ER18-1-02 | 0.05076 | 0.00148 | 0.27123 | 0.00790 | 0.03849 | 0.00039 | 230 | 48 | 244 | 6 | 243 | 2 |
| 11ER18-1-03 | 0.05048 | 0.00173 | 0.26966 | 0.00919 | 0.03866 | 0.00046 | 217 | 57 | 242 | 7 | 245 | 3 |
| 11ER18-1-04 | 0.05335 | 0.00189 | 0.28945 | 0.01022 | 0.03939 | 0.00056 | 344 | 54 | 258 | 8 | 249 | 3 |
| 11ER18-1-05 | 0.05351 | 0.00152 | 0.28944 | 0.00809 | 0.03904 | 0.00048 | 350 | 41 | 258 | 6 | 247 | 3 |
| 11ER18-1-06 | 0.04958 | 0.00166 | 0.27359 | 0.00935 | 0.03968 | 0.00050 | 175 | 56 | 246 | 7 | 251 | 3 |
| 11ER18-1-08 | 0.05527 | 0.00184 | 0.29951 | 0.00940 | 0.03922 | 0.00048 | 423 | 48 | 266 | 7 | 248 | 3 |
| 11ER18-1-10 | 0.04913 | 0.00190 | 0.26896 | 0.01033 | 0.03948 | 0.00047 | 154 | 68 | 242 | 8 | 250 | 3 |
| 11ER18-1-11 | 0.05761 | 0.00189 | 0.31221 | 0.00946 | 0.03930 | 0.00042 | 515 | 48 | 276 | 7 | 248 | 3 |
| 11ER18-1-12 | 0.05489 | 0.00173 | 0.30225 | 0.00994 | 0.03963 | 0.00048 | 408 | 52 | 268 | 8 | 251 | 3 |
| 11ER18-1-13 | 0.05336 | 0.00182 | 0.28729 | 0.01012 | 0.03878 | 0.00045 | 344 | 59 | 256 | 8 | 245 | 3 |
| 11ER18-1-14 | 0.05247 | 0.00212 | 0.28601 | 0.01152 | 0.03956 | 0.00061 | 306 | 64 | 255 | 9 | 250 | 4 |
| 11ER18-1-17 | 0.05193 | 0.00200 | 0.28234 | 0.01095 | 0.03927 | 0.00044 | 282 | 68 | 253 | 9 | 248 | 3 |
| 11ER18-1-19 | 0.05216 | 0.00178 | 0.27958 | 0.00955 | 0.03873 | 0.00045 | 292 | 56 | 250 | 8 | 245 | 3 |
| 11ER18-1-20 | 0.05449 | 0.00331 | 0.29314 | 0.01731 | 0.03902 | 0.00056 | 391 | 140 | 261 | 14 | 247 | 3 |
| 11ER18-1-21 | 0.05260 | 0.00161 | 0.28241 | 0.00833 | 0.03872 | 0.00042 | 312 | 47 | 253 | 7 | 245 | 3 |
| 14ER16-1-01 | 0.05246 | 0.00141 | 0.29815 | 0.00795 | 0.04083 | 0.00037 | 306 | 44 | 265 | 6 | 258 | 2 |
| 14ER16-1-02 | 0.05457 | 0.00158 | 0.31137 | 0.00908 | 0.04090 | 0.00041 | 395 | 47 | 275 | 7 | 258 | 3 |
| 14ER16-1-03 | 0.05315 | 0.00155 | 0.29844 | 0.00895 | 0.04013 | 0.00036 | 335 | 52 | 265 | 7 | 254 | 2 |
| 14ER16-1-04 | 0.05505 | 0.00141 | 0.31084 | 0.00799 | 0.04041 | 0.00033 | 414 | 43 | 275 | 6 | 255 | 2 |
| 14ER16-1-05 | 0.05071 | 0.00131 | 0.28339 | 0.00707 | 0.04013 | 0.00036 | 228 | 41 | 253 | 6 | 254 | 2 |
| 14ER16-1-06 | 0.05343 | 0.00169 | 0.30217 | 0.00945 | 0.04060 | 0.00038 | 347 | 54 | 268 | 7 | 257 | 2 |
| 14ER16-1-07 | 0.05059 | 0.00128 | 0.28532 | 0.00720 | 0.04033 | 0.00037 | 222 | 41 | 255 | 6 | 255 | 2 |
| 14ER16-1-08 | 0.05149 | 0.00150 | 0.29016 | 0.00823 | 0.04036 | 0.00038 | 263 | 48 | 259 | 6 | 255 | 2 |
| 14ER16-1-09 | 0.05216 | 0.00141 | 0.30628 | 0.00835 | 0.04205 | 0.00039 | 292 | 45 | 271 | 6 | 266 | 2 |
| 14ER16-1-10 | 0.05203 | 0.00133 | 0.31358 | 0.00792 | 0.04333 | 0.00039 | 287 | 41 | 277 | 6 | 273 | 2 |
| 14ER16-1-11 | 0.05181 | 0.00107 | 0.31151 | 0.00640 | 0.04316 | 0.00030 | 277 | 34 | 275 | 5 | 272 | 2 |
| 14ER16-1-12 | 0.05120 | 0.00129 | 0.28543 | 0.00716 | 0.04010 | 0.00030 | 250 | 44 | 255 | 6 | 253 | 2 |
| 14ER16-1-13 | 0.04946 | 0.00123 | 0.28370 | 0.00727 | 0.04139 | 0.00041 | 170 | 41 | 254 | 6 | 261 | 3 |
| 14ER16-1-14 | 0.05079 | 0.00115 | 0.28692 | 0.00700 | 0.04071 | 0.00043 | 231 | 37 | 256 | 6 | 257 | 3 |
| 14ER16-1-15 | 0.05157 | 0.00132 | 0.28751 | 0.00766 | 0.04020 | 0.00035 | 267 | 45 | 257 | 6 | 254 | 2 |
| 15ER1-1-01 | 0.05369 | 0.00178 | 0.36260 | 0.01169 | 0.04880 | 0.00046 | 358 | 56 | 314 | 9 | 307 | 3 |
| 15ER1-1-02 | 0.05011 | 0.00183 | 0.33677 | 0.01245 | 0.04845 | 0.00044 | 200 | 69 | 295 | 9 | 305 | 3 |
| 15ER1-1-03 | 0.05175 | 0.00124 | 0.36336 | 0.00944 | 0.05081 | 0.00070 | 275 | 35 | 315 | 7 | 320 | 4 |
| 15ER1-1-04 | 0.05216 | 0.00170 | 0.37005 | 0.01257 | 0.05112 | 0.00054 | 292 | 58 | 320 | 9 | 321 | 3 |
| 15ER1-1-05 | 0.05390 | 0.00177 | 0.37983 | 0.01235 | 0.05097 | 0.00053 | 367 | 55 | 327 | 9 | 320 | 3 |
| 15ER1-1-06 | 0.05209 | 0.00248 | 0.34535 | 0.01657 | 0.04821 | 0.00068 | 289 | 84 | 301 | 13 | 303 | 4 |
| 15ER1-1-07 | 0.05279 | 0.00168 | 0.37301 | 0.01195 | 0.05108 | 0.00047 | 320 | 56 | 322 | 9 | 321 | 3 |
| 15ER1-1-08 | 0.05082 | 0.00224 | 0.33290 | 0.01413 | 0.04747 | 0.00051 | 233 | 78 | 292 | 11 | 299 | 3 |
| 15ER1-1-09 | 0.05234 | 0.00183 | 0.37501 | 0.01481 | 0.05139 | 0.00071 | 300 | 65 | 323 | 11 | 323 | 4 |
| 15ER1-1-10 | 0.05100 | 0.00155 | 0.36498 | 0.01213 | 0.05178 | 0.00075 | 241 | 50 | 316 | 9 | 325 | 5 |
| 15ER1-1-11 | 0.05202 | 0.00177 | 0.33988 | 0.01140 | 0.04727 | 0.00038 | 286 | 62 | 297 | 9 | 298 | 2 |
| 15ER1-1-12 | 0.05262 | 0.00156 | 0.35228 | 0.01063 | 0.04848 | 0.00039 | 312 | 54 | 306 | 8 | 305 | 2 |
| 15ER1-1-13 | 0.05402 | 0.00181 | 0.36390 | 0.01208 | 0.04880 | 0.00041 | 372 | 60 | 315 | 9 | 307 | 3 |
| 15ER1-1-14 | 0.05218 | 0.00168 | 0.35370 | 0.01182 | 0.04913 | 0.00046 | 293 | 59 | 307 | 9 | 309 | 3 |
| 15ER1-1-15 | 0.05138 | 0.00181 | 0.33773 | 0.01173 | 0.04763 | 0.00042 | 258 | 64 | 295 | 9 | 300 | 3 |
| 15ER1-1-16 | 0.05167 | 0.00330 | 0.34396 | 0.02183 | 0.04830 | 0.00070 | 271 | 119 | 300 | 16 | 304 | 4 |
| 15ER1-1-17 | 0.05256 | 0.00317 | 0.37473 | 0.02140 | 0.05207 | 0.00076 | 310 | 104 | 323 | 16 | 327 | 5 |
| 15ER1-1-18 | 0.05152 | 0.00179 | 0.36905 | 0.01306 | 0.05209 | 0.00067 | 264 | 57 | 319 | 10 | 327 | 4 |
| 15ER1-1-19 | 0.05053 | 0.00174 | 0.33896 | 0.01196 | 0.04877 | 0.00060 | 220 | 59 | 296 | 9 | 307 | 4 |
| 15ER1-1-20 | 0.05283 | 0.00181 | 0.35533 | 0.01247 | 0.04881 | 0.00053 | 322 | 60 | 309 | 9 | 307 | 3 |
| 15ER1-1-21 | 0.05346 | 0.00225 | 0.35386 | 0.01565 | 0.04820 | 0.00076 | 348 | 71 | 308 | 12 | 303 | 5 |
| 15ER1-1-22 | 0.05240 | 0.00200 | 0.34388 | 0.01377 | 0.04725 | 0.00040 | 303 | 76 | 300 | 10 | 298 | 2 |
| 15ER1-1-23 | 0.05173 | 0.00173 | 0.36533 | 0.01201 | 0.05145 | 0.00043 | 274 | 60 | 316 | 9 | 323 | 3 |
| 15ER1-1-24 | 0.05290 | 0.00149 | 0.35024 | 0.00994 | 0.04800 | 0.00041 | 325 | 49 | 305 | 7 | 302 | 3 |
| 15ER1-1-25 | 0.05097 | 0.00195 | 0.36605 | 0.01405 | 0.05228 | 0.00058 | 240 | 68 | 317 | 10 | 328 | 4 |
| 12ER36-1-01 | 0.05799 | 0.00105 | 0.59603 | 0.01009 | 0.07453 | 0.00153 | 529 | 20 | 475 | 6 | 463 | 9 |
| 12ER36-1-02 | 0.05780 | 0.00397 | 0.57985 | 0.03636 | 0.07275 | 0.00266 | 522 | 76 | 464 | 23 | 453 | 16 |
| 12ER36-1-03 | 0.05751 | 0.00107 | 0.58612 | 0.01017 | 0.07391 | 0.00152 | 511 | 20 | 468 | 7 | 460 | 9 |
| 12ER36-1-04 | 0.05825 | 0.00365 | 0.58345 | 0.03336 | 0.07264 | 0.00250 | 539 | 68 | 467 | 21 | 452 | 15 |
| 12ER36-1-05 | 0.05636 | 0.00130 | 0.55644 | 0.01188 | 0.07160 | 0.00154 | 467 | 22 | 449 | 8 | 446 | 9 |
| 12ER36-1-06 | 0.05622 | 0.00147 | 0.56834 | 0.01376 | 0.07333 | 0.00162 | 461 | 24 | 457 | 9 | 456 | 10 |
| 12ER36-1-07 | 0.05550 | 0.00303 | 0.56305 | 0.02827 | 0.07359 | 0.00227 | 432 | 60 | 454 | 18 | 458 | 14 |
| 12ER36-1-08 | 0.05597 | 0.00117 | 0.55967 | 0.01094 | 0.07254 | 0.00152 | 451 | 21 | 451 | 7 | 451 | 9 |
| 12ER36-1-09 | 0.05526 | 0.00107 | 0.55479 | 0.01006 | 0.07286 | 0.00150 | 423 | 21 | 448 | 7 | 453 | 9 |
| 12ER36-1-10 | 0.05574 | 0.00159 | 0.55773 | 0.01138 | 0.07257 | 0.00145 | 442 | 65 | 450 | 7 | 452 | 9 |
| 12ER36-1-11 | 0.05834 | 0.00227 | 0.59379 | 0.02124 | 0.07387 | 0.00190 | 543 | 38 | 473 | 14 | 459 | 11 |
| 12ER36-1-13 | 0.05569 | 0.00139 | 0.57230 | 0.01328 | 0.07459 | 0.00162 | 440 | 23 | 460 | 9 | 464 | 10 |
| 12ER36-1-14 | 0.05589 | 0.00165 | 0.56352 | 0.01539 | 0.07319 | 0.00167 | 448 | 27 | 454 | 10 | 455 | 10 |
| 12ER36-1-15 | 0.05587 | 0.00196 | 0.56420 | 0.01830 | 0.07331 | 0.00177 | 447 | 34 | 454 | 12 | 456 | 11 |
| 12ER36-1-16 | 0.05876 | 0.00246 | 0.59952 | 0.02304 | 0.07408 | 0.00196 | 558 | 42 | 477 | 15 | 461 | 12 |
| 12ER36-1-17 | 0.05836 | 0.00616 | 0.58401 | 0.05829 | 0.07258 | 0.00250 | 543 | 239 | 467 | 37 | 452 | 15 |
| 12ER36-1-19 | 0.05864 | 0.00370 | 0.58124 | 0.03360 | 0.07200 | 0.00245 | 554 | 69 | 465 | 22 | 448 | 15 |
| 12ER36-1-22 | 0.05817 | 0.00203 | 0.58451 | 0.01876 | 0.07301 | 0.00178 | 536 | 33 | 467 | 12 | 454 | 11 |
| 12ER36-1-23 | 0.05683 | 0.00358 | 0.56747 | 0.03278 | 0.07255 | 0.00246 | 485 | 70 | 456 | 21 | 452 | 15 |
| 12ER36-1-24 | 0.05712 | 0.00343 | 0.57219 | 0.03148 | 0.07279 | 0.00238 | 496 | 66 | 459 | 20 | 453 | 14 |
| 18ER23-1-01 | 0.05573 | 0.00123 | 0.57258 | 0.01329 | 0.07421 | 0.00068 | 442 | 35 | 460 | 9 | 461 | 4 |
| 18ER23-1-02 | 0.05643 | 0.00128 | 0.57741 | 0.01344 | 0.07390 | 0.00056 | 469 | 38 | 463 | 9 | 460 | 3 |
| 18ER23-1-03 | 0.05707 | 0.00125 | 0.58484 | 0.01270 | 0.07408 | 0.00058 | 494 | 34 | 468 | 8 | 461 | 3 |
| 18ER23-1-04 | 0.05624 | 0.00111 | 0.57932 | 0.01165 | 0.07435 | 0.00058 | 462 | 31 | 464 | 7 | 462 | 3 |
| 18ER23-1-05 | 0.05463 | 0.00128 | 0.55786 | 0.01279 | 0.07376 | 0.00057 | 397 | 37 | 450 | 8 | 459 | 3 |
| 18ER23-1-06 | 0.05621 | 0.00146 | 0.57303 | 0.01484 | 0.07356 | 0.00063 | 461 | 42 | 460 | 10 | 458 | 4 |
| 18ER23-1-07 | 0.05693 | 0.00125 | 0.58289 | 0.01297 | 0.07369 | 0.00053 | 489 | 36 | 466 | 8 | 458 | 3 |
| 18ER23-1-08 | 0.05628 | 0.00148 | 0.57383 | 0.01492 | 0.07352 | 0.00058 | 463 | 44 | 460 | 10 | 457 | 3 |
| 18ER23-1-09 | 0.05827 | 0.00110 | 0.59842 | 0.01154 | 0.07394 | 0.00052 | 540 | 30 | 476 | 7 | 460 | 3 |
| 18ER23-1-10 | 0.05686 | 0.00129 | 0.57914 | 0.01267 | 0.07349 | 0.00052 | 486 | 36 | 464 | 8 | 457 | 3 |
| 18ER23-1-11 | 0.05769 | 0.00128 | 0.59526 | 0.01309 | 0.07441 | 0.00056 | 518 | 35 | 474 | 8 | 463 | 3 |
| 18ER23-1-12 | 0.05644 | 0.00138 | 0.57512 | 0.01362 | 0.07355 | 0.00055 | 470 | 39 | 461 | 9 | 458 | 3 |
| 18ER23-1-13 | 0.05705 | 0.00118 | 0.58273 | 0.01207 | 0.07359 | 0.00057 | 494 | 32 | 466 | 8 | 458 | 3 |
| 18ER23-1-14 | 0.05673 | 0.00135 | 0.58428 | 0.01359 | 0.07451 | 0.00063 | 481 | 36 | 467 | 9 | 463 | 4 |
| 18ER23-1-15 | 0.05734 | 0.00128 | 0.59080 | 0.01300 | 0.07448 | 0.00064 | 505 | 33 | 471 | 8 | 463 | 4 |
| 18ER23-1-17 | 0.05477 | 0.00117 | 0.56338 | 0.01206 | 0.07447 | 0.00070 | 403 | 31 | 454 | 8 | 463 | 4 |
| 18ER23-1-18 | 0.05683 | 0.00130 | 0.57836 | 0.01327 | 0.07366 | 0.00066 | 485 | 35 | 463 | 9 | 458 | 4 |
| 18ER23-1-19 | 0.05632 | 0.00128 | 0.57598 | 0.01318 | 0.07401 | 0.00060 | 465 | 36 | 462 | 8 | 460 | 4 |
| 18ER23-1-20 | 0.05656 | 0.00134 | 0.61299 | 0.01614 | 0.07812 | 0.00076 | 475 | 41 | 485 | 10 | 485 | 5 |
| 18ER23-1-21 | 0.05706 | 0.00138 | 0.58545 | 0.01421 | 0.07433 | 0.00060 | 494 | 39 | 468 | 9 | 462 | 4 |
| 18ER23-1-22 | 0.05558 | 0.00110 | 0.60085 | 0.01249 | 0.07816 | 0.00073 | 436 | 30 | 478 | 8 | 485 | 4 |
| 18ER23-1-23 | 0.05714 | 0.00119 | 0.58423 | 0.01204 | 0.07391 | 0.00048 | 497 | 34 | 467 | 8 | 460 | 3 |
| 18ER23-1-24 | 0.05556 | 0.00124 | 0.56578 | 0.01238 | 0.07359 | 0.00049 | 435 | 37 | 455 | 8 | 458 | 3 |
| 18ER23-1-25 | 0.05638 | 0.00158 | 0.57512 | 0.01585 | 0.07375 | 0.00062 | 467 | 46 | 461 | 10 | 459 | 4 |
| 14ER10-1-01 | 0.06069 | 0.00170 | 0.56791 | 0.01690 | 0.06719 | 0.00083 | 628 | 43 | 457 | 11 | 419 | 5 |
| 14ER10-1-02 | 0.05610 | 0.00173 | 0.50746 | 0.01490 | 0.06561 | 0.00063 | 456 | 70 | 417 | 10 | 410 | 4 |
| 14ER10-1-03 | 0.06032 | 0.00178 | 0.63540 | 0.01841 | 0.07568 | 0.00083 | 615 | 44 | 499 | 11 | 470 | 5 |
| 14ER10-1-04 | 0.06360 | 0.00312 | 0.57726 | 0.02587 | 0.06583 | 0.00131 | 728 | 107 | 463 | 17 | 411 | 8 |
| 14ER10-1-05 | 0.07031 | 0.00181 | 1.60437 | 0.04749 | 0.16312 | 0.00242 | 937 | 37 | 972 | 19 | 974 | 13 |
| 14ER10-1-06 | 0.06781 | 0.00152 | 1.01375 | 0.02318 | 0.10722 | 0.00107 | 863 | 31 | 711 | 12 | 657 | 6 |
| 14ER10-1-07 | 0.05852 | 0.00120 | 0.55414 | 0.01136 | 0.06789 | 0.00051 | 549 | 31 | 448 | 7 | 423 | 3 |
| 14ER10-1-08 | 0.06464 | 0.00130 | 1.27252 | 0.02495 | 0.14126 | 0.00099 | 763 | 29 | 834 | 11 | 852 | 6 |
| 14ER10-1-09 | 0.05975 | 0.00116 | 0.62052 | 0.01194 | 0.07463 | 0.00068 | 595 | 26 | 490 | 7 | 464 | 4 |
| 14ER10-1-10 | 0.05824 | 0.00107 | 0.60778 | 0.01199 | 0.07493 | 0.00084 | 539 | 24 | 482 | 8 | 466 | 5 |
| 14ER10-1-11 | 0.06014 | 0.00117 | 0.51996 | 0.01039 | 0.06207 | 0.00062 | 609 | 26 | 425 | 7 | 388 | 4 |
| 14ER10-1-12 | 0.06117 | 0.00125 | 0.51640 | 0.01085 | 0.06077 | 0.00076 | 645 | 25 | 423 | 7 | 380 | 5 |
| 14ER10-1-13 | 0.05734 | 0.00154 | 0.62011 | 0.01550 | 0.07843 | 0.00075 | 505 | 60 | 490 | 10 | 487 | 4 |
| 14ER10-1-14 | 0.05650 | 0.00205 | 0.57439 | 0.01860 | 0.07374 | 0.00120 | 472 | 82 | 461 | 12 | 459 | 7 |
| 14ER10-1-15 | 0.05717 | 0.00109 | 0.64107 | 0.01450 | 0.08047 | 0.00113 | 498 | 26 | 503 | 9 | 499 | 7 |
| 14ER6-1-01 | 0.05604 | 0.00125 | 0.60050 | 0.01359 | 0.07726 | 0.00065 | 454 | 35 | 478 | 9 | 480 | 4 |
| 14ER6-1-02 | 0.05726 | 0.00143 | 0.61190 | 0.01546 | 0.07715 | 0.00070 | 502 | 40 | 485 | 10 | 479 | 4 |
| 14ER6-1-03 | 0.06037 | 0.00225 | 0.65080 | 0.02583 | 0.07759 | 0.00095 | 617 | 64 | 509 | 16 | 482 | 6 |
| 14ER6-1-04 | 0.05364 | 0.00135 | 0.56924 | 0.01458 | 0.07656 | 0.00068 | 356 | 42 | 458 | 9 | 476 | 4 |
| 14ER6-1-05 | 0.05564 | 0.00144 | 0.59573 | 0.01568 | 0.07730 | 0.00073 | 438 | 42 | 475 | 10 | 480 | 4 |
| 14ER6-1-06 | 0.05786 | 0.00210 | 0.61781 | 0.02257 | 0.07726 | 0.00084 | 524 | 61 | 488 | 14 | 480 | 5 |
| 14ER6-1-07 | 0.05490 | 0.00155 | 0.57490 | 0.01692 | 0.07533 | 0.00073 | 408 | 48 | 461 | 11 | 468 | 4 |
| 14ER6-1-08 | 0.05630 | 0.00155 | 0.58974 | 0.01621 | 0.07553 | 0.00061 | 464 | 47 | 471 | 10 | 469 | 4 |
| 14ER6-1-09 | 0.05965 | 0.00151 | 0.63550 | 0.01617 | 0.07698 | 0.00070 | 591 | 39 | 500 | 10 | 478 | 4 |
| 14ER6-1-10 | 0.05405 | 0.00207 | 0.56619 | 0.02095 | 0.07641 | 0.00074 | 373 | 66 | 456 | 14 | 475 | 4 |
| 14ER15-1-02 | 0.05624 | 0.00216 | 0.60059 | 0.02222 | 0.07745 | 0.00081 | 462 | 87 | 478 | 14 | 481 | 5 |
| 14ER15-1-03 | 0.05575 | 0.00143 | 0.59547 | 0.01505 | 0.07674 | 0.00067 | 442 | 41 | 474 | 10 | 477 | 4 |
| 14ER15-1-05 | 0.05791 | 0.00127 | 0.62532 | 0.01452 | 0.07729 | 0.00062 | 526 | 37 | 493 | 9 | 480 | 4 |
| 14ER15-1-06 | 0.05462 | 0.00117 | 0.58565 | 0.01296 | 0.07695 | 0.00066 | 397 | 34 | 468 | 8 | 478 | 4 |
| 14ER15-1-07 | 0.05597 | 0.00140 | 0.59703 | 0.01528 | 0.07658 | 0.00071 | 451 | 40 | 475 | 10 | 476 | 4 |
| 14ER15-1-08 | 0.05834 | 0.00205 | 0.62834 | 0.02202 | 0.07755 | 0.00085 | 543 | 57 | 495 | 14 | 481 | 5 |
| 14ER15-1-09 | 0.05611 | 0.00151 | 0.60196 | 0.01586 | 0.07781 | 0.00087 | 457 | 39 | 478 | 10 | 483 | 5 |
| 14ER15-1-10 | 0.05651 | 0.00133 | 0.60958 | 0.01580 | 0.07731 | 0.00082 | 472 | 38 | 483 | 10 | 480 | 5 |
| 14ER15-1-11 | 0.05875 | 0.00200 | 0.61415 | 0.01966 | 0.07581 | 0.00089 | 558 | 76 | 486 | 12 | 471 | 5 |
| 14ER15-1-12 | 0.05951 | 0.00152 | 0.64667 | 0.01715 | 0.07816 | 0.00080 | 586 | 40 | 506 | 11 | 485 | 5 |
| 14ER15-1-13 | 0.05660 | 0.00119 | 0.61176 | 0.01360 | 0.07749 | 0.00067 | 476 | 34 | 485 | 9 | 481 | 4 |
| 14ER15-1-14 | 0.05604 | 0.00128 | 0.60573 | 0.01391 | 0.07754 | 0.00059 | 454 | 37 | 481 | 9 | 481 | 4 |
| 14ER15-1-15 | 0.05637 | 0.00167 | 0.59438 | 0.01710 | 0.07585 | 0.00065 | 467 | 48 | 474 | 11 | 471 | 4 |
| 14ER7-5-01 | 0.05939 | 0.00172 | 0.63841 | 0.01837 | 0.07752 | 0.00071 | 581 | 47 | 501 | 11 | 481 | 4 |
| 14ER7-5-02 | 0.05741 | 0.00161 | 0.64642 | 0.01968 | 0.08074 | 0.00082 | 507 | 49 | 506 | 12 | 501 | 5 |
| 14ER7-5-03 | 0.05826 | 0.00123 | 0.69117 | 0.01517 | 0.08532 | 0.00074 | 539 | 33 | 534 | 9 | 528 | 4 |
| 14ER7-5-04 | 0.05566 | 0.00140 | 0.60082 | 0.01520 | 0.07770 | 0.00064 | 439 | 41 | 478 | 10 | 482 | 4 |
| 14ER7-5-05 | 0.05718 | 0.00182 | 0.63571 | 0.02034 | 0.08059 | 0.00090 | 498 | 51 | 500 | 13 | 500 | 5 |
| 14ER7-5-06 | 0.05606 | 0.00130 | 0.62284 | 0.01473 | 0.08019 | 0.00075 | 455 | 36 | 492 | 9 | 497 | 4 |
| 14ER7-5-07 | 0.05666 | 0.00172 | 0.62611 | 0.01920 | 0.08009 | 0.00088 | 478 | 48 | 494 | 12 | 497 | 5 |
| 14ER7-5-08 | 0.05451 | 0.00136 | 0.58611 | 0.01473 | 0.07758 | 0.00068 | 392 | 41 | 468 | 9 | 482 | 4 |
| 14ER7-5-09 | 0.05611 | 0.00155 | 0.59188 | 0.01665 | 0.07600 | 0.00070 | 457 | 46 | 472 | 11 | 472 | 4 |
| 14ER7-5-10 | 0.05957 | 0.00237 | 0.62047 | 0.02384 | 0.07633 | 0.00088 | 588 | 63 | 490 | 15 | 474 | 5 |
| 14ER7-5-11 | 0.05599 | 0.00152 | 0.59187 | 0.01594 | 0.07646 | 0.00067 | 452 | 44 | 472 | 10 | 475 | 4 |
| 14ER7-5-12 | 0.05503 | 0.00201 | 0.58632 | 0.02160 | 0.07703 | 0.00077 | 414 | 64 | 469 | 14 | 478 | 5 |
| 14ER7-5-13 | 0.06037 | 0.00226 | 0.66215 | 0.02392 | 0.08003 | 0.00090 | 617 | 58 | 516 | 15 | 496 | 5 |
| 14ER7-5-15 | 0.05856 | 0.00197 | 0.62547 | 0.02085 | 0.07734 | 0.00089 | 551 | 53 | 493 | 13 | 480 | 5 |
| 14ER5-1-01 | 0.06009 | 0.00137 | 0.71387 | 0.01759 | 0.08600 | 0.00086 | 607 | 36 | 547 | 10 | 532 | 5 |
| 14ER5-1-02 | 0.05929 | 0.00101 | 0.70893 | 0.01262 | 0.08651 | 0.00065 | 578 | 26 | 544 | 7 | 535 | 4 |
| 14ER5-1-03 | 0.05622 | 0.00169 | 0.62415 | 0.01959 | 0.08037 | 0.00094 | 461 | 49 | 492 | 12 | 498 | 6 |
| 14ER5-1-04 | 0.05816 | 0.00110 | 0.64899 | 0.01224 | 0.08059 | 0.00056 | 536 | 29 | 508 | 8 | 500 | 3 |
| 14ER5-1-05 | 0.05844 | 0.00107 | 0.70218 | 0.01455 | 0.08665 | 0.00100 | 546 | 26 | 540 | 9 | 536 | 6 |
| 14ER5-1-06 | 0.05805 | 0.00150 | 0.62483 | 0.01689 | 0.07756 | 0.00077 | 531 | 42 | 493 | 11 | 482 | 5 |
| 14ER5-1-07 | 0.05885 | 0.00212 | 0.62414 | 0.02169 | 0.07692 | 0.00074 | 561 | 81 | 492 | 14 | 478 | 4 |
| 14ER5-1-08 | 0.05803 | 0.00157 | 0.62421 | 0.01822 | 0.07767 | 0.00098 | 531 | 42 | 492 | 11 | 482 | 6 |
| 14ER5-1-09 | 0.05814 | 0.00135 | 0.64857 | 0.01625 | 0.08068 | 0.00101 | 535 | 33 | 508 | 10 | 500 | 6 |
| 14ER5-1-10 | 0.05871 | 0.00128 | 0.58525 | 0.01290 | 0.07208 | 0.00063 | 556 | 33 | 468 | 8 | 449 | 4 |
| 14ER5-1-11 | 0.05828 | 0.00148 | 0.62659 | 0.01691 | 0.07778 | 0.00084 | 540 | 40 | 494 | 11 | 483 | 5 |
| 14ER5-1-12 | 0.05836 | 0.00116 | 0.63061 | 0.01339 | 0.07815 | 0.00072 | 543 | 30 | 496 | 8 | 485 | 4 |
| 14ER5-1-13 | 0.05996 | 0.00152 | 0.64127 | 0.01757 | 0.07736 | 0.00091 | 602 | 39 | 503 | 11 | 480 | 5 |
| 14ER5-1-14 | 0.05980 | 0.00163 | 0.63814 | 0.01856 | 0.07740 | 0.00101 | 596 | 41 | 501 | 12 | 481 | 6 |
| 14ER5-1-15 | 0.05906 | 0.00126 | 0.65885 | 0.01502 | 0.08059 | 0.00070 | 569 | 34 | 514 | 9 | 500 | 4 |
| 14ER5-1-16 | 0.05758 | 0.00146 | 0.61395 | 0.01624 | 0.07721 | 0.00079 | 514 | 40 | 486 | 10 | 479 | 5 |
| 14ER5-1-17 | 0.05624 | 0.00169 | 0.59672 | 0.01868 | 0.07693 | 0.00082 | 462 | 50 | 475 | 12 | 478 | 5 |
| 14ER5-1-18 | 0.05834 | 0.00136 | 0.64839 | 0.01606 | 0.08083 | 0.00111 | 543 | 31 | 507 | 10 | 501 | 7 |
| 14ER5-1-19 | 0.05850 | 0.00150 | 0.70043 | 0.02446 | 0.08649 | 0.00167 | 549 | 43 | 539 | 15 | 535 | 10 |
| 14ER5-1-20 | 0.05815 | 0.00129 | 0.62076 | 0.01490 | 0.07738 | 0.00072 | 535 | 36 | 490 | 9 | 480 | 4 |
| 14ER5-1-21 | 0.05476 | 0.00105 | 0.60908 | 0.01281 | 0.08080 | 0.00087 | 403 | 28 | 483 | 8 | 501 | 5 |
| 14ER5-1-22 | 0.05650 | 0.00116 | 0.62845 | 0.01330 | 0.08089 | 0.00085 | 472 | 28 | 495 | 8 | 501 | 5 |
| 14ER5-1-23 | 0.05681 | 0.00152 | 0.60764 | 0.01674 | 0.07767 | 0.00077 | 484 | 43 | 482 | 11 | 482 | 5 |
| 14ER5-1-24 | 0.05910 | 0.00115 | 0.54389 | 0.01103 | 0.06674 | 0.00055 | 571 | 30 | 441 | 7 | 416 | 3 |
| 14ER5-1-25 | 0.05551 | 0.00112 | 0.59274 | 0.01346 | 0.07738 | 0.00087 | 433 | 31 | 473 | 9 | 480 | 5 |
| 13ER30-1-01 | 0.05987 | 0.00178 | 0.64307 | 0.01818 | 0.07790 | 0.00074 | 599 | 66 | 504 | 11 | 484 | 4 |
| 13ER30-1-01 | 0.05803 | 0.00080 | 0.62855 | 0.00996 | 0.07834 | 0.00080 | 531 | 18 | 495 | 6 | 486 | 5 |
| 13ER30-1-02 | 0.05567 | 0.00113 | 0.60127 | 0.01285 | 0.07807 | 0.00069 | 439 | 32 | 478 | 8 | 485 | 4 |
| 13ER30-1-03 | 0.06203 | 0.00155 | 0.66585 | 0.01463 | 0.07785 | 0.00094 | 675 | 55 | 518 | 9 | 483 | 6 |
| 13ER30-1-05 | 0.06183 | 0.00102 | 0.66923 | 0.01064 | 0.07839 | 0.00082 | 668 | 17 | 520 | 6 | 486 | 5 |
| 13ER30-1-06 | 0.06057 | 0.00109 | 0.64897 | 0.01098 | 0.07755 | 0.00059 | 624 | 23 | 508 | 7 | 482 | 4 |
| 13ER30-1-07 | 0.05688 | 0.00148 | 0.60338 | 0.01601 | 0.07681 | 0.00075 | 487 | 41 | 479 | 10 | 477 | 5 |
| 13ER30-1-08 | 0.05526 | 0.00136 | 0.59601 | 0.01566 | 0.07784 | 0.00072 | 423 | 42 | 475 | 10 | 483 | 4 |
| 13ER30-1-09 | 0.06333 | 0.00189 | 0.67093 | 0.01937 | 0.07684 | 0.00058 | 719 | 65 | 521 | 12 | 477 | 3 |
| 13ER30-1-10 | 0.05762 | 0.00130 | 0.62102 | 0.01449 | 0.07785 | 0.00070 | 515 | 35 | 490 | 9 | 483 | 4 |
| 13ER30-1-16 | 0.05478 | 0.00075 | 0.59087 | 0.00894 | 0.07788 | 0.00059 | 403 | 21 | 471 | 6 | 483 | 4 |
| 13ER30-1-19 | 0.05824 | 0.00154 | 0.62458 | 0.01493 | 0.07778 | 0.00088 | 539 | 59 | 493 | 9 | 483 | 5 |
| ER13-1-01 | 0.06605 | 0.00157 | 1.25940 | 0.02681 | 0.13830 | 0.00148 | 808 | 51 | 828 | 12 | 835 | 8 |
| ER13-1-02 | 0.06544 | 0.00134 | 1.17640 | 0.02116 | 0.13038 | 0.00129 | 789 | 44 | 790 | 10 | 790 | 7 |
| ER13-1-03 | 0.06535 | 0.00075 | 1.09048 | 0.02083 | 0.12098 | 0.00197 | 786 | 18 | 749 | 10 | 736 | 11 |
| ER13-1-04 | 0.06487 | 0.00058 | 1.23959 | 0.01939 | 0.13845 | 0.00181 | 770 | 15 | 819 | 9 | 836 | 10 |
| ER13-1-05 | 0.06564 | 0.00053 | 1.18973 | 0.01728 | 0.13123 | 0.00154 | 795 | 14 | 796 | 8 | 795 | 9 |
| ER13-1-06 | 0.06566 | 0.00069 | 1.09777 | 0.02392 | 0.12113 | 0.00243 | 796 | 20 | 752 | 12 | 737 | 14 |
| ER13-1-07 | 0.06560 | 0.00071 | 1.25304 | 0.01462 | 0.13841 | 0.00083 | 794 | 15 | 825 | 7 | 836 | 5 |
| ER13-1-08 | 0.06505 | 0.00103 | 1.08703 | 0.02603 | 0.12114 | 0.00262 | 776 | 22 | 747 | 13 | 737 | 15 |
| ER13-1-09 | 0.06580 | 0.00090 | 1.09710 | 0.01909 | 0.12098 | 0.00180 | 800 | 16 | 752 | 9 | 736 | 10 |
| ER13-1-11 | 0.06736 | 0.00082 | 1.28675 | 0.02204 | 0.13838 | 0.00194 | 849 | 16 | 840 | 10 | 836 | 11 |
| ER13-1-12 | 0.06541 | 0.00119 | 1.09304 | 0.01710 | 0.12119 | 0.00111 | 788 | 39 | 750 | 8 | 737 | 6 |
| ER13-1-13 | 0.06678 | 0.00061 | 1.27750 | 0.01837 | 0.13845 | 0.00151 | 831 | 14 | 836 | 8 | 836 | 9 |
| ER13-1-14 | 0.06745 | 0.00086 | 1.28679 | 0.02407 | 0.13817 | 0.00206 | 852 | 18 | 840 | 11 | 834 | 12 |
| ER13-1-15 | 0.06640 | 0.00076 | 1.26672 | 0.01578 | 0.13830 | 0.00171 | 819 | 12 | 831 | 7 | 835 | 10 |
| ER13-1-22 | 0.06409 | 0.00143 | 1.07133 | 0.02110 | 0.12124 | 0.00125 | 745 | 48 | 739 | 10 | 738 | 7 |
| ER13-1-24 | 0.06557 | 0.00055 | 1.18501 | 0.01440 | 0.13086 | 0.00126 | 793 | 12 | 794 | 7 | 793 | 7 |
| ER13-1-25 | 0.06707 | 0.00165 | 1.11801 | 0.02353 | 0.12090 | 0.00153 | 840 | 52 | 762 | 11 | 736 | 9 |
| ER13-1-27 | 0.06476 | 0.00140 | 1.08102 | 0.01918 | 0.12107 | 0.00151 | 767 | 47 | 744 | 9 | 737 | 9 |
| ER13-1-28 | 0.06614 | 0.00078 | 1.10649 | 0.01880 | 0.12105 | 0.00144 | 811 | 17 | 756 | 9 | 737 | 8 |
| ER13-1-29 | 0.06615 | 0.00104 | 1.10343 | 0.01771 | 0.12091 | 0.00119 | 811 | 18 | 755 | 9 | 736 | 7 |
| 12ER7-1-01 | 0.06579 | 0.00149 | 1.11043 | 0.02513 | 0.12111 | 0.00113 | 800 | 32 | 758 | 12 | 737 | 7 |
| 12ER7-1-02 | 0.06268 | 0.00141 | 1.14232 | 0.02626 | 0.13070 | 0.00128 | 697 | 32 | 774 | 12 | 792 | 7 |
| 12ER7-1-03 | 0.06432 | 0.00215 | 1.11088 | 0.03580 | 0.12527 | 0.00110 | 752 | 72 | 759 | 17 | 761 | 6 |
| 12ER7-1-04 | 0.06335 | 0.00148 | 1.11128 | 0.02602 | 0.12555 | 0.00101 | 720 | 36 | 759 | 13 | 762 | 6 |
| 12ER7-1-05 | 0.06480 | 0.00172 | 1.09510 | 0.02874 | 0.12115 | 0.00118 | 768 | 39 | 751 | 14 | 737 | 7 |
| 12ER7-1-07 | 0.06377 | 0.00156 | 1.11932 | 0.02620 | 0.12560 | 0.00106 | 734 | 35 | 763 | 13 | 763 | 6 |
| 12ER7-1-08 | 0.06658 | 0.00158 | 1.17020 | 0.02686 | 0.12543 | 0.00102 | 825 | 34 | 787 | 13 | 762 | 6 |
| 12ER7-1-11 | 0.06116 | 0.00178 | 1.08134 | 0.03046 | 0.12550 | 0.00129 | 645 | 43 | 744 | 15 | 762 | 7 |
| 12ER7-1-12 | 0.07204 | 0.00211 | 1.22563 | 0.03479 | 0.12121 | 0.00119 | 987 | 42 | 812 | 16 | 738 | 7 |
| 12ER7-1-14 | 0.06122 | 0.00157 | 1.12350 | 0.02778 | 0.13075 | 0.00117 | 647 | 38 | 765 | 13 | 792 | 7 |
| 12ER7-1-15 | 0.06353 | 0.00173 | 1.16622 | 0.03105 | 0.13085 | 0.00140 | 726 | 38 | 785 | 15 | 793 | 8 |
| 12ER7-1-16 | 0.06408 | 0.00170 | 1.12937 | 0.02933 | 0.12580 | 0.00143 | 744 | 36 | 767 | 14 | 764 | 8 |
| 12ER7-1-17 | 0.06392 | 0.00302 | 1.09605 | 0.05006 | 0.12437 | 0.00148 | 739 | 102 | 751 | 24 | 756 | 8 |
| 12ER7-1-18 | 0.06668 | 0.00149 | 1.13363 | 0.02509 | 0.12117 | 0.00111 | 828 | 31 | 769 | 12 | 737 | 6 |
| 12ER7-1-20 | 0.06483 | 0.00174 | 1.18855 | 0.03373 | 0.13065 | 0.00179 | 769 | 37 | 795 | 16 | 792 | 10 |
| 12ER7-1-21 | 0.06276 | 0.00163 | 1.14736 | 0.02949 | 0.13075 | 0.00142 | 700 | 36 | 776 | 14 | 792 | 8 |
| 12ER7-1-23 | 0.06444 | 0.00141 | 1.17461 | 0.02571 | 0.13079 | 0.00117 | 756 | 31 | 789 | 12 | 792 | 7 |
| 12ER7-1-24 | 0.06326 | 0.00164 | 1.14597 | 0.02856 | 0.13082 | 0.00136 | 717 | 35 | 775 | 14 | 793 | 8 |
| 12ER7-1-25 | 0.06608 | 0.00314 | 1.14148 | 0.05260 | 0.12529 | 0.00148 | 809 | 102 | 773 | 25 | 761 | 8 |
| 12ER7-1-27 | 0.06453 | 0.00162 | 1.16804 | 0.02983 | 0.13069 | 0.00135 | 759 | 36 | 786 | 14 | 792 | 8 |
| 12ER7-1-28 | 0.06209 | 0.00144 | 1.12528 | 0.02628 | 0.13074 | 0.00118 | 677 | 34 | 766 | 13 | 792 | 7 |
| 11ER24-1-01 | 0.06271 | 0.00174 | 1.22287 | 0.03337 | 0.14086 | 0.00167 | 698 | 38 | 811 | 15 | 850 | 9 |
| 11ER24-1-02 | 0.06542 | 0.00149 | 1.18591 | 0.02742 | 0.13054 | 0.00129 | 788 | 32 | 794 | 13 | 791 | 7 |
| 11ER24-1-03 | 0.06616 | 0.00150 | 1.15765 | 0.02680 | 0.12598 | 0.00124 | 812 | 32 | 781 | 13 | 765 | 7 |
| 11ER24-1-04 | 0.06842 | 0.00149 | 1.18645 | 0.02563 | 0.12499 | 0.00122 | 881 | 29 | 794 | 12 | 759 | 7 |
| 11ER24-1-05 | 0.06784 | 0.00181 | 1.22759 | 0.03369 | 0.13038 | 0.00147 | 864 | 38 | 813 | 15 | 790 | 8 |
| 11ER24-1-06 | 0.07412 | 0.00201 | 1.45203 | 0.04166 | 0.14100 | 0.00174 | 1045 | 38 | 911 | 17 | 850 | 10 |
| 11ER24-1-07 | 0.06344 | 0.00287 | 1.09512 | 0.04667 | 0.12520 | 0.00189 | 723 | 98 | 751 | 23 | 760 | 11 |
| 11ER24-1-08 | 0.06725 | 0.00173 | 1.21556 | 0.03078 | 0.13046 | 0.00141 | 846 | 35 | 808 | 14 | 790 | 8 |
| 11ER24-1-09 | 0.06685 | 0.00171 | 1.20746 | 0.03097 | 0.13046 | 0.00148 | 833 | 35 | 804 | 14 | 790 | 8 |
| 11ER24-1-10 | 0.06575 | 0.00154 | 1.18960 | 0.02829 | 0.13047 | 0.00128 | 799 | 33 | 796 | 13 | 791 | 7 |
| 11ER24-1-11 | 0.06672 | 0.00149 | 1.30313 | 0.02904 | 0.14097 | 0.00137 | 829 | 30 | 847 | 13 | 850 | 8 |
| 11ER24-1-12 | 0.06945 | 0.00152 | 1.20731 | 0.02795 | 0.12557 | 0.00159 | 912 | 27 | 804 | 13 | 763 | 9 |
| 11ER24-1-13 | 0.07107 | 0.00170 | 1.23345 | 0.03108 | 0.12564 | 0.00181 | 960 | 29 | 816 | 14 | 763 | 10 |
| 11ER24-1-14 | 0.07799 | 0.00340 | 1.34375 | 0.05627 | 0.12496 | 0.00152 | 1147 | 89 | 865 | 24 | 759 | 9 |
| 11ER24-1-15 | 0.06705 | 0.00174 | 1.31073 | 0.03654 | 0.14102 | 0.00189 | 839 | 36 | 850 | 16 | 850 | 11 |
| 11ER24-1-16 | 0.06696 | 0.00165 | 1.30662 | 0.03356 | 0.14092 | 0.00170 | 837 | 33 | 849 | 15 | 850 | 10 |
| 11ER24-1-17 | 0.06656 | 0.00163 | 1.29991 | 0.03525 | 0.14116 | 0.00214 | 824 | 32 | 846 | 16 | 851 | 12 |
| 11ER24-1-18 | 0.06553 | 0.00186 | 1.13838 | 0.03524 | 0.12530 | 0.00167 | 791 | 43 | 772 | 17 | 761 | 10 |
| 11ER24-1-19 | 0.06468 | 0.00132 | 1.16817 | 0.02545 | 0.13037 | 0.00138 | 764 | 28 | 786 | 12 | 790 | 8 |
| 11ER24-1-20 | 0.06569 | 0.00144 | 1.19150 | 0.02922 | 0.13085 | 0.00164 | 796 | 31 | 797 | 14 | 793 | 9 |
| 14ER17-1-01 | 0.07171 | 0.00131 | 1.48704 | 0.02908 | 0.14925 | 0.00144 | 978 | 24 | 925 | 12 | 897 | 8 |
| 14ER17-1-02 | 0.06615 | 0.00111 | 1.15708 | 0.02152 | 0.12588 | 0.00122 | 811 | 23 | 781 | 10 | 764 | 7 |
| 14ER17-1-03 | 0.06477 | 0.00122 | 1.01913 | 0.01914 | 0.11329 | 0.00066 | 767 | 30 | 713 | 10 | 692 | 4 |
| 14ER17-1-04 | 0.06569 | 0.00130 | 1.19366 | 0.02628 | 0.13058 | 0.00133 | 797 | 29 | 798 | 12 | 791 | 8 |
| 14ER17-1-05 | 0.06664 | 0.00140 | 1.20871 | 0.02588 | 0.13062 | 0.00115 | 827 | 30 | 805 | 12 | 791 | 7 |
| 14ER17-1-06 | 0.06505 | 0.00139 | 0.99397 | 0.02251 | 0.10979 | 0.00103 | 776 | 32 | 701 | 11 | 672 | 6 |
| 14ER17-1-07 | 0.06661 | 0.00139 | 1.03156 | 0.02089 | 0.11151 | 0.00078 | 825 | 31 | 720 | 10 | 682 | 5 |
| 14ER17-1-08 | 0.06523 | 0.00117 | 1.18729 | 0.02134 | 0.13099 | 0.00108 | 782 | 24 | 795 | 10 | 794 | 6 |
| 14ER17-1-09 | 0.06621 | 0.00107 | 1.20274 | 0.02228 | 0.13046 | 0.00144 | 813 | 21 | 802 | 10 | 790 | 8 |
| 14ER17-1-10 | 0.06728 | 0.00120 | 1.07911 | 0.01945 | 0.11514 | 0.00088 | 846 | 25 | 743 | 9 | 703 | 5 |
| 14ER17-1-11 | 0.06345 | 0.00124 | 1.15721 | 0.02293 | 0.13094 | 0.00116 | 723 | 27 | 781 | 11 | 793 | 7 |
| 14ER17-1-12 | 0.06457 | 0.00128 | 1.16342 | 0.02311 | 0.12936 | 0.00108 | 760 | 28 | 784 | 11 | 784 | 6 |
| 14ER17-1-13 | 0.06366 | 0.00112 | 1.15647 | 0.02249 | 0.13058 | 0.00150 | 730 | 23 | 780 | 11 | 791 | 9 |
| 14ER17-1-14 | 0.06380 | 0.00108 | 1.15655 | 0.02018 | 0.13056 | 0.00131 | 735 | 21 | 780 | 10 | 791 | 7 |
| ER24-1-01 | 0.06759 | 0.00182 | 1.22339 | 0.03212 | 0.13079 | 0.00144 | 856 | 36 | 811 | 15 | 792 | 8 |
| ER24-1-02 | 0.06445 | 0.00144 | 1.16896 | 0.02744 | 0.13088 | 0.00139 | 757 | 32 | 786 | 13 | 793 | 8 |
| ER24-1-03 | 0.06554 | 0.00168 | 1.18415 | 0.02895 | 0.13083 | 0.00131 | 792 | 34 | 793 | 13 | 793 | 7 |
| ER24-1-04 | 0.06583 | 0.00124 | 1.26397 | 0.02430 | 0.13859 | 0.00113 | 801 | 27 | 830 | 11 | 837 | 6 |
| ER24-1-05 | 0.06788 | 0.00116 | 1.30521 | 0.02507 | 0.13864 | 0.00138 | 865 | 24 | 848 | 11 | 837 | 8 |
| ER24-1-06 | 0.06613 | 0.00137 | 1.26906 | 0.02609 | 0.13863 | 0.00118 | 810 | 29 | 832 | 12 | 837 | 7 |
| ER24-1-07 | 0.06759 | 0.00146 | 1.22802 | 0.02618 | 0.13110 | 0.00115 | 856 | 30 | 813 | 12 | 794 | 7 |
| ER24-1-08 | 0.06743 | 0.00133 | 1.21809 | 0.02302 | 0.13074 | 0.00121 | 851 | 24 | 809 | 11 | 792 | 7 |
| ER24-1-09 | 0.06552 | 0.00102 | 1.25841 | 0.02146 | 0.13845 | 0.00124 | 791 | 21 | 827 | 10 | 836 | 7 |
| ER24-1-10 | 0.06810 | 0.00118 | 1.23644 | 0.02377 | 0.13074 | 0.00115 | 872 | 25 | 817 | 11 | 792 | 7 |
| ER24-1-11 | 0.06489 | 0.00142 | 1.17673 | 0.02437 | 0.13078 | 0.00105 | 771 | 30 | 790 | 11 | 792 | 6 |
| ER24-1-12 | 0.06539 | 0.00143 | 1.18648 | 0.02584 | 0.13087 | 0.00121 | 787 | 30 | 794 | 12 | 793 | 7 |
| ER24-1-13 | 0.06461 | 0.00160 | 1.17219 | 0.02955 | 0.13085 | 0.00132 | 762 | 36 | 788 | 14 | 793 | 8 |
| ER24-1-14 | 0.06598 | 0.00126 | 1.26496 | 0.02592 | 0.13847 | 0.00151 | 806 | 25 | 830 | 12 | 836 | 9 |
| ER24-1-15 | 0.06625 | 0.00123 | 1.27217 | 0.02397 | 0.13870 | 0.00118 | 814 | 25 | 833 | 11 | 837 | 7 |
| ER24-1-17 | 0.06656 | 0.00114 | 1.20428 | 0.02135 | 0.13080 | 0.00102 | 824 | 24 | 803 | 10 | 792 | 6 |
| ER24-1-20 | 0.06748 | 0.00122 | 1.21877 | 0.02299 | 0.13070 | 0.00098 | 853 | 27 | 809 | 11 | 792 | 6 |
| ER24-1-21 | 0.06674 | 0.00124 | 1.20667 | 0.02385 | 0.13089 | 0.00122 | 830 | 26 | 804 | 11 | 793 | 7 |
| ER24-1-22 | 0.06701 | 0.00115 | 1.28416 | 0.02297 | 0.13875 | 0.00102 | 838 | 25 | 839 | 10 | 838 | 6 |
| ER24-1-23 | 0.06571 | 0.00144 | 1.18595 | 0.02715 | 0.13062 | 0.00119 | 797 | 33 | 794 | 13 | 791 | 7 |
| ER24-1-24 | 0.06752 | 0.00113 | 1.21898 | 0.02215 | 0.13064 | 0.00105 | 854 | 24 | 809 | 10 | 792 | 6 |
| 11ER23-1-01 | 0.06860 | 0.00200 | 1.33930 | 0.03770 | 0.14050 | 0.00140 | 888 | 42 | 863 | 16 | 847 | 8 |
| 11ER23-1-02 | 0.06570 | 0.00150 | 1.29030 | 0.02990 | 0.14120 | 0.00140 | 797 | 32 | 841 | 13 | 851 | 8 |
| 11ER23-1-03 | 0.06580 | 0.00170 | 1.19490 | 0.03090 | 0.13050 | 0.00130 | 801 | 38 | 798 | 14 | 791 | 7 |
| 11ER23-1-04 | 0.06330 | 0.00170 | 1.22310 | 0.03060 | 0.14110 | 0.00260 | 717 | 25 | 811 | 14 | 851 | 15 |
| 11ER23-1-05 | 0.06600 | 0.00190 | 1.29180 | 0.03720 | 0.14090 | 0.00130 | 807 | 45 | 842 | 16 | 850 | 7 |
| 11ER23-1-06 | 0.06560 | 0.00190 | 1.18760 | 0.03340 | 0.13070 | 0.00130 | 794 | 42 | 795 | 15 | 792 | 8 |
| 11ER23-1-07 | 0.06450 | 0.00250 | 1.25890 | 0.04830 | 0.14090 | 0.00160 | 759 | 61 | 827 | 22 | 850 | 9 |
| 11ER23-1-08 | 0.06850 | 0.00180 | 1.41510 | 0.03840 | 0.14820 | 0.00180 | 883 | 36 | 895 | 16 | 891 | 10 |
| 11ER23-1-09 | 0.06470 | 0.00180 | 1.26490 | 0.03490 | 0.14090 | 0.00140 | 763 | 41 | 830 | 16 | 849 | 8 |
| 11ER23-1-10 | 0.06477 | 0.00149 | 1.27228 | 0.03103 | 0.14087 | 0.00138 | 767 | 35 | 833 | 14 | 850 | 8 |
| 11ER23-1-11 | 0.06500 | 0.00160 | 1.27180 | 0.03010 | 0.14100 | 0.00130 | 774 | 35 | 833 | 13 | 850 | 7 |
| 11ER23-1-12 | 0.06530 | 0.00150 | 1.18850 | 0.02810 | 0.13070 | 0.00140 | 785 | 32 | 795 | 13 | 792 | 8 |
| 11ER23-1-13 | 0.06530 | 0.00170 | 1.27650 | 0.03160 | 0.14090 | 0.00140 | 785 | 35 | 835 | 14 | 850 | 8 |
| 11ER23-1-14 | 0.06460 | 0.00160 | 1.26230 | 0.03130 | 0.14060 | 0.00140 | 760 | 36 | 829 | 14 | 848 | 8 |
| 11ER23-1-15 | 0.06310 | 0.00140 | 1.23600 | 0.02910 | 0.14090 | 0.00150 | 712 | 32 | 817 | 13 | 849 | 8 |
| 11ER23-1-16 | 0.06500 | 0.00190 | 1.17760 | 0.03450 | 0.13070 | 0.00160 | 775 | 41 | 790 | 16 | 792 | 9 |
| 11ER23-1-18 | 0.06270 | 0.00160 | 1.13490 | 0.03040 | 0.13090 | 0.00230 | 699 | 30 | 770 | 14 | 793 | 13 |
| 11ER23-1-19 | 0.06660 | 0.00290 | 1.20250 | 0.05230 | 0.13090 | 0.00200 | 826 | 65 | 802 | 24 | 793 | 11 |
| 11ER23-1-20 | 0.06580 | 0.00150 | 1.28770 | 0.02920 | 0.14100 | 0.00130 | 799 | 32 | 840 | 13 | 850 | 8 |
| 14ER13-1-01 | 0.06575 | 0.00135 | 1.18873 | 0.02809 | 0.13120 | 0.00180 | 799 | 27 | 795 | 13 | 795 | 10 |
| 14ER13-1-02 | 0.06531 | 0.00124 | 1.16880 | 0.02664 | 0.12960 | 0.00150 | 784 | 29 | 786 | 12 | 786 | 9 |
| 14ER13-1-03 | 0.06765 | 0.00127 | 1.22420 | 0.02464 | 0.13120 | 0.00130 | 858 | 26 | 812 | 11 | 795 | 7 |
| 14ER13-1-04 | 0.06599 | 0.00121 | 1.19049 | 0.02567 | 0.13058 | 0.00145 | 806 | 27 | 796 | 12 | 791 | 8 |
| 14ER13-1-05 | 0.06568 | 0.00131 | 1.18382 | 0.02416 | 0.13057 | 0.00104 | 796 | 29 | 793 | 11 | 791 | 6 |
| 14ER13-1-06 | 0.06569 | 0.00136 | 1.19127 | 0.03064 | 0.13118 | 0.00209 | 797 | 29 | 797 | 14 | 795 | 12 |
| 14ER13-1-07 | 0.06478 | 0.00133 | 1.17368 | 0.02818 | 0.13133 | 0.00196 | 767 | 27 | 788 | 13 | 795 | 11 |
| 14ER13-1-08 | 0.06438 | 0.00125 | 1.15863 | 0.02309 | 0.13048 | 0.00108 | 754 | 28 | 781 | 11 | 791 | 6 |
| 14ER13-1-09 | 0.06317 | 0.00168 | 1.14005 | 0.03354 | 0.13085 | 0.00160 | 714 | 42 | 773 | 16 | 793 | 9 |
| 14ER13-1-10 | 0.06094 | 0.00127 | 1.10406 | 0.02817 | 0.13107 | 0.00202 | 637 | 30 | 755 | 14 | 794 | 12 |
| 14ER13-1-11 | 0.06239 | 0.00126 | 1.13081 | 0.02484 | 0.13127 | 0.00133 | 688 | 30 | 768 | 12 | 795 | 8 |
| 14ER13-1-12 | 0.06281 | 0.00126 | 1.13798 | 0.02419 | 0.13126 | 0.00145 | 702 | 27 | 772 | 11 | 795 | 8 |
| 14ER13-1-13 | 0.06338 | 0.00121 | 1.14968 | 0.02423 | 0.13105 | 0.00126 | 721 | 28 | 777 | 11 | 794 | 7 |
| 13ER41-1-01 | 0.06663 | 0.00098 | 1.20906 | 0.01731 | 0.13089 | 0.00083 | 826 | 19 | 805 | 8 | 793 | 5 |
| 13ER41-1-02 | 0.06629 | 0.00094 | 1.20174 | 0.01863 | 0.13073 | 0.00122 | 816 | 18 | 801 | 9 | 792 | 7 |
| 13ER41-1-03 | 0.06596 | 0.00094 | 1.26042 | 0.01842 | 0.13786 | 0.00113 | 805 | 17 | 828 | 8 | 833 | 6 |
| 13ER41-1-04 | 0.06629 | 0.00098 | 1.25873 | 0.01821 | 0.13682 | 0.00079 | 815 | 21 | 827 | 8 | 827 | 4 |
| 13ER41-1-05 | 0.06519 | 0.00103 | 1.18645 | 0.01851 | 0.13116 | 0.00088 | 781 | 21 | 794 | 9 | 794 | 5 |
| 13ER41-1-06 | 0.06590 | 0.00117 | 1.26145 | 0.02242 | 0.13792 | 0.00106 | 803 | 24 | 829 | 10 | 833 | 6 |
| 13ER41-1-07 | 0.06670 | 0.00112 | 1.21348 | 0.02023 | 0.13112 | 0.00097 | 828 | 22 | 807 | 9 | 794 | 6 |
| 13ER41-1-13 | 0.06616 | 0.00089 | 1.19896 | 0.01729 | 0.13083 | 0.00109 | 812 | 17 | 800 | 8 | 793 | 6 |
| 13ER41-1-14 | 0.06926 | 0.00096 | 1.25485 | 0.01777 | 0.13093 | 0.00103 | 907 | 17 | 826 | 8 | 793 | 6 |
| 13ER41-1-15 | 0.06551 | 0.00088 | 1.19715 | 0.01776 | 0.13211 | 0.00126 | 791 | 16 | 799 | 8 | 800 | 7 |
| 13ER41-1-16 | 0.06882 | 0.00139 | 1.33825 | 0.02606 | 0.14082 | 0.00096 | 893 | 29 | 862 | 11 | 849 | 5 |
| 13ER41-1-17 | 0.06565 | 0.00093 | 1.18814 | 0.01719 | 0.13089 | 0.00096 | 795 | 18 | 795 | 8 | 793 | 5 |
| 13ER41-1-18 | 0.06905 | 0.00085 | 1.25352 | 0.01602 | 0.13114 | 0.00078 | 900 | 17 | 825 | 7 | 794 | 4 |
| 13ER43-1-01 | 0.06516 | 0.00120 | 1.18632 | 0.02204 | 0.13163 | 0.00104 | 780 | 26 | 794 | 10 | 797 | 6 |
| 13ER43-1-02 | 0.06671 | 0.00122 | 1.27478 | 0.02285 | 0.13814 | 0.00086 | 829 | 27 | 835 | 10 | 834 | 5 |
| 13ER43-1-04 | 0.06553 | 0.00140 | 1.18570 | 0.02515 | 0.13086 | 0.00109 | 792 | 30 | 794 | 12 | 793 | 6 |
| 13ER43-1-05 | 0.06521 | 0.00127 | 1.24738 | 0.02530 | 0.13803 | 0.00106 | 781 | 30 | 822 | 11 | 833 | 6 |
| 13ER43-1-06 | 0.06636 | 0.00124 | 1.20414 | 0.02245 | 0.13108 | 0.00101 | 818 | 26 | 802 | 10 | 794 | 6 |
| 13ER43-1-08 | 0.06744 | 0.00143 | 1.47341 | 0.03124 | 0.15762 | 0.00126 | 851 | 31 | 920 | 13 | 944 | 7 |
| 13ER43-1-09 | 0.06545 | 0.00106 | 1.25786 | 0.02054 | 0.13857 | 0.00094 | 789 | 23 | 827 | 9 | 837 | 5 |
| 13ER43-1-10 | 0.06676 | 0.00114 | 1.21225 | 0.02139 | 0.13093 | 0.00108 | 830 | 23 | 806 | 10 | 793 | 6 |
| 13ER43-1-11 | 0.06352 | 0.00146 | 1.20929 | 0.02604 | 0.13785 | 0.00110 | 726 | 32 | 805 | 12 | 833 | 6 |
| 13ER43-1-12 | 0.06495 | 0.00112 | 1.18374 | 0.01951 | 0.13146 | 0.00105 | 773 | 21 | 793 | 9 | 796 | 6 |
| 13ER43-1-13 | 0.06538 | 0.00128 | 1.18990 | 0.02277 | 0.13111 | 0.00108 | 787 | 26 | 796 | 11 | 794 | 6 |
| 13ER43-1-14 | 0.06588 | 0.00138 | 1.19697 | 0.02412 | 0.13116 | 0.00116 | 802 | 27 | 799 | 11 | 794 | 7 |
| 13ER43-1-15 | 0.06369 | 0.00122 | 1.15850 | 0.02190 | 0.13115 | 0.00105 | 731 | 26 | 781 | 10 | 794 | 6 |
| 13ER43-1-16 | 0.06352 | 0.00121 | 1.15146 | 0.02260 | 0.13093 | 0.00142 | 726 | 24 | 778 | 11 | 793 | 8 |
| 13ER43-1-17 | 0.06601 | 0.00125 | 1.19870 | 0.02259 | 0.13105 | 0.00098 | 807 | 27 | 800 | 10 | 794 | 6 |
| 13ER43-1-19 | 0.06604 | 0.00107 | 1.20083 | 0.02079 | 0.13089 | 0.00098 | 808 | 24 | 801 | 10 | 793 | 6 |
| 13ER44-1-01 | 0.06599 | 0.00102 | 1.20316 | 0.01986 | 0.13142 | 0.00098 | 806 | 22 | 802 | 9 | 796 | 6 |
| 13ER44-1-02 | 0.06405 | 0.00151 | 1.22437 | 0.02815 | 0.13847 | 0.00111 | 743 | 35 | 812 | 13 | 836 | 6 |
| 13ER44-1-03 | 0.06552 | 0.00110 | 1.25416 | 0.02154 | 0.13828 | 0.00103 | 791 | 24 | 825 | 10 | 835 | 6 |
| 13ER44-1-04 | 0.06419 | 0.00121 | 1.16433 | 0.02241 | 0.13122 | 0.00117 | 748 | 26 | 784 | 11 | 795 | 7 |
| 13ER44-1-05 | 0.06854 | 0.00179 | 1.24571 | 0.03760 | 0.13081 | 0.00150 | 885 | 43 | 821 | 17 | 792 | 9 |
| 13ER44-1-06 | 0.06418 | 0.00114 | 1.16271 | 0.02131 | 0.13092 | 0.00105 | 748 | 25 | 783 | 10 | 793 | 6 |
| 13ER44-1-07 | 0.06885 | 0.00153 | 1.31664 | 0.02906 | 0.13826 | 0.00102 | 894 | 33 | 853 | 13 | 835 | 6 |
| 13ER44-1-08 | 0.06584 | 0.00127 | 1.25554 | 0.02367 | 0.13804 | 0.00098 | 801 | 28 | 826 | 11 | 834 | 6 |
| 13ER44-1-09 | 0.06469 | 0.00146 | 1.17251 | 0.02700 | 0.13111 | 0.00109 | 764 | 34 | 788 | 13 | 794 | 6 |
| 13ER44-1-10 | 0.06809 | 0.00147 | 1.23153 | 0.02571 | 0.13096 | 0.00101 | 871 | 30 | 815 | 12 | 793 | 6 |
| 13ER44-1-11 | 0.06639 | 0.00151 | 1.27088 | 0.02903 | 0.13837 | 0.00118 | 819 | 33 | 833 | 13 | 835 | 7 |
| 13ER44-1-12 | 0.06555 | 0.00123 | 1.25417 | 0.02334 | 0.13836 | 0.00108 | 792 | 26 | 825 | 11 | 835 | 6 |
| 13ER44-1-13 | 0.06431 | 0.00111 | 1.23379 | 0.02157 | 0.13850 | 0.00093 | 752 | 25 | 816 | 10 | 836 | 5 |
| 13ER44-1-14 | 0.06599 | 0.00116 | 1.19576 | 0.02091 | 0.13098 | 0.00100 | 806 | 24 | 799 | 10 | 793 | 6 |
| 13ER44-1-15 | 0.06623 | 0.00134 | 1.20647 | 0.02439 | 0.13149 | 0.00097 | 814 | 30 | 804 | 11 | 796 | 6 |
| 13ER44-1-16 | 0.06490 | 0.00155 | 1.24375 | 0.03000 | 0.13856 | 0.00119 | 771 | 36 | 821 | 14 | 837 | 7 |
| 13ER44-1-17 | 0.06648 | 0.00136 | 1.26172 | 0.02533 | 0.13706 | 0.00096 | 822 | 30 | 829 | 11 | 828 | 5 |
| 13ER44-1-18 | 0.06744 | 0.00120 | 1.29408 | 0.02248 | 0.13861 | 0.00091 | 851 | 25 | 843 | 10 | 837 | 5 |
| 13ER44-1-19 | 0.06475 | 0.00124 | 1.24204 | 0.02428 | 0.13865 | 0.00115 | 766 | 27 | 820 | 11 | 837 | 7 |
| 13ER44-1-20 | 0.06620 | 0.00127 | 1.27078 | 0.02479 | 0.13880 | 0.00120 | 813 | 26 | 833 | 11 | 838 | 7 |
| 14ER11-1-01 | 0.06505 | 0.00113 | 1.18932 | 0.02158 | 0.13131 | 0.00132 | 776 | 22 | 796 | 10 | 795 | 8 |
| 14ER11-1-02 | 0.06449 | 0.00116 | 1.18174 | 0.02123 | 0.13153 | 0.00103 | 758 | 25 | 792 | 10 | 797 | 6 |
| 14ER11-1-03 | 0.06548 | 0.00150 | 1.20249 | 0.02778 | 0.13196 | 0.00121 | 790 | 33 | 802 | 13 | 799 | 7 |
| 14ER11-1-04 | 0.06338 | 0.00133 | 1.15574 | 0.02363 | 0.13103 | 0.00113 | 721 | 29 | 780 | 11 | 794 | 6 |
| 14ER11-1-05 | 0.06813 | 0.00152 | 1.39515 | 0.02845 | 0.14747 | 0.00132 | 872 | 27 | 887 | 12 | 887 | 7 |
| 14ER11-1-06 | 0.06539 | 0.00125 | 1.19260 | 0.02591 | 0.13077 | 0.00180 | 787 | 24 | 797 | 12 | 792 | 10 |
| 14ER11-1-07 | 0.06500 | 0.00158 | 1.18011 | 0.02775 | 0.13049 | 0.00118 | 774 | 34 | 791 | 13 | 791 | 7 |
| 14ER11-1-08 | 0.06501 | 0.00117 | 1.19048 | 0.02207 | 0.13136 | 0.00124 | 775 | 23 | 796 | 10 | 796 | 7 |
| 14ER11-1-09 | 0.06640 | 0.00141 | 1.20795 | 0.02571 | 0.13054 | 0.00106 | 819 | 31 | 804 | 12 | 791 | 6 |
| 14ER11-1-10 | 0.06423 | 0.00142 | 1.18055 | 0.02685 | 0.13197 | 0.00116 | 749 | 33 | 792 | 13 | 799 | 7 |
| 14ER11-1-11 | 0.06520 | 0.00117 | 1.19366 | 0.02280 | 0.13140 | 0.00112 | 781 | 26 | 798 | 11 | 796 | 6 |
| 14ER11-1-12 | 0.06397 | 0.00173 | 1.15934 | 0.03114 | 0.13059 | 0.00131 | 741 | 40 | 782 | 15 | 791 | 7 |
| 14ER11-1-13 | 0.06574 | 0.00152 | 1.19038 | 0.02907 | 0.13049 | 0.00134 | 798 | 34 | 796 | 13 | 791 | 8 |
| 14ER11-1-14 | 0.06246 | 0.00142 | 1.14574 | 0.02943 | 0.13200 | 0.00162 | 690 | 34 | 775 | 14 | 799 | 9 |
| 14ER11-1-15 | 0.06400 | 0.00169 | 1.15963 | 0.03291 | 0.13083 | 0.00159 | 742 | 39 | 782 | 15 | 793 | 9 |
| 13ER12-1-01 | 0.06700 | 0.00085 | 1.30391 | 0.01667 | 0.14028 | 0.00071 | 838 | 18 | 847 | 7 | 846 | 4 |
| 13ER12-1-02 | 0.06720 | 0.00082 | 1.31053 | 0.01752 | 0.14060 | 0.00099 | 844 | 16 | 850 | 8 | 848 | 6 |
| 13ER12-1-03 | 0.06722 | 0.00092 | 1.30445 | 0.01881 | 0.13999 | 0.00099 | 845 | 18 | 848 | 8 | 845 | 6 |
| 13ER12-1-04 | 0.06739 | 0.00096 | 1.38202 | 0.02098 | 0.14786 | 0.00100 | 850 | 20 | 881 | 9 | 889 | 6 |
| 13ER12-1-05 | 0.06883 | 0.00118 | 1.41338 | 0.02513 | 0.14849 | 0.00134 | 894 | 22 | 895 | 11 | 892 | 8 |
| 13ER12-1-06 | 0.06871 | 0.00106 | 1.41242 | 0.02260 | 0.14843 | 0.00117 | 890 | 20 | 894 | 10 | 892 | 7 |
| 13ER12-1-07 | 0.06741 | 0.00105 | 1.31177 | 0.02098 | 0.14045 | 0.00101 | 850 | 21 | 851 | 9 | 847 | 6 |
| 13ER12-1-08 | 0.06817 | 0.00089 | 1.29881 | 0.01805 | 0.13741 | 0.00093 | 874 | 18 | 845 | 8 | 830 | 5 |
| 13ER12-1-09 | 0.06802 | 0.00085 | 1.36136 | 0.01825 | 0.14430 | 0.00100 | 869 | 17 | 872 | 8 | 869 | 6 |
| 13ER12-1-10 | 0.06804 | 0.00099 | 1.36078 | 0.02081 | 0.14414 | 0.00110 | 870 | 19 | 872 | 9 | 868 | 6 |
| 13ER12-1-11 | 0.06836 | 0.00103 | 1.36032 | 0.02125 | 0.14327 | 0.00103 | 880 | 20 | 872 | 9 | 863 | 6 |
| 13ER12-1-12 | 0.06807 | 0.00106 | 1.35184 | 0.02061 | 0.14330 | 0.00106 | 871 | 19 | 868 | 9 | 863 | 6 |
| 13ER12-1-13 | 0.06877 | 0.00100 | 1.41393 | 0.02084 | 0.14808 | 0.00097 | 892 | 20 | 895 | 9 | 890 | 5 |
| 13ER12-1-14 | 0.06771 | 0.00083 | 1.39385 | 0.01919 | 0.14812 | 0.00117 | 860 | 16 | 886 | 8 | 890 | 7 |
| 13ER12-1-15 | 0.06847 | 0.00083 | 1.28215 | 0.01582 | 0.13473 | 0.00080 | 883 | 16 | 838 | 7 | 815 | 5 |
| 13ER12-1-16 | 0.06765 | 0.00082 | 1.27304 | 0.01581 | 0.13543 | 0.00092 | 858 | 15 | 834 | 7 | 819 | 5 |
| 13ER13-1-01 | 0.06856 | 0.00110 | 1.38852 | 0.01981 | 0.14688 | 0.00106 | 886 | 34 | 884 | 8 | 883 | 6 |
| 13ER13-1-02 | 0.06827 | 0.00081 | 1.33165 | 0.01669 | 0.14043 | 0.00093 | 877 | 15 | 860 | 7 | 847 | 5 |
| 13ER13-1-03 | 0.06942 | 0.00092 | 1.46937 | 0.01959 | 0.15285 | 0.00123 | 911 | 15 | 918 | 8 | 917 | 7 |
| 13ER13-1-04 | 0.06762 | 0.00090 | 1.32204 | 0.01839 | 0.14079 | 0.00094 | 857 | 18 | 855 | 8 | 849 | 5 |
| 13ER13-1-05 | 0.06852 | 0.00100 | 1.33620 | 0.02018 | 0.14074 | 0.00114 | 884 | 18 | 862 | 9 | 849 | 6 |
| 13ER13-1-06 | 0.06969 | 0.00092 | 1.48138 | 0.02309 | 0.15326 | 0.00146 | 919 | 17 | 923 | 9 | 919 | 8 |
| 13ER13-1-07 | 0.07069 | 0.00134 | 1.35817 | 0.02398 | 0.13934 | 0.00097 | 948 | 40 | 871 | 10 | 841 | 5 |
| 13ER13-1-08 | 0.06823 | 0.00152 | 1.32074 | 0.02717 | 0.14040 | 0.00121 | 875 | 47 | 855 | 12 | 847 | 7 |
| 13ER13-1-09 | 0.06872 | 0.00145 | 1.39389 | 0.02722 | 0.14710 | 0.00117 | 890 | 45 | 886 | 12 | 885 | 7 |
| 13ER13-1-10 | 0.07447 | 0.00113 | 1.44563 | 0.02287 | 0.14029 | 0.00119 | 1054 | 19 | 908 | 9 | 846 | 7 |
| 13ER13-1-11 | 0.06855 | 0.00920 | 1.39493 | 0.02021 | 0.14682 | 0.00115 | 885 | 17 | 887 | 9 | 883 | 6 |
| 13ER13-1-12 | 0.06986 | 0.00088 | 1.41375 | 0.02021 | 0.14601 | 0.00126 | 924 | 16 | 895 | 9 | 879 | 7 |
| 13ER13-1-13 | 0.07039 | 0.00086 | 1.43318 | 0.01765 | 0.14685 | 0.00082 | 940 | 16 | 903 | 7 | 883 | 5 |
| 13ER13-1-14 | 0.07028 | 0.00085 | 1.42972 | 0.01982 | 0.14669 | 0.00127 | 936 | 15 | 901 | 8 | 882 | 7 |
| 13ER13-1-15 | 0.07109 | 0.00087 | 1.51062 | 0.02098 | 0.15301 | 0.00119 | 960 | 16 | 935 | 8 | 918 | 7 |
| 12ER28-1-01 | 0.06950 | 0.00150 | 1.11262 | 0.02374 | 0.11485 | 0.00100 | 914 | 30 | 759 | 11 | 701 | 6 |
| 12ER28-1-02 | 0.06955 | 0.00142 | 1.11150 | 0.02319 | 0.11468 | 0.00119 | 915 | 26 | 759 | 11 | 700 | 7 |
| 12ER28-1-03 | 0.06760 | 0.00145 | 1.33486 | 0.03382 | 0.14146 | 0.00220 | 856 | 28 | 861 | 15 | 853 | 12 |
| 12ER28-1-04 | 0.06794 | 0.00173 | 1.32785 | 0.03523 | 0.14032 | 0.00181 | 867 | 34 | 858 | 15 | 846 | 10 |
| 12ER28-1-05 | 0.06755 | 0.00165 | 1.33349 | 0.03255 | 0.14138 | 0.00134 | 855 | 35 | 860 | 14 | 852 | 8 |
| 12ER28-1-06 | 0.06988 | 0.00155 | 1.06648 | 0.02354 | 0.10944 | 0.00111 | 925 | 29 | 737 | 12 | 670 | 6 |
| 12ER28-1-07 | 0.07536 | 0.00183 | 1.00937 | 0.02479 | 0.09605 | 0.00138 | 1078 | 27 | 709 | 13 | 591 | 8 |
| 12ER28-1-08 | 0.07138 | 0.00162 | 1.11657 | 0.02476 | 0.11162 | 0.00096 | 968 | 31 | 761 | 12 | 682 | 6 |
| 12ER28-1-09 | 0.06691 | 0.00166 | 1.32090 | 0.03984 | 0.14056 | 0.00275 | 835 | 32 | 855 | 17 | 848 | 16 |
| 12ER28-1-10 | 0.07049 | 0.00234 | 1.13966 | 0.03720 | 0.11482 | 0.00124 | 943 | 49 | 772 | 18 | 701 | 7 |
| 12ER28-1-11 | 0.06740 | 0.00181 | 1.11436 | 0.03014 | 0.11817 | 0.00120 | 850 | 39 | 760 | 14 | 720 | 7 |
| 12ER28-1-12 | 0.06621 | 0.00249 | 1.29572 | 0.05043 | 0.14079 | 0.00218 | 813 | 55 | 844 | 22 | 849 | 12 |
| 12ER28-1-13 | 0.06766 | 0.00191 | 1.32346 | 0.04015 | 0.14078 | 0.00211 | 858 | 38 | 856 | 18 | 849 | 12 |
| 12ER28-1-14 | 0.06893 | 0.00158 | 1.09264 | 0.02600 | 0.11375 | 0.00106 | 897 | 34 | 750 | 13 | 694 | 6 |
| 12ER28-1-15 | 0.07026 | 0.00170 | 1.56808 | 0.03815 | 0.16020 | 0.00148 | 936 | 35 | 958 | 15 | 958 | 8 |
| ER7-1-01 | 0.06749 | 0.00076 | 1.31407 | 0.02364 | 0.14096 | 0.00233 | 853 | 17 | 852 | 10 | 850 | 13 |
| ER7-1-02 | 0.06708 | 0.00082 | 1.30531 | 0.01584 | 0.14102 | 0.00123 | 840 | 12 | 848 | 7 | 850 | 7 |
| ER7-1-03 | 0.06777 | 0.00062 | 1.47912 | 0.01568 | 0.15806 | 0.00130 | 861 | 10 | 922 | 6 | 946 | 7 |
| ER7-1-05 | 0.06963 | 0.00042 | 1.52800 | 0.01912 | 0.15891 | 0.00174 | 918 | 11 | 942 | 8 | 951 | 10 |
| ER7-1-06 | 0.07074 | 0.00030 | 1.55364 | 0.01090 | 0.15906 | 0.00094 | 950 | 6 | 952 | 4 | 952 | 5 |
| ER7-1-08 | 0.06545 | 0.00069 | 1.27678 | 0.01679 | 0.14133 | 0.00134 | 789 | 13 | 835 | 7 | 852 | 8 |
| ER7-1-09 | 0.06691 | 0.00083 | 1.30010 | 0.01914 | 0.14084 | 0.00150 | 835 | 15 | 846 | 8 | 849 | 8 |
| ER7-1-10 | 0.06920 | 0.00177 | 1.34790 | 0.03338 | 0.14118 | 0.00087 | 905 | 41 | 867 | 14 | 851 | 5 |
| ER7-1-11 | 0.06896 | 0.00067 | 1.51339 | 0.01566 | 0.15920 | 0.00173 | 897 | 10 | 936 | 6 | 952 | 10 |
| ER7-1-12 | 0.07470 | 0.00139 | 1.82774 | 0.05019 | 0.17684 | 0.00297 | 1060 | 30 | 1055 | 18 | 1050 | 16 |
| ER7-1-13 | 0.07191 | 0.00040 | 1.57995 | 0.01419 | 0.15915 | 0.00114 | 983 | 8 | 962 | 6 | 952 | 6 |
| ER7-1-14 | 0.07011 | 0.00081 | 1.36298 | 0.01864 | 0.14116 | 0.00255 | 932 | 17 | 873 | 8 | 851 | 14 |
| ER7-1-15 | 0.06854 | 0.00046 | 1.50029 | 0.02345 | 0.15862 | 0.00223 | 885 | 14 | 931 | 10 | 949 | 12 |
| ER7-1-16 | 0.07058 | 0.00058 | 1.54922 | 0.02190 | 0.15897 | 0.00147 | 945 | 15 | 950 | 9 | 951 | 8 |
| ER7-1-18 | 0.07009 | 0.00115 | 1.53385 | 0.03021 | 0.15941 | 0.00279 | 931 | 18 | 944 | 12 | 954 | 16 |
| ER7-1-19 | 0.06985 | 0.00093 | 1.53494 | 0.02787 | 0.15933 | 0.00223 | 924 | 17 | 944 | 11 | 953 | 12 |
| ER7-1-20 | 0.07182 | 0.00114 | 1.75505 | 0.03303 | 0.17737 | 0.00332 | 981 | 17 | 1029 | 12 | 1053 | 18 |
| ER7-1-21 | 0.07115 | 0.00077 | 1.56279 | 0.02925 | 0.15902 | 0.00238 | 962 | 18 | 956 | 12 | 951 | 13 |
| ER7-1-22 | 0.06798 | 0.00071 | 1.48387 | 0.01865 | 0.15819 | 0.00136 | 868 | 13 | 924 | 8 | 947 | 8 |
| ER7-1-24 | 0.06765 | 0.00094 | 1.48467 | 0.02343 | 0.15898 | 0.00198 | 858 | 15 | 924 | 10 | 951 | 11 |
| ER7-1-25 | 0.07244 | 0.00153 | 1.58076 | 0.03039 | 0.15826 | 0.00136 | 998 | 44 | 963 | 12 | 947 | 8 |
| 14ER3-6-01 | 0.11277 | 0.00208 | 5.10324 | 0.09558 | 0.32506 | 0.00283 | 1845 | 21 | 1837 | 16 | 1814 | 14 |
| 14ER3-6-02 | 0.11349 | 0.00191 | 5.13539 | 0.08801 | 0.32515 | 0.00248 | 1856 | 20 | 1842 | 15 | 1815 | 12 |
| 14ER3-6-03 | 0.11267 | 0.00177 | 5.09175 | 0.08301 | 0.32495 | 0.00244 | 1843 | 19 | 1835 | 14 | 1814 | 12 |
| 14ER3-6-04 | 0.10973 | 0.00176 | 4.77550 | 0.08164 | 0.31306 | 0.00246 | 1795 | 20 | 1781 | 14 | 1756 | 12 |
| 14ER3-6-05 | 0.11323 | 0.00190 | 5.11074 | 0.09117 | 0.32502 | 0.00270 | 1852 | 20 | 1838 | 15 | 1814 | 13 |
| 14ER3-6-06 | 0.09629 | 0.00244 | 3.28198 | 0.07901 | 0.24721 | 0.00196 | 1553 | 49 | 1477 | 19 | 1424 | 10 |
| 14ER3-6-07 | 0.11328 | 0.00205 | 4.90588 | 0.09199 | 0.31257 | 0.00253 | 1853 | 22 | 1803 | 16 | 1753 | 12 |
| 14ER3-6-08 | 0.11181 | 0.00221 | 5.02758 | 0.10175 | 0.32479 | 0.00256 | 1829 | 25 | 1824 | 17 | 1813 | 12 |
| 14ER3-6-09 | 0.10934 | 0.00211 | 4.72160 | 0.09418 | 0.31177 | 0.00233 | 1788 | 25 | 1771 | 17 | 1749 | 11 |
| 14ER3-6-10 | 0.09897 | 0.00190 | 3.50780 | 0.06393 | 0.25705 | 0.00158 | 1605 | 37 | 1529 | 14 | 1475 | 8 |
| 14ER3-6-11 | 0.10904 | 0.00234 | 4.69636 | 0.09507 | 0.31236 | 0.00223 | 1784 | 40 | 1767 | 17 | 1752 | 11 |
| 14ER3-6-12 | 0.09885 | 0.00188 | 3.40522 | 0.06121 | 0.24986 | 0.00153 | 1602 | 36 | 1506 | 14 | 1438 | 8 |
| 14ER3-6-13 | 0.10404 | 0.00160 | 3.93794 | 0.06273 | 0.27311 | 0.00196 | 1697 | 19 | 1622 | 13 | 1557 | 10 |
| 14ER3-6-14 | 0.10630 | 0.00189 | 4.61624 | 0.08474 | 0.31330 | 0.00257 | 1737 | 22 | 1752 | 15 | 1757 | 13 |
| 14ER3-6-15 | 0.10712 | 0.00193 | 4.67693 | 0.08714 | 0.31482 | 0.00265 | 1751 | 22 | 1763 | 16 | 1764 | 13 |
| 14ER3-1-01 | 0.11440 | 0.00216 | 5.31566 | 0.09702 | 0.33308 | 0.00251 | 1871 | 22 | 1871 | 16 | 1853 | 12 |
| 14ER3-1-02 | 0.11489 | 0.00201 | 5.47471 | 0.09656 | 0.34183 | 0.00284 | 1878 | 20 | 1897 | 15 | 1895 | 14 |
| 14ER3-1-03 | 0.11363 | 0.00185 | 5.44546 | 0.08982 | 0.34398 | 0.00265 | 1858 | 19 | 1892 | 14 | 1906 | 13 |
| 14ER3-1-04 | 0.11237 | 0.00171 | 5.36301 | 0.08155 | 0.34294 | 0.00250 | 1838 | 17 | 1879 | 13 | 1901 | 12 |
| 14ER3-1-05 | 0.11411 | 0.00188 | 5.28982 | 0.08938 | 0.33366 | 0.00297 | 1866 | 18 | 1867 | 14 | 1856 | 14 |
| 14ER3-1-06 | 0.11218 | 0.00195 | 5.19467 | 0.09139 | 0.33309 | 0.00246 | 1835 | 21 | 1852 | 15 | 1853 | 12 |
| 14ER3-1-07 | 0.11030 | 0.00212 | 4.51963 | 0.08546 | 0.29512 | 0.00241 | 1804 | 22 | 1735 | 16 | 1667 | 12 |
| 14ER3-1-08 | 0.11061 | 0.00201 | 5.07074 | 0.09317 | 0.33026 | 0.00262 | 1809 | 22 | 1831 | 16 | 1840 | 13 |
| 14ER3-1-09 | 0.11129 | 0.00194 | 5.29395 | 0.09336 | 0.34264 | 0.00253 | 1821 | 21 | 1868 | 15 | 1899 | 12 |
| 14ER3-1-10 | 0.11259 | 0.00189 | 5.14418 | 0.09508 | 0.32908 | 0.00325 | 1842 | 19 | 1843 | 16 | 1834 | 16 |
| 14ER3-1-11 | 0.11533 | 0.00179 | 5.33829 | 0.08625 | 0.33326 | 0.00266 | 1885 | 18 | 1875 | 14 | 1854 | 13 |
| 14ER3-1-12 | 0.11199 | 0.00169 | 5.16449 | 0.09005 | 0.33150 | 0.00333 | 1832 | 18 | 1847 | 15 | 1846 | 16 |
| 14ER3-1-13 | 0.11230 | 0.00182 | 5.20085 | 0.08686 | 0.33327 | 0.00262 | 1837 | 19 | 1853 | 14 | 1854 | 13 |
| 14ER3-1-14 | 0.11347 | 0.00200 | 5.20526 | 0.09085 | 0.32996 | 0.00232 | 1856 | 21 | 1853 | 15 | 1838 | 11 |
| 14ER3-1-15 | 0.11316 | 0.00211 | 5.18662 | 0.09826 | 0.32958 | 0.00267 | 1851 | 23 | 1850 | 16 | 1836 | 13 |
| 14ER3-1-16 | 0.11319 | 0.00224 | 5.19348 | 0.10336 | 0.33048 | 0.00299 | 1851 | 23 | 1852 | 17 | 1841 | 14 |
| 14ER3-1-17 | 0.11697 | 0.00210 | 5.38697 | 0.10014 | 0.33147 | 0.00305 | 1910 | 20 | 1883 | 16 | 1846 | 15 |
| 14ER3-1-18 | 0.11728 | 0.00222 | 5.55918 | 0.10550 | 0.34264 | 0.00381 | 1915 | 19 | 1910 | 16 | 1899 | 18 |
| 14ER3-1-19 | 0.11830 | 0.00220 | 5.41945 | 0.10948 | 0.32947 | 0.00346 | 1931 | 21 | 1888 | 17 | 1836 | 17 |
| 14ER3-1-20 | 0.11342 | 0.00234 | 5.39336 | 0.11255 | 0.34189 | 0.00272 | 1855 | 26 | 1884 | 18 | 1896 | 13 |

**Supplementary** **Table S3. Zircon Hf isotopic data for the Proterozoic–Phanerozoic granitoids in the Erguna Massif**

| Sample | **t(Ma)** | ^176^Yb/^177^Hf | ^176^Lu/^177^Hf | ^176^Hf/^177^Hf | 2σ_m_ | ε_Hf_(0) | **ε_Hf_(t)** | 2σ | T_DM1_(Hf)(Ma) | **T_DM2_(Hf)(Ma)** | *f*_Lu/Hf_ |
| --- | --- | --- | --- | --- | --- | --- | --- | --- | --- | --- | --- |
| 11ER16-6-01 | **125** | 0.109713 | 0.004422 | 0.282819 | 0.000027 | 1.7 | **4.0** | 1.0 | 675 | **924** | -0.87 |
| 11ER16-6-02 | **125** | 0.067719 | 0.002620 | 0.282823 | 0.000078 | 1.8 | **4.3** | 2.8 | 636 | **906** | -0.92 |
| 11ER16-6-03 | **125** | 0.123087 | 0.004555 | 0.282815 | 0.000075 | 1.5 | **3.9** | 2.7 | 685 | **935** | -0.86 |
| 11ER16-6-04 | **125** | 0.100632 | 0.004349 | 0.282823 | 0.000062 | 1.8 | **4.2** | 2.2 | 667 | **914** | -0.87 |
| MZ14-2-01 | **134** | 0.034735 | 0.001109 | 0.282886 | 0.000032 | 4.0 | **6.9** | 1.1 | 520 | **750** | -0.97 |
| MZ14-2-02 | **134** | 0.024202 | 0.000928 | 0.282873 | 0.000023 | 3.6 | **6.4** | 0.8 | 536 | **777** | -0.97 |
| MZ14-2-03 | **134** | 0.017364 | 0.000594 | 0.282862 | 0.000033 | 3.2 | **6.1** | 1.2 | 547 | **801** | -0.98 |
| MZ14-2-04 | **134** | 0.034755 | 0.001202 | 0.282870 | 0.000033 | 3.5 | **6.3** | 1.2 | 544 | **786** | -0.96 |
| 12ER8-1-01 | **140** | 0.028320 | 0.000904 | 0.282896 | 0.000030 | 4.4 | **7.4** | 1.1 | 503 | **722** | -0.97 |
| 12ER8-1-02 | **140** | 0.081554 | 0.002313 | 0.282952 | 0.000042 | 6.4 | **9.2** | 1.5 | 440 | **603** | -0.93 |
| 12ER8-1-04 | **140** | 0.041426 | 0.001198 | 0.282883 | 0.000033 | 3.9 | **6.9** | 1.2 | 526 | **753** | -0.96 |
| 12ER8-1-05 | **140** | 0.039798 | 0.001195 | 0.282874 | 0.000038 | 3.6 | **6.6** | 1.3 | 539 | **774** | -0.96 |
| 12ER8-1-06 | **140** | 0.039420 | 0.001224 | 0.282875 | 0.000031 | 3.6 | **6.6** | 1.1 | 538 | **772** | -0.96 |
| 12ER8-1-07 | **140** | 0.031006 | 0.001049 | 0.282847 | 0.000032 | 2.7 | **5.6** | 1.1 | 575 | **834** | -0.97 |
| 12ER8-1-08 | **140** | 0.065595 | 0.002138 | 0.282927 | 0.000038 | 5.5 | **8.4** | 1.3 | 475 | **659** | -0.94 |
| 12ER8-1-09 | **140** | 0.009859 | 0.000432 | 0.282853 | 0.000035 | 2.9 | **5.9** | 1.2 | 557 | **817** | -0.99 |
| 12ER8-1-10 | **140** | 0.034760 | 0.001223 | 0.282885 | 0.000041 | 4.0 | **7.0** | 1.5 | 523 | **749** | -0.96 |
| 12ER8-1-14 | **140** | 0.043205 | 0.001397 | 0.282907 | 0.000037 | 4.8 | **7.7** | 1.3 | 494 | **700** | -0.96 |
| 17ER9-1-01 | **141** | 0.016298 | 0.000627 | 0.282959 | 0.000026 | 6.6 | **9.7** | 0.9 | 411 | **577** | -0.98 |
| 17ER9-1-02 | **141** | 0.018794 | 0.000749 | 0.282924 | 0.000020 | 5.4 | **8.4** | 0.7 | 462 | **657** | -0.98 |
| 17ER9-1-03 | **141** | 0.032019 | 0.001262 | 0.282881 | 0.000020 | 3.9 | **6.8** | 0.7 | 529 | **757** | -0.96 |
| 17ER9-1-04 | **141** | 0.038185 | 0.001556 | 0.282912 | 0.000028 | 5.0 | **7.9** | 1.0 | 488 | **688** | -0.95 |
| 17ER9-1-05 | **141** | 0.022027 | 0.000872 | 0.282910 | 0.000027 | 4.9 | **7.9** | 1.0 | 483 | **690** | -0.97 |
| 17ER9-1-06 | **141** | 0.040284 | 0.001468 | 0.282912 | 0.000024 | 5.0 | **7.9** | 0.9 | 488 | **689** | -0.96 |
| 17ER9-1-07 | **141** | 0.038463 | 0.001451 | 0.282883 | 0.000026 | 3.9 | **6.9** | 0.9 | 529 | **754** | -0.96 |
| 17ER9-1-08 | **141** | 0.035378 | 0.001390 | 0.282951 | 0.000025 | 6.3 | **9.3** | 0.9 | 431 | **600** | -0.96 |
| 17ER9-1-09 | **141** | 0.008680 | 0.000353 | 0.282937 | 0.000022 | 5.8 | **8.9** | 0.8 | 439 | **626** | -0.99 |
| 17ER9-1-10 | **141** | 0.018925 | 0.000750 | 0.282941 | 0.000026 | 6.0 | **9.0** | 0.9 | 437 | **618** | -0.98 |
| DB01-01 | **143** | 0.023635 | 0.000653 | 0.282880 | 0.000034 | 3.8 | **6.9** | 1.2 | 523 | **755** | -0.98 |
| DB01-02 | **143** | 0.034440 | 0.000909 | 0.282873 | 0.000032 | 3.6 | **6.6** | 1.1 | 536 | **772** | -0.97 |
| DB01-03 | **143** | 0.026209 | 0.000664 | 0.282878 | 0.000029 | 3.7 | **6.8** | 1.0 | 526 | **760** | -0.98 |
| DB01-04 | **143** | 0.021536 | 0.000579 | 0.282852 | 0.000036 | 2.8 | **5.9** | 1.3 | 561 | **819** | -0.98 |
| DB01-05 | **143** | 0.028922 | 0.000790 | 0.282881 | 0.000036 | 3.8 | **6.9** | 1.3 | 524 | **755** | -0.98 |
| DB01-06 | **143** | 0.075578 | 0.001801 | 0.282883 | 0.000052 | 3.9 | **6.9** | 1.8 | 534 | **754** | -0.95 |
| DB01-07 | **143** | 0.048874 | 0.001221 | 0.282901 | 0.000046 | 4.6 | **7.6** | 1.6 | 500 | **711** | -0.96 |
| DB01-08 | **143** | 0.035039 | 0.000951 | 0.282895 | 0.000035 | 4.3 | **7.4** | 1.2 | 506 | **723** | -0.97 |
| DB01-09 | **143** | 0.018232 | 0.000499 | 0.282882 | 0.000038 | 3.9 | **7.0** | 1.4 | 517 | **749** | -0.98 |
| DB01-10 | **143** | 0.022567 | 0.000611 | 0.282899 | 0.000036 | 4.5 | **7.6** | 1.3 | 495 | **711** | -0.98 |
| 13ER48-1-01 | **150** | 0.043333 | 0.001453 | 0.282741 | 0.000026 | -1.1 | **2.1** | 0.9 | 733 | **1070** | -0.96 |
| 13ER48-1 02 | **150** | 0.021742 | 0.000841 | 0.282749 | 0.000023 | -0.8 | **2.4** | 0.8 | 710 | **1048** | -0.97 |
| 13ER48-1-03 | **150** | 0.025096 | 0.000892 | 0.282701 | 0.000028 | -2.5 | **0.7** | 1.0 | 778 | **1157** | -0.97 |
| 13ER48-1-04 | **150** | 0.037863 | 0.001310 | 0.282710 | 0.000022 | -2.2 | **1.0** | 0.8 | 774 | **1139** | -0.96 |
| 13ER48-1-05 | **150** | 0.049236 | 0.001722 | 0.282786 | 0.000026 | 0.5 | **3.6** | 0.9 | 673 | **970** | -0.95 |
| 13ER48-1-06 | **150** | 0.027479 | 0.001035 | 0.282747 | 0.000027 | -0.9 | **2.3** | 1.0 | 716 | **1054** | -0.97 |
| 13ER48-1-07 | **150** | 0.037829 | 0.001349 | 0.282783 | 0.000031 | 0.4 | **3.5** | 1.1 | 671 | **975** | -0.96 |
| 13ER48-1-08 | **150** | 0.019507 | 0.000722 | 0.282733 | 0.000025 | -1.4 | **1.8** | 0.9 | 730 | **1084** | -0.98 |
| 12ER2-1-01 | **152** | 0.039709 | 0.001152 | 0.282877 | 0.000031 | 3.7 | **6.9** | 1.1 | 534 | **760** | -0.97 |
| 12ER2-1-02 | **152** | 0.042715 | 0.001296 | 0.282809 | 0.000027 | 1.3 | **4.5** | 1.0 | 633 | **914** | -0.96 |
| 12ER2-1-03 | **152** | 0.063027 | 0.001786 | 0.282841 | 0.000034 | 2.4 | **5.6** | 1.2 | 595 | **845** | -0.95 |
| 12ER2-1-04 | **152** | 0.048687 | 0.001537 | 0.282811 | 0.000031 | 1.4 | **4.6** | 1.1 | 634 | **911** | -0.95 |
| 12ER2-1-05 | **152** | 0.050415 | 0.001441 | 0.282859 | 0.000038 | 3.1 | **6.3** | 1.3 | 564 | **802** | -0.96 |
| 12ER2-1-06 | **152** | 0.021902 | 0.000771 | 0.282788 | 0.000030 | 0.6 | **3.8** | 1.1 | 654 | **959** | -0.98 |
| 12ER2-1-07 | **152** | 0.040914 | 0.001282 | 0.282816 | 0.000029 | 1.6 | **4.8** | 1.0 | 623 | **899** | -0.96 |
| 12ER2-1-08 | **152** | 0.038173 | 0.001129 | 0.282877 | 0.000030 | 3.7 | **6.9** | 1.1 | 533 | **759** | -0.97 |
| 12ER2-1-09 | **152** | 0.023398 | 0.000763 | 0.282789 | 0.000029 | 0.6 | **3.9** | 1.0 | 652 | **956** | -0.98 |
| 12ER2-1-10 | **152** | 0.054791 | 0.001641 | 0.282821 | 0.000034 | 1.7 | **4.9** | 1.2 | 621 | **890** | -0.95 |
| 12ER2-1-11 | **152** | 0.048870 | 0.001518 | 0.282810 | 0.000030 | 1.3 | **4.5** | 1.1 | 635 | **914** | -0.95 |
| Db-03-1 | **153** | 0.055841 | 0.001872 | 0.282868 | 0.000043 | 3.4 | **6.6** | 1.5 | 557 | **784** | -0.94 |
| Db-03-2 | **153** | 0.054611 | 0.001751 | 0.282864 | 0.000040 | 3.3 | **6.4** | 1.4 | 561 | **792** | -0.95 |
| Db-03-3 | **153** | 0.040446 | 0.001123 | 0.282873 | 0.000039 | 3.6 | **6.8** | 1.4 | 539 | **768** | -0.97 |
| Db-03-4 | **153** | 0.050308 | 0.001514 | 0.282971 | 0.000071 | 7.0 | **10.2** | 2.5 | 404 | **548** | -0.95 |
| Db-03-5 | **153** | 0.068593 | 0.002164 | 0.282866 | 0.000035 | 3.3 | **6.5** | 1.2 | 565 | **791** | -0.93 |
| Db-03-6 | **153** | 0.044912 | 0.001531 | 0.282863 | 0.000047 | 3.2 | **6.4** | 1.7 | 560 | **794** | -0.95 |
| Db-03-7 | **153** | 0.058291 | 0.001554 | 0.282983 | 0.000081 | 7.5 | **10.7** | 2.9 | 387 | **522** | -0.95 |
| 12ER1-2-01 | **155** | 0.017886 | 0.000726 | 0.282838 | 0.000019 | 2.3 | **5.7** | 0.7 | 582 | **842** | -0.98 |
| 12ER1-2-02 | **155** | 0.014853 | 0.000639 | 0.282826 | 0.000024 | 1.9 | **5.3** | 0.8 | 598 | **869** | -0.98 |
| 12ER1-2-03 | **155** | 0.017302 | 0.000687 | 0.282763 | 0.000027 | -0.3 | **3.0** | 1.0 | 688 | **1014** | -0.98 |
| 12ER1-2-04 | **155** | 0.016003 | 0.000660 | 0.282829 | 0.000031 | 2.0 | **5.4** | 1.1 | 594 | **863** | -0.98 |
| 12ER1-2-05 | **155** | 0.021189 | 0.000935 | 0.282811 | 0.000021 | 1.4 | **4.7** | 0.8 | 624 | **906** | -0.97 |
| 12ER1-2-06 | **155** | 0.033337 | 0.001529 | 0.282856 | 0.000021 | 3.0 | **6.2** | 0.7 | 570 | **809** | -0.95 |
| 12ER1-2-07 | **155** | 0.025752 | 0.001031 | 0.282646 | 0.000019 | -4.5 | **-1.2** | 0.7 | 859 | **1279** | -0.97 |
| 12ER1-2-08 | **155** | 0.052093 | 0.002006 | 0.282774 | 0.000020 | 0.1 | **3.3** | 0.7 | 695 | **995** | -0.94 |
| MZ23-1-01 | **171** | 0.055929 | 0.001799 | 0.282774 | 0.000036 | 0.1 | **3.6** | 1.3 | 692 | **985** | -0.95 |
| MZ23-1-02 | **171** | 0.040499 | 0.001294 | 0.282780 | 0.000035 | 0.3 | **3.9** | 1.2 | 674 | **969** | -0.96 |
| MZ23-1-03 | **171** | 0.036628 | 0.001145 | 0.282755 | 0.000032 | -0.6 | **3.0** | 1.1 | 707 | **1024** | -0.97 |
| MZ23-1-04 | **171** | 0.037017 | 0.001178 | 0.282836 | 0.000030 | 2.3 | **5.9** | 1.1 | 592 | **841** | -0.96 |
| MZ23-1-05 | **200** | 0.056353 | 0.001695 | 0.282783 | 0.000033 | 0.4 | **4.5** | 1.2 | 678 | **949** | -0.95 |
| MZ23-1-06 | **171** | 0.046871 | 0.001459 | 0.282767 | 0.000034 | -0.2 | **3.4** | 1.2 | 696 | **1000** | -0.96 |
| MZ23-1-07 | **171** | 0.035730 | 0.001109 | 0.282747 | 0.000031 | -0.9 | **2.7** | 1.1 | 718 | **1042** | -0.97 |
| MZ23-1-08 | **171** | 0.072356 | 0.002194 | 0.282792 | 0.000046 | 0.7 | **4.2** | 1.6 | 673 | **948** | -0.93 |
| MZ23-1-09 | **171** | 0.055082 | 0.001631 | 0.282787 | 0.000032 | 0.5 | **4.1** | 1.1 | 671 | **956** | -0.95 |
| 12ER31-1-01 | **173** | 0.025452 | 0.000724 | 0.282597 | 0.000019 | -6.2 | **-2.5** | 0.7 | 920 | **1376** | -0.98 |
| 12ER31-1-02 | **173** | 0.038340 | 0.000973 | 0.282645 | 0.000026 | -4.5 | **-0.8** | 0.9 | 858 | **1269** | -0.97 |
| 12ER31-1-03 | **173** | 0.017313 | 0.000650 | 0.282541 | 0.000023 | -8.2 | **-4.4** | 0.8 | 996 | **1501** | -0.98 |
| 12ER31-1-04 | **173** | 0.041465 | 0.001562 | 0.282647 | 0.000025 | -4.4 | **-0.8** | 0.9 | 870 | **1270** | -0.95 |
| 12ER31-1-05 | **173** | 0.037626 | 0.001080 | 0.282576 | 0.000020 | -6.9 | **-3.2** | 0.7 | 958 | **1425** | -0.97 |
| 12ER31-1-06 | **173** | 0.031837 | 0.000999 | 0.282642 | 0.000025 | -4.6 | **-0.9** | 0.9 | 863 | **1277** | -0.97 |
| 12ER31-1-07 | **173** | 0.030235 | 0.001219 | 0.282597 | 0.000033 | -6.2 | **-2.5** | 1.2 | 932 | **1380** | -0.96 |
| 14ER14-1-01 | **177** | 0.014233 | 0.000628 | 0.282565 | 0.000032 | -7.3 | **-3.5** | 1.1 | 963 | **1445** | -0.98 |
| 14ER14-1-02 | **177** | 0.007835 | 0.000406 | 0.282579 | 0.000023 | -6.8 | **-3.0** | 0.8 | 938 | **1412** | -0.99 |
| 14ER14-1-03 | **177** | 0.008342 | 0.000436 | 0.282562 | 0.000026 | -7.4 | **-3.6** | 0.9 | 962 | **1450** | -0.99 |
| 14ER14-1-04 | **177** | 0.012151 | 0.000584 | 0.282580 | 0.000024 | -6.8 | **-3.0** | 0.9 | 941 | **1412** | -0.98 |
| 14ER14-1-05 | **177** | 0.014750 | 0.000621 | 0.282592 | 0.000023 | -6.4 | **-2.6** | 0.8 | 925 | **1385** | -0.98 |
| 14ER14-1-06 | **177** | 0.012064 | 0.000613 | 0.282565 | 0.000026 | -7.3 | **-3.5** | 0.9 | 962 | **1445** | -0.98 |
| 14ER14-1-07 | **177** | 0.019572 | 0.000884 | 0.282529 | 0.000035 | -8.6 | **-4.8** | 1.2 | 1019 | **1527** | -0.97 |
| 14ER14-1-08 | **177** | 0.007851 | 0.000397 | 0.282553 | 0.000026 | -7.7 | **-3.9** | 0.9 | 973 | **1470** | -0.99 |
| MZ18-2-01 | **180** | 0.095439 | 0.002416 | 0.282985 | 0.000017 | 7.5 | **11.2** | 0.6 | 393 | **508** | -0.93 |
| MZ18-2-02 | **180** | 0.041855 | 0.001101 | 0.282847 | 0.000012 | 2.7 | **6.5** | 0.4 | 575 | **810** | -0.97 |
| MZ18-2-03 | **180** | 0.041757 | 0.001539 | 0.282892 | 0.000037 | 4.2 | **8.0** | 1.3 | 518 | **713** | -0.95 |
| MZ18-2-04 | **180** | 0.064838 | 0.001738 | 0.282871 | 0.000013 | 3.5 | **7.3** | 0.5 | 551 | **761** | -0.95 |
| MZ18-2-05 | **180** | 0.056922 | 0.001539 | 0.282862 | 0.000011 | 3.2 | **6.9** | 0.4 | 561 | **780** | -0.95 |
| MZ18-2-06 | **180** | 0.113224 | 0.002944 | 0.282940 | 0.000027 | 6.0 | **9.6** | 1.0 | 466 | **613** | -0.91 |
| MZ18-2-07 | **180** | 0.034814 | 0.000954 | 0.282843 | 0.000012 | 2.5 | **6.4** | 0.4 | 579 | **818** | -0.97 |
| MZ18-2-08 | **180** | 0.066738 | 0.001817 | 0.282902 | 0.000012 | 4.6 | **8.3** | 0.4 | 507 | **691** | -0.95 |
| ZKS1-1-01 | **212** | 0.207317 | 0.004934 | 0.283044 | 0.000032 | 9.6 | **13.6** | 1.1 | 328 | **378** | -0.85 |
| ZKS1-1-02 | **212** | 0.127309 | 0.002773 | 0.282887 | 0.000038 | 4.1 | **8.3** | 1.3 | 543 | **716** | -0.92 |
| ZKS1-1-03 | **185** | 0.085396 | 0.001962 | 0.282812 | 0.000017 | 1.4 | **5.3** | 0.6 | 639 | **893** | -0.94 |
| ZKS1-1-04 | **185** | 0.115257 | 0.002794 | 0.282874 | 0.000024 | 3.6 | **7.3** | 0.9 | 562 | **759** | -0.92 |
| ZKS1-1-05 | **212** | 0.136013 | 0.003106 | 0.282886 | 0.000015 | 4.0 | **8.3** | 0.5 | 549 | **721** | -0.91 |
| ZKS1-1-06 | **185** | 0.111315 | 0.002710 | 0.282901 | 0.000023 | 4.6 | **8.3** | 0.8 | 521 | **698** | -0.92 |
| ZKS1-1-07 | **185** | 0.120621 | 0.002806 | 0.282882 | 0.000013 | 3.9 | **7.6** | 0.5 | 551 | **742** | -0.92 |
| ZKS1-1-08 | **185** | 0.107327 | 0.002422 | 0.282894 | 0.000042 | 4.3 | **8.1** | 1.5 | 528 | **712** | -0.93 |
| ZKS1-1-09 | **212** | 0.206318 | 0.004371 | 0.283013 | 0.000029 | 8.5 | **12.6** | 1.0 | 372 | **445** | -0.87 |
| ZKS1-1-10 | **185** | 0.096342 | 0.002215 | 0.282857 | 0.000018 | 3.0 | **6.8** | 0.6 | 579 | **794** | -0.93 |
| ZKS1-1-11 | **185** | 0.125296 | 0.002732 | 0.282899 | 0.000018 | 4.5 | **8.2** | 0.7 | 525 | **702** | -0.92 |
| ZKS1-1-12 | **185** | 0.074505 | 0.001737 | 0.282801 | 0.000019 | 1.0 | **4.9** | 0.7 | 652 | **917** | -0.95 |
| ZKS1-1-13 | **185** | 0.119376 | 0.002846 | 0.282888 | 0.000022 | 4.1 | **7.8** | 0.8 | 542 | **728** | -0.91 |
| ZKS1-1-14 | **185** | 0.075996 | 0.001761 | 0.282776 | 0.000014 | 0.1 | **4.0** | 0.5 | 688 | **973** | -0.95 |
| ZKS1-1-15 | **240** | 0.144555 | 0.003213 | 0.282909 | 0.000035 | 4.8 | **9.6** | 1.3 | 516 | **656** | -0.90 |
| ZKS1-1-16 | **185** | 0.111158 | 0.002532 | 0.282859 | 0.000017 | 3.1 | **6.8** | 0.6 | 581 | **792** | -0.92 |
| 13ER14-1-01 | **185** | 0.043180 | 0.001672 | 0.282845 | 0.000031 | 2.6 | **6.4** | 1.1 | 587 | **816** | -0.95 |
| 13ER14-1-02 | **185** | 0.049954 | 0.002158 | 0.282831 | 0.000039 | 2.1 | **5.9** | 1.4 | 616 | **853** | -0.94 |
| 13ER14-1-03 | **185** | 0.066452 | 0.002858 | 0.282737 | 0.000021 | -1.2 | **2.5** | 0.7 | 768 | **1071** | -0.91 |
| 13ER14-1-04 | **185** | 0.043033 | 0.001595 | 0.282686 | 0.000026 | -3.0 | **0.8** | 0.9 | 814 | **1175** | -0.95 |
| 13ER14-1-05 | **185** | 0.031376 | 0.001190 | 0.282746 | 0.000022 | -0.9 | **3.0** | 0.8 | 721 | **1038** | -0.96 |
| 13ER14-1-06 | **185** | 0.033433 | 0.001346 | 0.282633 | 0.000029 | -4.9 | **-1.0** | 1.0 | 885 | **1293** | -0.96 |
| 13ER14-1-07 | **185** | 0.061867 | 0.002268 | 0.282866 | 0.000032 | 3.3 | **7.1** | 1.1 | 566 | **774** | -0.93 |
| 13ER14-1-08 | **185** | 0.063726 | 0.002686 | 0.282747 | 0.000023 | -0.9 | **2.8** | 0.8 | 750 | **1047** | -0.92 |
| 13ER14-1-09 | **185** | 0.065817 | 0.002575 | 0.282718 | 0.000043 | -1.9 | **1.8** | 1.5 | 790 | **1111** | -0.92 |
| 13ER14-1-10 | **185** | 0.059988 | 0.002482 | 0.282772 | 0.000029 | 0.0 | **3.8** | 1.0 | 708 | **989** | -0.93 |
| ER6-1-01 | **186** | 0.071791 | 0.002019 | 0.282811 | 0.000062 | 1.4 | **5.2** | 2.2 | 642 | **895** | -0.94 |
| ER6-1-02 | **186** | 0.139558 | 0.003813 | 0.282840 | 0.000052 | 2.4 | **6.0** | 1.8 | 631 | **844** | -0.89 |
| ER6-1-03 | **186** | 0.068620 | 0.001934 | 0.282754 | 0.000034 | -0.6 | **3.2** | 1.2 | 724 | **1024** | -0.94 |
| ER6-1-04 | **186** | 0.112901 | 0.003113 | 0.282840 | 0.000043 | 2.4 | **6.1** | 1.5 | 619 | **838** | -0.91 |
| ER6-1-05 | **186** | 0.165896 | 0.004501 | 0.282902 | 0.000063 | 4.6 | **8.1** | 2.2 | 548 | **710** | -0.86 |
| ER6-1-06 | **206** | 0.243614 | 0.006524 | 0.283075 | 0.000076 | 10.7 | **14.4** | 2.7 | 293 | **325** | -0.80 |
| ER6-1-07 | **186** | 0.104637 | 0.002854 | 0.282900 | 0.000023 | 4.5 | **8.3** | 0.8 | 525 | **701** | -0.91 |
| ER6-1-08 | **186** | 0.071228 | 0.001938 | 0.282742 | 0.000016 | -1.1 | **2.8** | 0.6 | 742 | **1052** | -0.94 |
| ER6-1-09 | **186** | 0.173408 | 0.004564 | 0.282999 | 0.000057 | 8.0 | **11.6** | 2.0 | 396 | **489** | -0.86 |
| ER6-1-10 | **186** | 0.117904 | 0.003156 | 0.282869 | 0.000024 | 3.4 | **7.1** | 0.9 | 576 | **774** | -0.90 |
| ER6-1-11 | **206** | 0.168243 | 0.004338 | 0.282886 | 0.000040 | 4.0 | **8.0** | 1.4 | 569 | **735** | -0.87 |
| 13ER21-1-01 | **195** | 0.062641 | 0.002280 | 0.282729 | 0.000029 | -1.5 | **2.5** | 1.0 | 767 | **1077** | -0.93 |
| 13ER21-1-02 | **195** | 0.038746 | 0.001619 | 0.282694 | 0.000020 | -2.7 | **1.3** | 0.7 | 803 | **1151** | -0.95 |
| 13ER21-1-03 | **195** | 0.040314 | 0.001565 | 0.282709 | 0.000022 | -2.2 | **1.9** | 0.8 | 781 | **1117** | -0.95 |
| 13ER21-1-04 | **195** | 0.039321 | 0.001556 | 0.282723 | 0.000021 | -1.7 | **2.4** | 0.8 | 760 | **1085** | -0.95 |
| 13ER21-1-05 | **195** | 0.050769 | 0.001991 | 0.282698 | 0.000023 | -2.6 | **1.4** | 0.8 | 806 | **1145** | -0.94 |
| 13ER21-1-06 | **195** | 0.039429 | 0.001633 | 0.282733 | 0.000021 | -1.4 | **2.7** | 0.8 | 748 | **1063** | -0.95 |
| 13ER21-1-07 | **195** | 0.043908 | 0.001610 | 0.282768 | 0.000024 | -0.1 | **3.9** | 0.8 | 697 | **984** | -0.95 |
| 13ER6-1-01 | **196** | 0.041712 | 0.001643 | 0.282747 | 0.000027 | -0.9 | **3.2** | 1.0 | 728 | **1032** | -0.95 |
| 13ER6-1-02 | **196** | 0.074720 | 0.002870 | 0.282833 | 0.000035 | 2.2 | **6.1** | 1.2 | 625 | **847** | -0.91 |
| 13ER6-1-03 | **196** | 0.083904 | 0.003182 | 0.282767 | 0.000027 | -0.2 | **3.7** | 0.9 | 730 | **999** | -0.90 |
| 13ER6-1-04 | **196** | 0.070744 | 0.002757 | 0.282776 | 0.000031 | 0.2 | **4.1** | 1.1 | 707 | **974** | -0.92 |
| 13ER6-1-05 | **196** | 0.043448 | 0.001695 | 0.282736 | 0.000030 | -1.3 | **2.8** | 1.1 | 745 | **1057** | -0.95 |
| 13ER6-1-06 | **196** | 0.049415 | 0.001914 | 0.282748 | 0.000026 | -0.9 | **3.2** | 0.9 | 732 | **1032** | -0.94 |
| 13ER6-1-07 | **196** | 0.065257 | 0.002569 | 0.282759 | 0.000028 | -0.5 | **3.5** | 1.0 | 730 | **1013** | -0.92 |
| 13TH1-1-01 | **196** | 0.043431 | 0.001489 | 0.282687 | 0.000029 | -3.0 | **1.1** | 1.0 | 811 | **1165** | -0.96 |
| 13TH1-1-02 | **196** | 0.045347 | 0.001616 | 0.282704 | 0.000024 | -2.4 | **1.7** | 0.8 | 789 | **1129** | -0.95 |
| 13TH1-1-03 | **196** | 0.081433 | 0.002711 | 0.282736 | 0.000035 | -1.3 | **2.7** | 1.2 | 765 | **1064** | -0.92 |
| 13TH1-1-04 | **196** | 0.062633 | 0.002072 | 0.282669 | 0.000028 | -3.6 | **0.4** | 1.0 | 850 | **1211** | -0.94 |
| 13TH1-1-05 | **196** | 0.030344 | 0.001043 | 0.282684 | 0.000023 | -3.1 | **1.0** | 0.8 | 806 | **1170** | -0.97 |
| 13TH1-1-06 | **196** | 0.050809 | 0.001823 | 0.282648 | 0.000029 | -4.4 | **-0.3** | 1.0 | 874 | **1257** | -0.95 |
| 13TH1-1-07 | **196** | 0.035483 | 0.001198 | 0.282688 | 0.000022 | -3.0 | **1.2** | 0.8 | 803 | **1161** | -0.96 |
| 13TH1-1-08 | **196** | 0.086305 | 0.002969 | 0.282691 | 0.000029 | -2.9 | **1.1** | 1.0 | 838 | **1168** | -0.91 |
| 13TH1-1-09 | **196** | 0.038613 | 0.001286 | 0.282652 | 0.000033 | -4.2 | **-0.1** | 1.2 | 856 | **1243** | -0.96 |
| 13TH1-1-10 | **196** | 0.041219 | 0.001423 | 0.282676 | 0.000029 | -3.4 | **0.7** | 1.0 | 825 | **1190** | -0.96 |
| 12ER27-1-01 | **197** | 0.038489 | 0.001392 | 0.282795 | 0.000033 | 0.8 | **5.0** | 1.2 | 654 | **920** | -0.96 |
| 12ER27-1-02 | **197** | 0.026707 | 0.000939 | 0.282778 | 0.000029 | 0.2 | **4.4** | 1.0 | 670 | **955** | -0.97 |
| 12ER27-1-03 | **197** | 0.035635 | 0.001254 | 0.282789 | 0.000030 | 0.6 | **4.8** | 1.1 | 660 | **932** | -0.96 |
| 12ER27-1-04 | **197** | 0.037379 | 0.001303 | 0.282760 | 0.000030 | -0.4 | **3.7** | 1.1 | 703 | **999** | -0.96 |
| 12ER27-1-05 | **197** | 0.034414 | 0.001198 | 0.282769 | 0.000034 | -0.1 | **4.1** | 1.2 | 688 | **977** | -0.96 |
| 12ER27-1-06 | **197** | 0.034360 | 0.001307 | 0.282754 | 0.000033 | -0.6 | **3.5** | 1.2 | 712 | **1013** | -0.96 |
| 12ER27-1-07 | **197** | 0.025820 | 0.000880 | 0.282752 | 0.000035 | -0.7 | **3.5** | 1.3 | 706 | **1013** | -0.97 |
| 12ER27-1-08 | **197** | 0.037725 | 0.001323 | 0.282761 | 0.000036 | -0.4 | **3.8** | 1.3 | 701 | **996** | -0.96 |
| 12ER27-1-09 | **197** | 0.041731 | 0.001404 | 0.282767 | 0.000038 | -0.2 | **4.0** | 1.3 | 695 | **984** | -0.96 |
| 12ER27-1-10 | **197** | 0.033276 | 0.001157 | 0.282773 | 0.000033 | 0.0 | **4.2** | 1.2 | 682 | **968** | -0.97 |
| 12ER27-1-11 | **197** | 0.040048 | 0.001360 | 0.282775 | 0.000035 | 0.1 | **4.2** | 1.2 | 683 | **966** | -0.96 |
| 12ER27-1-12 | **197** | 0.053468 | 0.001750 | 0.282760 | 0.000031 | -0.4 | **3.7** | 1.1 | 711 | **1002** | -0.95 |
| 12ER27-1-13 | **197** | 0.042532 | 0.001433 | 0.282765 | 0.000029 | -0.3 | **3.9** | 1.0 | 698 | **989** | -0.96 |
| 11ER10-1-01 | **202** | 0.045578 | 0.001642 | 0.282672 | 0.000012 | -3.5 | **0.7** | 0.4 | 835 | **1197** | -0.95 |
| 11ER10-1-02 | **235** | 0.048351 | 0.001677 | 0.282689 | 0.000014 | -2.9 | **2.0** | 0.5 | 812 | **1141** | -0.95 |
| 11ER10-1-03 | **202** | 0.030587 | 0.001077 | 0.282673 | 0.000016 | -3.5 | **0.8** | 0.6 | 821 | **1190** | -0.97 |
| 11ER10-1-04 | **202** | 0.023157 | 0.000843 | 0.282679 | 0.000011 | -3.3 | **1.0** | 0.4 | 809 | **1175** | -0.97 |
| 11ER10-1-05 | **202** | 0.068149 | 0.002432 | 0.282720 | 0.000019 | -1.8 | **2.3** | 0.7 | 784 | **1096** | -0.93 |
| 11ER10-1-06 | **202** | 0.048630 | 0.001683 | 0.282687 | 0.000013 | -3.0 | **1.2** | 0.5 | 814 | **1163** | -0.95 |
| 11ER10-1-07 | **215** | 0.028842 | 0.001046 | 0.282657 | 0.000015 | -4.1 | **0.5** | 0.5 | 843 | **1218** | -0.97 |
| 11ER10-1-08 | **215** | 0.033794 | 0.001183 | 0.282668 | 0.000011 | -3.7 | **0.9** | 0.4 | 831 | **1194** | -0.96 |
| 11ER10-1-09 | **202** | 0.028703 | 0.001009 | 0.282665 | 0.000012 | -3.8 | **0.5** | 0.4 | 832 | **1208** | -0.97 |
| 11ER10-1-10 | **202** | 0.049014 | 0.001570 | 0.282700 | 0.000010 | -2.5 | **1.7** | 0.4 | 794 | **1133** | -0.95 |
| 11ER13-1-01 | **244** | 0.062411 | 0.001840 | 0.282768 | 0.000032 | -0.2 | **4.9** | 1.1 | 702 | **960** | -0.94 |
| 11ER13-1-02 | **244** | 0.064897 | 0.002030 | 0.282729 | 0.000034 | -1.5 | **3.5** | 1.2 | 762 | **1050** | -0.94 |
| 11ER13-1-03 | **203** | 0.052373 | 0.001741 | 0.282713 | 0.000041 | -2.1 | **2.1** | 1.4 | 779 | **1105** | -0.95 |
| 11ER13-1-04 | **244** | 0.035747 | 0.001065 | 0.282767 | 0.000036 | -0.2 | **5.0** | 1.3 | 689 | **954** | -0.97 |
| 11ER13-1-05 | **244** | 0.093222 | 0.002671 | 0.282739 | 0.000036 | -1.2 | **3.8** | 1.3 | 761 | **1033** | -0.92 |
| 11ER13-1-06 | **244** | 0.083871 | 0.002499 | 0.282753 | 0.000038 | -0.7 | **4.3** | 1.4 | 736 | **999** | -0.92 |
| 11ER13-1-07 | **244** | 0.068992 | 0.002265 | 0.282722 | 0.000043 | -1.8 | **3.2** | 1.5 | 776 | **1066** | -0.93 |
| 11ER13-1-08 | **244** | 0.048135 | 0.001456 | 0.282722 | 0.000035 | -1.8 | **3.4** | 1.2 | 760 | **1058** | -0.96 |
| 11ER13-1-09 | **244** | 0.082563 | 0.002566 | 0.282738 | 0.000037 | -1.2 | **3.7** | 1.3 | 760 | **1034** | -0.92 |
| 11ER13-1-10 | **244** | 0.048668 | 0.001452 | 0.282715 | 0.000033 | -2.0 | **3.1** | 1.2 | 770 | **1075** | -0.96 |
| 11ER13-1-11 | **244** | 0.048163 | 0.001452 | 0.282734 | 0.000031 | -1.3 | **3.8** | 1.1 | 743 | **1032** | -0.96 |
| 11ER13-1-12 | **203** | 0.048970 | 0.001480 | 0.282741 | 0.000029 | -1.1 | **3.2** | 1.0 | 733 | **1039** | -0.96 |
| 11ER13-1-13 | **244** | 0.125397 | 0.003983 | 0.282822 | 0.000052 | 1.8 | **6.5** | 1.8 | 661 | **858** | -0.88 |
| 11ER13-1-14 | **229** | 0.093319 | 0.002791 | 0.282754 | 0.000038 | -0.6 | **4.0** | 1.4 | 741 | **1008** | -0.92 |
| 12ER17-1-01 | **205** | 0.032202 | 0.000880 | 0.282597 | 0.000019 | -6.2 | **-1.8** | 0.7 | 924 | **1357** | -0.97 |
| 12ER17-1-02 | **205** | 0.036544 | 0.000987 | 0.282633 | 0.000018 | -4.9 | **-0.6** | 0.6 | 877 | **1279** | -0.97 |
| 12ER17-1-03 | **205** | 0.038566 | 0.001054 | 0.282618 | 0.000023 | -5.5 | **-1.1** | 0.8 | 899 | **1313** | -0.97 |
| 12ER17-1-04 | **205** | 0.043350 | 0.001142 | 0.282620 | 0.000018 | -5.4 | **-1.0** | 0.6 | 899 | **1310** | -0.97 |
| 12ER17-1-05 | **205** | 0.056847 | 0.001351 | 0.282656 | 0.000020 | -4.1 | **0.2** | 0.7 | 852 | **1230** | -0.96 |
| 12ER17-1-06 | **205** | 0.047879 | 0.001259 | 0.282617 | 0.000017 | -5.5 | **-1.1** | 0.6 | 905 | **1316** | -0.96 |
| 12ER17-1-07 | **205** | 0.035741 | 0.000938 | 0.282606 | 0.000016 | -5.9 | **-1.5** | 0.5 | 913 | **1339** | -0.97 |
| 12ER17-1-08 | **205** | 0.044349 | 0.001153 | 0.282620 | 0.000016 | -5.4 | **-1.0** | 0.6 | 898 | **1309** | -0.97 |
| 12ER17-1-09 | **205** | 0.047086 | 0.001457 | 0.282645 | 0.000034 | -4.5 | **-0.2** | 1.2 | 870 | **1254** | -0.96 |
| 12ER17-1-10 | **205** | 0.056322 | 0.001434 | 0.282623 | 0.000016 | -5.3 | **-0.9** | 0.6 | 900 | **1303** | -0.96 |
| 12ER17-1-11 | **205** | 0.040506 | 0.001192 | 0.282600 | 0.000017 | -6.1 | **-1.7** | 0.6 | 928 | **1354** | -0.96 |
| 12ER17-1-12 | **205** | 0.136744 | 0.004039 | 0.282663 | 0.000026 | -3.9 | **0.1** | 0.9 | 908 | **1237** | -0.88 |
| 12ER19-1-01 | **205** | 0.064721 | 0.001921 | 0.282719 | 0.000034 | -1.9 | **2.4** | 1.2 | 773 | **1091** | -0.94 |
| 12ER19-1-02 | **205** | 0.042042 | 0.001312 | 0.282731 | 0.000028 | -1.4 | **2.9** | 1.0 | 744 | **1059** | -0.96 |
| 12ER19-1-03 | **205** | 0.047858 | 0.001449 | 0.282703 | 0.000041 | -2.4 | **1.9** | 1.4 | 787 | **1124** | -0.96 |
| 12ER19-1-04 | **205** | 0.037335 | 0.001151 | 0.282682 | 0.000032 | -3.2 | **1.1** | 1.1 | 811 | **1170** | -0.97 |
| 12ER19-1-05 | **205** | 0.037095 | 0.001143 | 0.282702 | 0.000027 | -2.5 | **1.9** | 1.0 | 782 | **1124** | -0.97 |
| 12ER19-1-06 | **205** | 0.042645 | 0.001279 | 0.282722 | 0.000026 | -1.8 | **2.6** | 0.9 | 757 | **1080** | -0.96 |
| 12ER19-1-07 | **205** | 0.054234 | 0.001639 | 0.282729 | 0.000035 | -1.5 | **2.8** | 1.2 | 754 | **1068** | -0.95 |
| 12ER19-1-08 | **205** | 0.058413 | 0.001705 | 0.282761 | 0.000031 | -0.4 | **3.9** | 1.1 | 709 | **995** | -0.95 |
| 12ER19-1-09 | **205** | 0.047032 | 0.001417 | 0.282719 | 0.000029 | -1.9 | **2.4** | 1.0 | 764 | **1088** | -0.96 |
| 12ER19-1-10 | **205** | 0.038686 | 0.001126 | 0.282739 | 0.000027 | -1.2 | **3.2** | 0.9 | 729 | **1040** | -0.97 |
| 14ER18-1-01 | **205** | 0.018996 | 0.000842 | 0.282790 | 0.000024 | 0.6 | **5.0** | 0.8 | 652 | **923** | -0.97 |
| 14ER18-1-02 | **205** | 0.023044 | 0.000989 | 0.282776 | 0.000024 | 0.1 | **4.5** | 0.8 | 675 | **956** | -0.97 |
| 14ER18-1-03 | **205** | 0.039424 | 0.001627 | 0.282779 | 0.000026 | 0.2 | **4.5** | 0.9 | 682 | **955** | -0.95 |
| 14ER18-1-04 | **205** | 0.026312 | 0.001136 | 0.282787 | 0.000022 | 0.5 | **4.9** | 0.8 | 661 | **931** | -0.97 |
| 14ER18-1-05 | **205** | 0.026247 | 0.001123 | 0.282783 | 0.000029 | 0.4 | **4.7** | 1.0 | 667 | **942** | -0.97 |
| 14ER18-1-06 | **205** | 0.041288 | 0.001676 | 0.282775 | 0.000025 | 0.1 | **4.4** | 0.9 | 689 | **964** | -0.95 |
| 14ER18-1-07 | **205** | 0.027808 | 0.001197 | 0.282786 | 0.000027 | 0.5 | **4.8** | 0.9 | 664 | **934** | -0.96 |
| 14ER18-1-08 | **205** | 0.037642 | 0.001623 | 0.282778 | 0.000025 | 0.2 | **4.5** | 0.9 | 683 | **956** | -0.95 |
| 14ER18-1-09 | **205** | 0.056513 | 0.002042 | 0.282783 | 0.000035 | 0.4 | **4.6** | 1.2 | 683 | **948** | -0.94 |
| 14ER18-1-10 | **205** | 0.047773 | 0.001912 | 0.282780 | 0.000050 | 0.3 | **4.5** | 1.8 | 685 | **953** | -0.94 |
| 18ER12-1-01 | **206** | 0.045475 | 0.001519 | 0.282679 | 0.000027 | -3.3 | **1.0** | 1.0 | 822 | **1178** | -0.95 |
| 18ER12-1-02 | **206** | 0.068163 | 0.002269 | 0.282580 | 0.000029 | -6.8 | **-2.6** | 1.0 | 985 | **1409** | -0.93 |
| 18ER12-1-03 | **206** | 0.071412 | 0.002060 | 0.282639 | 0.000029 | -4.7 | **-0.5** | 1.0 | 893 | **1273** | -0.94 |
| 18ER12-1-04 | **206** | 0.078000 | 0.002267 | 0.282610 | 0.000028 | -5.7 | **-1.5** | 1.0 | 941 | **1340** | -0.93 |
| 18ER12-1-05 | **206** | 0.047071 | 0.001397 | 0.282600 | 0.000026 | -6.1 | **-1.7** | 0.9 | 933 | **1355** | -0.96 |
| 18ER12-1-06 | **206** | 0.087180 | 0.002481 | 0.282590 | 0.000039 | -6.4 | **-2.2** | 1.4 | 975 | **1386** | -0.93 |
| 18ER12-1-07 | **206** | 0.056860 | 0.001752 | 0.282623 | 0.000031 | -5.3 | **-1.0** | 1.1 | 909 | **1307** | -0.95 |
| 18ER12-1-08 | **206** | 0.053514 | 0.001604 | 0.282606 | 0.000025 | -5.9 | **-1.6** | 0.9 | 929 | **1344** | -0.95 |
| 18ER12-1-09 | **206** | 0.036394 | 0.001139 | 0.282628 | 0.000024 | -5.1 | **-0.7** | 0.8 | 887 | **1290** | -0.97 |
| 18ER12-1-10 | **206** | 0.040533 | 0.001482 | 0.282602 | 0.000025 | -6.0 | **-1.7** | 0.9 | 931 | **1351** | -0.96 |
| 12ER34-1-01 | **206** | 0.037394 | 0.000940 | 0.282751 | 0.000021 | -0.7 | **3.7** | 0.7 | 709 | **1011** | -0.97 |
| 12ER34-1-02 | **206** | 0.035743 | 0.000927 | 0.282759 | 0.000028 | -0.5 | **3.9** | 1.0 | 698 | **994** | -0.97 |
| 12ER34-1-03 | **206** | 0.033573 | 0.000863 | 0.282736 | 0.000020 | -1.3 | **3.1** | 0.7 | 728 | **1044** | -0.97 |
| 12ER34-1-04 | **206** | 0.032457 | 0.000844 | 0.282737 | 0.000022 | -1.2 | **3.2** | 0.8 | 727 | **1041** | -0.97 |
| 12ER34-1-05 | **206** | 0.033532 | 0.000863 | 0.282728 | 0.000020 | -1.6 | **2.9** | 0.7 | 740 | **1062** | -0.97 |
| 12ER34-1-06 | **206** | 0.033075 | 0.000869 | 0.282734 | 0.000021 | -1.3 | **3.1** | 0.7 | 731 | **1048** | -0.97 |
| 12ER34-1-07 | **206** | 0.032970 | 0.000849 | 0.282745 | 0.000019 | -1.0 | **3.5** | 0.7 | 716 | **1024** | -0.97 |
| 12ER34-1-08 | **206** | 0.024396 | 0.000640 | 0.282757 | 0.000021 | -0.5 | **3.9** | 0.8 | 695 | **996** | -0.98 |
| 12ER34-1-09 | **206** | 0.037380 | 0.000954 | 0.282740 | 0.000020 | -1.1 | **3.3** | 0.7 | 724 | **1035** | -0.97 |
| 12ER34-1-10 | **206** | 0.033775 | 0.000899 | 0.282738 | 0.000020 | -1.2 | **3.2** | 0.7 | 727 | **1041** | -0.97 |
| 12ER34-1-11 | **206** | 0.039241 | 0.001026 | 0.282753 | 0.000018 | -0.7 | **3.7** | 0.6 | 707 | **1006** | -0.97 |
| 12ER34-1-12 | **206** | 0.036087 | 0.000927 | 0.282738 | 0.000019 | -1.2 | **3.2** | 0.7 | 728 | **1041** | -0.97 |
| 12ER34-1-13 | **206** | 0.033459 | 0.000892 | 0.282742 | 0.000019 | -1.1 | **3.3** | 0.7 | 721 | **1031** | -0.97 |
| 12ER34-1-14 | **206** | 0.032427 | 0.000883 | 0.282735 | 0.000018 | -1.3 | **3.1** | 0.7 | 730 | **1046** | -0.97 |
| 12ER34-1-15 | **206** | 0.035747 | 0.000936 | 0.282753 | 0.000017 | -0.7 | **3.7** | 0.6 | 706 | **1007** | -0.97 |
| 11ER26-1-01 | **221** | 0.057115 | 0.001832 | 0.282714 | 0.000018 | -2.1 | **2.5** | 0.6 | 780 | **1094** | -0.94 |
| 11ER26-1-02 | **206** | 0.031234 | 0.000977 | 0.282686 | 0.000011 | -3.0 | **1.3** | 0.4 | 801 | **1158** | -0.97 |
| 11ER26-1-03 | **221** | 0.042072 | 0.001374 | 0.282682 | 0.000010 | -3.2 | **1.5** | 0.4 | 816 | **1162** | -0.96 |
| 11ER26-1-04 | **221** | 0.031293 | 0.001061 | 0.282667 | 0.000013 | -3.7 | **1.0** | 0.4 | 830 | **1193** | -0.97 |
| 11ER26-1-05 | **221** | 0.031297 | 0.000991 | 0.282675 | 0.000011 | -3.4 | **1.3** | 0.4 | 817 | **1175** | -0.97 |
| 11ER26-1-06 | **221** | 0.040905 | 0.001347 | 0.282684 | 0.000013 | -3.1 | **1.5** | 0.4 | 812 | **1157** | -0.96 |
| 11ER26-1-07 | **221** | 0.047693 | 0.001645 | 0.282663 | 0.000020 | -3.9 | **0.7** | 0.7 | 849 | **1208** | -0.95 |
| 11ER26-1-08 | **221** | 0.081557 | 0.002482 | 0.282750 | 0.000021 | -0.8 | **3.7** | 0.7 | 740 | **1018** | -0.93 |
| 11ER26-1-09 | **221** | 0.042665 | 0.001399 | 0.282701 | 0.000017 | -2.5 | **2.1** | 0.6 | 790 | **1120** | -0.96 |
| 11ER26-1-10 | **206** | 0.047169 | 0.001671 | 0.282667 | 0.000034 | -3.7 | **0.6** | 1.2 | 843 | **1206** | -0.95 |
| 11ER26-1-11 | **206** | 0.036378 | 0.001114 | 0.282696 | 0.000012 | -2.7 | **1.7** | 0.4 | 790 | **1137** | -0.97 |
| 11ER26-1-12 | **206** | 0.044340 | 0.001361 | 0.282670 | 0.000013 | -3.6 | **0.7** | 0.5 | 832 | **1197** | -0.96 |
| 11ER26-1-13 | **221** | 0.041738 | 0.001287 | 0.282663 | 0.000015 | -3.8 | **0.8** | 0.5 | 840 | **1203** | -0.96 |
| 12ER16-3-01 | **206** | 0.062049 | 0.001871 | 0.282661 | 0.000031 | -3.9 | **0.3** | 1.1 | 857 | **1222** | -0.94 |
| 12ER16-3-02 | **206** | 0.038259 | 0.001230 | 0.282666 | 0.000033 | -3.8 | **0.6** | 1.2 | 835 | **1206** | -0.96 |
| 12ER16-3-03 | **206** | 0.046916 | 0.001439 | 0.282673 | 0.000030 | -3.5 | **0.8** | 1.1 | 830 | **1191** | -0.96 |
| 12ER16-3-04 | **206** | 0.034225 | 0.001122 | 0.282661 | 0.000033 | -3.9 | **0.5** | 1.2 | 839 | **1215** | -0.97 |
| 12ER16-3-05 | **206** | 0.041595 | 0.001332 | 0.282667 | 0.000034 | -3.7 | **0.6** | 1.2 | 835 | **1203** | -0.96 |
| 12ER16-3-06 | **206** | 0.037580 | 0.001199 | 0.282652 | 0.000036 | -4.2 | **0.1** | 1.3 | 854 | **1236** | -0.96 |
| 12ER16-3-07 | **206** | 0.025838 | 0.000836 | 0.282652 | 0.000031 | -4.2 | **0.2** | 1.1 | 846 | **1233** | -0.97 |
| 12ER16-3-08 | **206** | 0.034466 | 0.001174 | 0.282653 | 0.000037 | -4.2 | **0.2** | 1.3 | 852 | **1234** | -0.96 |
| 12ER16-3-09 | **206** | 0.045864 | 0.001396 | 0.282648 | 0.000035 | -4.4 | **-0.1** | 1.2 | 865 | **1248** | -0.96 |
| 18ER19-1-01 | **211** | 0.030540 | 0.000954 | 0.282655 | 0.000036 | -4.1 | **0.4** | 1.3 | 844 | **1223** | -0.97 |
| 18ER19-1-02 | **211** | 0.030963 | 0.000900 | 0.282644 | 0.000025 | -4.5 | **0.0** | 0.9 | 858 | **1248** | -0.97 |
| 18ER19-1-03 | **211** | 0.035755 | 0.001093 | 0.282647 | 0.000020 | -4.4 | **0.0** | 0.7 | 859 | **1245** | -0.97 |
| 18ER19-1-04 | **211** | 0.028456 | 0.000926 | 0.282648 | 0.000027 | -4.4 | **0.1** | 1.0 | 853 | **1239** | -0.97 |
| 18ER19-1-05 | **211** | 0.031898 | 0.000976 | 0.282636 | 0.000033 | -4.8 | **-0.3** | 1.2 | 872 | **1268** | -0.97 |
| 18ER19-1-06 | **211** | 0.027766 | 0.000852 | 0.282652 | 0.000036 | -4.2 | **0.3** | 1.3 | 846 | **1230** | -0.97 |
| 18ER19-1-07 | **211** | 0.022499 | 0.000737 | 0.282647 | 0.000035 | -4.4 | **0.1** | 1.2 | 851 | **1241** | -0.98 |
| 18ER19-1-08 | **211** | 0.034986 | 0.001129 | 0.282647 | 0.000024 | -4.4 | **0.0** | 0.9 | 860 | **1245** | -0.97 |
| 18ER19-1-09 | **211** | 0.018168 | 0.000569 | 0.282656 | 0.000029 | -4.1 | **0.4** | 1.0 | 835 | **1220** | -0.98 |
| 18ER19-1-10 | **211** | 0.024723 | 0.000804 | 0.282653 | 0.000033 | -4.2 | **0.3** | 1.2 | 843 | **1227** | -0.98 |
| 11ER9-1-01 | **224** | 0.035308 | 0.000965 | 0.282597 | 0.000035 | -6.2 | **-1.4** | 1.2 | 926 | **1346** | -0.97 |
| 11ER9-1-02 | **224** | 0.039596 | 0.001103 | 0.282601 | 0.000040 | -6.1 | **-1.3** | 1.4 | 924 | **1340** | -0.97 |
| 11ER9-1-03 | **224** | 0.043882 | 0.001261 | 0.282634 | 0.000033 | -4.9 | **-0.2** | 1.2 | 882 | **1268** | -0.96 |
| 11ER9-1-04 | **224** | 0.084871 | 0.002198 | 0.282614 | 0.000039 | -5.6 | **-1.0** | 1.4 | 933 | **1322** | -0.93 |
| 11ER9-1-05 | **224** | 0.081639 | 0.002112 | 0.282599 | 0.000034 | -6.1 | **-1.5** | 1.2 | 952 | **1352** | -0.94 |
| 11ER9-1-06 | **224** | 0.124313 | 0.002854 | 0.282634 | 0.000056 | -4.9 | **-0.4** | 2.0 | 921 | **1283** | -0.91 |
| 11ER9-1-07 | **224** | 0.110571 | 0.002707 | 0.282636 | 0.000041 | -4.8 | **-0.3** | 1.5 | 914 | **1276** | -0.92 |
| 13ER46-1-01 | **228** | 0.019772 | 0.000767 | 0.282758 | 0.000024 | -0.5 | **4.4** | 0.8 | 695 | **979** | -0.98 |
| 13ER46-1-02 | **228** | 0.018588 | 0.000754 | 0.282784 | 0.000023 | 0.4 | **5.3** | 0.8 | 660 | **922** | -0.98 |
| 13ER46-1-03 | **228** | 0.019896 | 0.000795 | 0.282811 | 0.000023 | 1.4 | **6.3** | 0.8 | 622 | **861** | -0.98 |
| 13ER46-1-04 | **228** | 0.030769 | 0.001193 | 0.282801 | 0.000026 | 1.0 | **5.9** | 0.9 | 643 | **887** | -0.96 |
| 13ER46-1-05 | **228** | 0.020472 | 0.000822 | 0.282767 | 0.000027 | -0.2 | **4.7** | 0.9 | 685 | **961** | -0.98 |
| 13ER46-1-06 | **228** | 0.025038 | 0.000987 | 0.282757 | 0.000024 | -0.5 | **4.3** | 0.9 | 702 | **985** | -0.97 |
| 13ER46-1-07 | **228** | 0.025676 | 0.001026 | 0.282792 | 0.000028 | 0.7 | **5.6** | 1.0 | 653 | **906** | -0.97 |
| ER12-1-01 | **229** | 0.078853 | 0.002508 | 0.282704 | 0.000045 | -2.4 | **2.2** | 1.6 | 809 | **1118** | -0.92 |
| ER12-1-02 | **229** | 0.082543 | 0.001998 | 0.282726 | 0.000038 | -1.6 | **3.1** | 1.4 | 765 | **1063** | -0.94 |
| ER12-1-03 | **229** | 0.090563 | 0.002706 | 0.282734 | 0.000066 | -1.3 | **3.3** | 2.3 | 769 | **1052** | -0.92 |
| ER12-1-05 | **229** | 0.029391 | 0.000918 | 0.282673 | 0.000031 | -3.5 | **1.4** | 1.1 | 818 | **1172** | -0.97 |
| ER12-1-06 | **229** | 0.118881 | 0.002661 | 0.282751 | 0.000071 | -0.7 | **3.9** | 2.5 | 742 | **1013** | -0.92 |
| ER12-1-07 | **229** | 0.355243 | 0.007911 | 0.282803 | 0.000098 | 1.1 | **4.9** | 3.5 | 780 | **947** | -0.76 |
| 15ER12-1-01 | **238** | 0.034927 | 0.001540 | 0.282854 | 0.000022 | 2.9 | **7.9** | 0.8 | 572 | **764** | -0.95 |
| 15ER12-1-02 | **238** | 0.031030 | 0.001397 | 0.282875 | 0.000022 | 3.6 | **8.7** | 0.8 | 540 | **715** | -0.96 |
| 15ER12-1-03 | **238** | 0.034499 | 0.001526 | 0.282852 | 0.000022 | 2.8 | **7.8** | 0.8 | 574 | **768** | -0.95 |
| 15ER12-1-04 | **238** | 0.029975 | 0.001204 | 0.282890 | 0.000021 | 4.2 | **9.2** | 0.8 | 516 | **679** | -0.96 |
| 15ER12-1-05 | **238** | 0.027818 | 0.001214 | 0.282857 | 0.000023 | 3.0 | **8.0** | 0.8 | 564 | **756** | -0.96 |
| 15ER12-1-06 | **238** | 0.026133 | 0.001161 | 0.282838 | 0.000022 | 2.4 | **7.4** | 0.8 | 589 | **796** | -0.97 |
| 15ER12-1-07 | **238** | 0.026578 | 0.001155 | 0.282902 | 0.000025 | 4.6 | **9.7** | 0.9 | 498 | **652** | -0.97 |
| 15ER12-1-08 | **238** | 0.024914 | 0.001017 | 0.282820 | 0.000021 | 1.7 | **6.8** | 0.7 | 612 | **836** | -0.97 |
| 15ER12-1-09 | **238** | 0.043456 | 0.001953 | 0.282858 | 0.000024 | 3.0 | **8.0** | 0.9 | 573 | **760** | -0.94 |
| 15ER12-1-10 | **238** | 0.038071 | 0.001646 | 0.282853 | 0.000026 | 2.9 | **7.8** | 0.9 | 575 | **768** | -0.95 |
| 15ER4-1-01 | **239** | 0.053408 | 0.002103 | 0.282687 | 0.000036 | -3.0 | **1.9** | 1.3 | 825 | **1148** | -0.94 |
| 15ER4-1-02 | **239** | 0.041687 | 0.001613 | 0.282655 | 0.000037 | -4.2 | **0.8** | 1.3 | 860 | **1215** | -0.95 |
| 15ER4-1-03 | **239** | 0.045486 | 0.001769 | 0.282586 | 0.000041 | -6.6 | **-1.6** | 1.5 | 962 | **1370** | -0.95 |
| 15ER4-1-04 | **239** | 0.047902 | 0.001867 | 0.282651 | 0.000040 | -4.3 | **0.7** | 1.4 | 871 | **1226** | -0.94 |
| 15ER4-1-05 | **239** | 0.049861 | 0.001927 | 0.282601 | 0.000033 | -6.1 | **-1.1** | 1.2 | 945 | **1339** | -0.94 |
| 15ER4-1-06 | **239** | 0.071677 | 0.002757 | 0.282697 | 0.000039 | -2.6 | **2.2** | 1.4 | 824 | **1130** | -0.92 |
| 15ER4-1-07 | **239** | 0.102188 | 0.003834 | 0.282718 | 0.000055 | -1.9 | **2.7** | 1.9 | 818 | **1094** | -0.88 |
| 15ER4-1-08 | **239** | 0.043091 | 0.001707 | 0.282654 | 0.000037 | -4.2 | **0.8** | 1.3 | 863 | **1218** | -0.95 |
| 15ER4-1-09 | **239** | 0.070951 | 0.002608 | 0.282699 | 0.000050 | -2.6 | **2.3** | 1.8 | 818 | **1125** | -0.92 |
| 15ER4-1-10 | **239** | 0.080288 | 0.003089 | 0.282690 | 0.000040 | -2.9 | **1.9** | 1.4 | 842 | **1149** | -0.91 |
| ER15-1-01 | **241** | 0.019039 | 0.000666 | 0.282613 | 0.000031 | -5.6 | **-0.4** | 1.1 | 897 | **1299** | -0.98 |
| ER15-1-02 | **241** | 0.023335 | 0.000676 | 0.282611 | 0.000026 | -5.7 | **-0.5** | 0.9 | 900 | **1304** | -0.98 |
| ER15-1-03 | **241** | 0.037614 | 0.001078 | 0.282587 | 0.000031 | -6.5 | **-1.4** | 1.1 | 943 | **1361** | -0.97 |
| ER15-1-04 | **241** | 0.033319 | 0.000946 | 0.282608 | 0.000035 | -5.8 | **-0.6** | 1.2 | 910 | **1312** | -0.97 |
| ER15-1-05 | **241** | 0.022068 | 0.000642 | 0.282599 | 0.000035 | -6.1 | **-0.9** | 1.2 | 916 | **1330** | -0.98 |
| ER15-1-06 | **241** | 0.018933 | 0.000566 | 0.282601 | 0.000027 | -6.0 | **-0.8** | 1.0 | 911 | **1324** | -0.98 |
| ER15-1-07 | **241** | 0.038296 | 0.001083 | 0.282591 | 0.000033 | -6.4 | **-1.3** | 1.2 | 938 | **1352** | -0.97 |
| ER15-1-08 | **241** | 0.016592 | 0.000476 | 0.282588 | 0.000028 | -6.5 | **-1.3** | 1.0 | 927 | **1353** | -0.99 |
| ER15-1-09 | **241** | 0.028085 | 0.000788 | 0.282600 | 0.000039 | -6.1 | **-0.9** | 1.4 | 918 | **1329** | -0.98 |
| ER15-1-10 | **241** | 0.024727 | 0.000685 | 0.282570 | 0.000035 | -7.1 | **-2.0** | 1.2 | 957 | **1395** | -0.98 |
| ER15-1-11 | **241** | 0.033005 | 0.000877 | 0.282615 | 0.000033 | -5.6 | **-0.4** | 1.2 | 900 | **1297** | -0.97 |
| 11ER17-1-01 | **242** | 0.033156 | 0.000981 | 0.282759 | 0.000033 | -0.5 | **4.7** | 1.2 | 698 | **971** | -0.97 |
| 11ER17-1-02 | **242** | 0.072909 | 0.002068 | 0.282777 | 0.000036 | 0.2 | **5.2** | 1.3 | 693 | **942** | -0.94 |
| 11ER17-1-03 | **242** | 0.064322 | 0.001904 | 0.282817 | 0.000046 | 1.6 | **6.6** | 1.6 | 631 | **849** | -0.94 |
| 11ER17-1-04 | **242** | 0.034763 | 0.001025 | 0.282746 | 0.000025 | -0.9 | **4.2** | 0.9 | 717 | **1001** | -0.97 |
| 11ER17-1-05 | **242** | 0.069464 | 0.002084 | 0.282772 | 0.000034 | 0.0 | **5.0** | 1.2 | 700 | **953** | -0.94 |
| 13ER31-1-01 | **246** | 0.014140 | 0.000554 | 0.282664 | 0.000025 | -3.8 | **1.5** | 0.9 | 822 | **1178** | -0.98 |
| 13ER31-1-02 | **246** | 0.017065 | 0.000701 | 0.282603 | 0.000023 | -6.0 | **-0.7** | 0.8 | 912 | **1318** | -0.98 |
| 13ER31-1-03 | **246** | 0.029761 | 0.001191 | 0.282669 | 0.000026 | -3.7 | **1.6** | 0.9 | 830 | **1175** | -0.96 |
| 13ER31-1-04 | **246** | 0.021103 | 0.000857 | 0.282675 | 0.000021 | -3.4 | **1.8** | 0.7 | 814 | **1157** | -0.97 |
| 13ER31-1-05 | **246** | 0.015602 | 0.000611 | 0.282633 | 0.000022 | -4.9 | **0.4** | 0.8 | 868 | **1251** | -0.98 |
| 11ER18-1-01 | **247** | 0.035346 | 0.001209 | 0.282632 | 0.000012 | -4.9 | **0.3** | 0.4 | 882 | **1256** | -0.96 |
| 11ER18-1-02 | **247** | 0.021499 | 0.000764 | 0.282614 | 0.000011 | -5.6 | **-0.3** | 0.4 | 897 | **1293** | -0.98 |
| 11ER18-1-03 | **247** | 0.021476 | 0.000782 | 0.282609 | 0.000012 | -5.8 | **-0.5** | 0.4 | 905 | **1305** | -0.98 |
| 11ER18-1-04 | **247** | 0.026173 | 0.000939 | 0.282612 | 0.000010 | -5.7 | **-0.4** | 0.3 | 905 | **1300** | -0.97 |
| 11ER18-1-05 | **247** | 0.015882 | 0.000576 | 0.282627 | 0.000013 | -5.1 | **0.2** | 0.5 | 875 | **1262** | -0.98 |
| 11ER18-1-06 | **247** | 0.030672 | 0.001027 | 0.282707 | 0.000015 | -2.3 | **2.9** | 0.5 | 773 | **1087** | -0.97 |
| 11ER18-1-07 | **247** | 0.021806 | 0.000770 | 0.282628 | 0.000012 | -5.1 | **0.2** | 0.4 | 878 | **1261** | -0.98 |
| 11ER18-1-08 | **247** | 0.027835 | 0.001008 | 0.282609 | 0.000015 | -5.7 | **-0.5** | 0.5 | 910 | **1306** | -0.97 |
| 11ER18-1-09 | **247** | 0.047427 | 0.001631 | 0.282635 | 0.000019 | -4.8 | **0.3** | 0.7 | 888 | **1254** | -0.95 |
| 11ER18-1-10 | **247** | 0.020571 | 0.000744 | 0.282621 | 0.000010 | -5.3 | **0.0** | 0.4 | 888 | **1278** | -0.98 |
| 11ER18-1-11 | **247** | 0.029103 | 0.001073 | 0.282650 | 0.000011 | -4.3 | **0.9** | 0.4 | 855 | **1216** | -0.97 |
| 11ER18-1-12 | **247** | 0.066570 | 0.002048 | 0.282718 | 0.000013 | -1.9 | **3.2** | 0.5 | 778 | **1071** | -0.94 |
| 11ER18-1-13 | **247** | 0.020309 | 0.000763 | 0.282647 | 0.000009 | -4.4 | **0.9** | 0.3 | 851 | **1219** | -0.98 |
| 14ER16-1-01 | **256** | 0.017869 | 0.000744 | 0.282589 | 0.000024 | -6.5 | **-1.0** | 0.8 | 932 | **1344** | -0.98 |
| 14ER16-1-02 | **256** | 0.019891 | 0.000834 | 0.282590 | 0.000018 | -6.4 | **-1.0** | 0.6 | 933 | **1343** | -0.97 |
| 14ER16-1-03 | **256** | 0.020662 | 0.000859 | 0.282596 | 0.000019 | -6.2 | **-0.7** | 0.7 | 925 | **1329** | -0.97 |
| 14ER16-1-04 | **256** | 0.021944 | 0.000931 | 0.282588 | 0.000018 | -6.5 | **-1.0** | 0.6 | 938 | **1348** | -0.97 |
| 14ER16-1-05 | **256** | 0.031726 | 0.001317 | 0.282693 | 0.000029 | -2.8 | **2.6** | 1.0 | 799 | **1117** | -0.96 |
| 14ER16-1-06 | **256** | 0.026742 | 0.001113 | 0.282637 | 0.000023 | -4.8 | **0.7** | 0.8 | 873 | **1240** | -0.97 |
| 14ER16-1-07 | **256** | 0.022315 | 0.000925 | 0.282603 | 0.000018 | -6.0 | **-0.5** | 0.6 | 917 | **1314** | -0.97 |
| 14ER16-1-08 | **256** | 0.019418 | 0.000825 | 0.282589 | 0.000017 | -6.5 | **-1.0** | 0.6 | 934 | **1345** | -0.98 |
| 14ER16-1-09 | **256** | 0.029505 | 0.001201 | 0.282660 | 0.000027 | -4.0 | **1.5** | 1.0 | 843 | **1189** | -0.96 |
| 14ER16-1-10 | **256** | 0.021221 | 0.000876 | 0.282609 | 0.000022 | -5.8 | **-0.3** | 0.8 | 907 | **1300** | -0.97 |
| 15ER1-1-01 | **303** | 0.021710 | 0.000935 | 0.282905 | 0.000052 | 4.7 | **11.2** | 1.8 | 491 | **604** | -0.97 |
| 15ER1-1-02 | **303** | 0.021904 | 0.000945 | 0.282887 | 0.000045 | 4.1 | **10.6** | 1.6 | 516 | **644** | -0.97 |
| 15ER1-1-03 | **303** | 0.020652 | 0.000903 | 0.282894 | 0.000037 | 4.3 | **10.8** | 1.3 | 506 | **628** | -0.97 |
| 15ER1-1-04 | **303** | 0.014204 | 0.000635 | 0.282896 | 0.000034 | 4.4 | **10.9** | 1.2 | 500 | **621** | -0.98 |
| 15ER1-1-05 | **303** | 0.017452 | 0.000798 | 0.282884 | 0.000030 | 4.0 | **10.5** | 1.1 | 519 | **650** | -0.98 |
| 15ER1-1-06 | **303** | 0.012903 | 0.000620 | 0.282929 | 0.000047 | 5.5 | **12.1** | 1.7 | 453 | **546** | -0.98 |
| 15ER1-1-07 | **303** | 0.019871 | 0.000900 | 0.282939 | 0.000036 | 5.9 | **12.4** | 1.3 | 443 | **527** | -0.97 |
| 15ER1-1-08 | **303** | 0.018882 | 0.000832 | 0.282891 | 0.000041 | 4.2 | **10.7** | 1.4 | 510 | **635** | -0.97 |
| 15ER1-1-09 | **303** | 0.022424 | 0.001019 | 0.282885 | 0.000033 | 4.0 | **10.5** | 1.2 | 520 | **649** | -0.97 |
| 15ER1-1-10 | **303** | 0.022558 | 0.001049 | 0.282887 | 0.000032 | 4.1 | **10.5** | 1.1 | 518 | **646** | -0.97 |
| 12ER36-1-01 | **455** | 0.109689 | 0.003491 | 0.282666 | 0.000029 | -3.7 | **5.2** | 1.0 | 889 | **1102** | -0.89 |
| 12ER36-1-02 | **455** | 0.068031 | 0.002201 | 0.282639 | 0.000016 | -4.7 | **4.7** | 0.6 | 897 | **1138** | -0.93 |
| 12ER36-1-03 | **455** | 0.081149 | 0.002607 | 0.282684 | 0.000028 | -3.1 | **6.1** | 1.0 | 840 | **1044** | -0.92 |
| 12ER36-1-04 | **455** | 0.065145 | 0.001995 | 0.282595 | 0.000017 | -6.3 | **3.2** | 0.6 | 955 | **1233** | -0.94 |
| 12ER36-1-05 | **455** | 0.088007 | 0.002515 | 0.282631 | 0.000026 | -5.0 | **4.3** | 0.9 | 916 | **1162** | -0.92 |
| 12ER36-1-06 | **455** | 0.087130 | 0.002752 | 0.282644 | 0.000016 | -4.5 | **4.7** | 0.6 | 903 | **1137** | -0.92 |
| 12ER36-1-07 | **455** | 0.070143 | 0.002311 | 0.282603 | 0.000018 | -6.0 | **3.3** | 0.6 | 952 | **1221** | -0.93 |
| 12ER36-1-08 | **455** | 0.049812 | 0.001456 | 0.282603 | 0.000018 | -6.0 | **3.6** | 0.6 | 930 | **1205** | -0.96 |
| 12ER36-1-09 | **455** | 0.080497 | 0.002538 | 0.282669 | 0.000034 | -3.6 | **5.6** | 1.2 | 861 | **1077** | -0.92 |
| 12ER36-1-10 | **455** | 0.073152 | 0.002275 | 0.282563 | 0.000013 | -7.4 | **1.9** | 0.5 | 1009 | **1310** | -0.93 |
| 12ER36-1-11 | **455** | 0.086483 | 0.002689 | 0.282595 | 0.000024 | -6.3 | **2.9** | 0.8 | 974 | **1246** | -0.92 |
| 12ER36-1-13 | **455** | 0.068219 | 0.002059 | 0.282653 | 0.000020 | -4.2 | **5.2** | 0.7 | 873 | **1104** | -0.94 |
| 12ER36-1-15 | **455** | 0.049832 | 0.001485 | 0.282594 | 0.000012 | -6.3 | **3.3** | 0.4 | 943 | **1226** | -0.96 |
| 12ER36-1-16 | **455** | 0.088093 | 0.002737 | 0.282640 | 0.000022 | -4.7 | **4.5** | 0.8 | 908 | **1146** | -0.92 |
| 12ER36-1-17 | **455** | 0.150450 | 0.004623 | 0.282620 | 0.000023 | -5.4 | **3.2** | 0.8 | 990 | **1227** | -0.86 |
| 12ER36-1-19 | **455** | 0.067537 | 0.002121 | 0.282605 | 0.000015 | -5.9 | **3.5** | 0.5 | 944 | **1213** | -0.94 |
| 12ER36-1-22 | **455** | 0.097964 | 0.002960 | 0.282619 | 0.000020 | -5.4 | **3.7** | 0.7 | 945 | **1198** | -0.91 |
| 12ER36-1-23 | **455** | 0.082178 | 0.002451 | 0.282614 | 0.000021 | -5.6 | **3.7** | 0.7 | 939 | **1199** | -0.93 |
| 12ER36-1-24 | **455** | 0.069532 | 0.002177 | 0.282561 | 0.000018 | -7.5 | **1.9** | 0.6 | 1009 | **1313** | -0.93 |
| 18ER23-1-01 | **460** | 0.035257 | 0.001232 | 0.282531 | 0.000032 | -8.5 | **1.2** | 1.1 | 1026 | **1360** | -0.96 |
| 18ER23-1-02 | **460** | 0.032006 | 0.001088 | 0.282515 | 0.000031 | -9.1 | **0.7** | 1.1 | 1044 | **1392** | -0.97 |
| 18ER23-1-03 | **460** | 0.043418 | 0.001435 | 0.282533 | 0.000033 | -8.5 | **1.2** | 1.2 | 1029 | **1359** | -0.96 |
| 18ER23-1-04 | **460** | 0.035250 | 0.001184 | 0.282511 | 0.000029 | -9.2 | **0.5** | 1.0 | 1054 | **1404** | -0.96 |
| 18ER23-1-05 | **460** | 0.044144 | 0.001521 | 0.282517 | 0.000034 | -9.0 | **0.6** | 1.2 | 1054 | **1396** | -0.95 |
| 18ER23-1-06 | **460** | 0.038591 | 0.001287 | 0.282507 | 0.000027 | -9.4 | **0.3** | 1.0 | 1062 | **1415** | -0.96 |
| 18ER23-1-07 | **460** | 0.033202 | 0.001165 | 0.282542 | 0.000025 | -8.1 | **1.6** | 0.9 | 1009 | **1333** | -0.96 |
| 18ER23-1-08 | **460** | 0.037587 | 0.001344 | 0.282511 | 0.000024 | -9.2 | **0.5** | 0.8 | 1058 | **1407** | -0.96 |
| 18ER23-1-09 | **460** | 0.048940 | 0.001705 | 0.282526 | 0.000029 | -8.7 | **0.9** | 1.0 | 1047 | **1380** | -0.95 |
| 18ER23-1-10 | **460** | 0.029153 | 0.001019 | 0.282525 | 0.000026 | -8.7 | **1.1** | 0.9 | 1029 | **1369** | -0.97 |
| 14ER10-1-01 | **465** | 0.129318 | 0.003501 | 0.282545 | 0.000036 | -8.0 | **1.1** | 1.3 | 1071 | **1368** | -0.89 |
| 14ER10-1-02 | **465** | 0.110768 | 0.003361 | 0.282539 | 0.000036 | -8.2 | **1.0** | 1.3 | 1076 | **1380** | -0.90 |
| 14ER10-1-03 | **465** | 0.025447 | 0.000834 | 0.282513 | 0.000014 | -9.2 | **0.8** | 0.5 | 1041 | **1389** | -0.97 |
| 14ER6-1-01 | **476** | 0.019813 | 0.000852 | 0.282518 | 0.000019 | -9.0 | **1.2** | 0.7 | 1035 | **1372** | -0.97 |
| 14ER6-1-02 | **476** | 0.016421 | 0.000697 | 0.282549 | 0.000016 | -7.9 | **2.4** | 0.6 | 987 | **1300** | -0.98 |
| 14ER6-1-03 | **476** | 0.019321 | 0.000838 | 0.282542 | 0.000020 | -8.1 | **2.1** | 0.7 | 1000 | **1318** | -0.97 |
| 14ER6-1-04 | **476** | 0.020526 | 0.000873 | 0.282544 | 0.000017 | -8.1 | **2.1** | 0.6 | 999 | **1315** | -0.97 |
| 14ER6-1-05 | **476** | 0.021607 | 0.000916 | 0.282511 | 0.000017 | -9.2 | **0.9** | 0.6 | 1046 | **1390** | -0.97 |
| 14ER6-1-06 | **476** | 0.009488 | 0.000396 | 0.282523 | 0.000018 | -8.8 | **1.5** | 0.6 | 1015 | **1351** | -0.99 |
| 14ER6-1-07 | **476** | 0.020021 | 0.000848 | 0.282507 | 0.000022 | -9.4 | **0.8** | 0.8 | 1050 | **1397** | -0.97 |
| 14ER6-1-08 | **476** | 0.016757 | 0.000721 | 0.282532 | 0.000018 | -8.5 | **1.8** | 0.6 | 1011 | **1337** | -0.98 |
| 14ER6-1-09 | **476** | 0.018802 | 0.000832 | 0.282515 | 0.000016 | -9.1 | **1.1** | 0.6 | 1037 | **1377** | -0.97 |
| 14ER6-1-10 | **476** | 0.022453 | 0.000918 | 0.282557 | 0.000022 | -7.6 | **2.6** | 0.8 | 981 | **1285** | -0.97 |
| 14ER15-1-01 | **479** | 0.037173 | 0.001492 | 0.282505 | 0.000019 | -9.4 | **0.6** | 0.7 | 1070 | **1411** | -0.96 |
| 14ER15-1-02 | **479** | 0.054225 | 0.002110 | 0.282520 | 0.000019 | -8.9 | **1.0** | 0.7 | 1067 | **1391** | -0.94 |
| 14ER15-1-03 | **479** | 0.035977 | 0.001440 | 0.282511 | 0.000019 | -9.2 | **0.9** | 0.7 | 1060 | **1397** | -0.96 |
| 14ER15-1-04 | **479** | 0.036268 | 0.001498 | 0.282533 | 0.000015 | -8.5 | **1.6** | 0.5 | 1031 | **1349** | -0.95 |
| 14ER15-1-05 | **479** | 0.081505 | 0.003188 | 0.282579 | 0.000019 | -6.8 | **2.7** | 0.7 | 1011 | **1279** | -0.90 |
| 14ER15-1-06 | **479** | 0.027774 | 0.001111 | 0.282524 | 0.000016 | -8.8 | **1.4** | 0.6 | 1033 | **1362** | -0.97 |
| 14ER15-1-07 | **479** | 0.046368 | 0.001790 | 0.282539 | 0.000016 | -8.3 | **1.7** | 0.6 | 1031 | **1343** | -0.95 |
| 14ER15-1-08 | **479** | 0.089705 | 0.003383 | 0.282581 | 0.000023 | -6.7 | **2.7** | 0.8 | 1013 | **1278** | -0.90 |
| 14ER15-1-09 | **479** | 0.047073 | 0.001868 | 0.282571 | 0.000021 | -7.1 | **2.9** | 0.7 | 986 | **1270** | -0.94 |
| 14ER15-1-10 | **479** | 0.055915 | 0.002287 | 0.282583 | 0.000029 | -6.7 | **3.1** | 1.0 | 980 | **1252** | -0.93 |
| 14ER7-5-01 | **479** | 0.026707 | 0.001080 | 0.282583 | 0.000018 | -6.7 | **3.5** | 0.6 | 949 | **1229** | -0.97 |
| 14ER7-5-02 | **479** | 0.018637 | 0.000786 | 0.282565 | 0.000018 | -7.3 | **3.0** | 0.6 | 966 | **1262** | -0.98 |
| 14ER7-5-03 | **479** | 0.021163 | 0.000875 | 0.282557 | 0.000017 | -7.6 | **2.7** | 0.6 | 980 | **1283** | -0.97 |
| 14ER7-5-04 | **479** | 0.026692 | 0.001114 | 0.282540 | 0.000020 | -8.2 | **2.0** | 0.7 | 1010 | **1326** | -0.97 |
| 14ER7-5-05 | **479** | 0.021851 | 0.000910 | 0.282550 | 0.000026 | -7.9 | **2.4** | 0.9 | 992 | **1300** | -0.97 |
| 14ER7-5-06 | **479** | 0.030426 | 0.001331 | 0.282557 | 0.000018 | -7.6 | **2.5** | 0.6 | 992 | **1291** | -0.96 |
| 14ER7-5-07 | **479** | 0.021583 | 0.000892 | 0.282560 | 0.000018 | -7.5 | **2.8** | 0.6 | 977 | **1277** | -0.97 |
| 14ER7-5-08 | **479** | 0.013247 | 0.000557 | 0.282561 | 0.000018 | -7.5 | **2.9** | 0.6 | 967 | **1268** | -0.98 |
| 14ER5-1-01 | **481** | 0.018252 | 0.000742 | 0.282561 | 0.000020 | -7.5 | **2.9** | 0.7 | 972 | **1271** | -0.98 |
| 14ER5-1-02 | **481** | 0.017140 | 0.000701 | 0.282531 | 0.000020 | -8.5 | **1.9** | 0.7 | 1011 | **1336** | -0.98 |
| 14ER5-1-03 | **481** | 0.019808 | 0.000814 | 0.282574 | 0.000023 | -7.0 | **3.3** | 0.8 | 955 | **1243** | -0.98 |
| 14ER5-1-04 | **481** | 0.020659 | 0.000854 | 0.282566 | 0.000023 | -7.3 | **3.0** | 0.8 | 968 | **1262** | -0.97 |
| 14ER5-1-05 | **481** | 0.016391 | 0.000664 | 0.282551 | 0.000017 | -7.8 | **2.6** | 0.6 | 983 | **1291** | -0.98 |
| 14ER5-1-06 | **481** | 0.015882 | 0.000650 | 0.282548 | 0.000018 | -7.9 | **2.5** | 0.6 | 987 | **1298** | -0.98 |
| 14ER5-1-07 | **481** | 0.015748 | 0.000646 | 0.282537 | 0.000021 | -8.3 | **2.1** | 0.7 | 1002 | **1321** | -0.98 |
| 14ER5-1-08 | **481** | 0.015007 | 0.000614 | 0.282556 | 0.000018 | -7.6 | **2.8** | 0.6 | 975 | **1279** | -0.98 |
| 14ER5-1-09 | **481** | 0.018726 | 0.000769 | 0.282545 | 0.000023 | -8.0 | **2.3** | 0.8 | 994 | **1306** | -0.98 |
| 14ER5-1-10 | **481** | 0.012936 | 0.000545 | 0.282518 | 0.000018 | -9.0 | **1.4** | 0.6 | 1026 | **1363** | -0.98 |
| 13ER30-1-01 | **482** | 0.036339 | 0.001356 | 0.282459 | 0.000013 | -11.1 | **-0.9** | 0.4 | 1132 | **1511** | -0.96 |
| 13ER30-1-02 | **482** | 0.020415 | 0.000854 | 0.282472 | 0.000015 | -10.6 | **-0.3** | 0.5 | 1099 | **1471** | -0.97 |
| 13ER30-1-04 | **482** | 0.021658 | 0.000897 | 0.282460 | 0.000015 | -11.0 | **-0.7** | 0.5 | 1117 | **1500** | -0.97 |
| 13ER30-1-05 | **482** | 0.022131 | 0.000883 | 0.282489 | 0.000014 | -10.0 | **0.3** | 0.5 | 1075 | **1433** | -0.97 |
| 13ER30-1-06 | **482** | 0.025619 | 0.001057 | 0.282446 | 0.000012 | -11.5 | **-1.2** | 0.4 | 1141 | **1533** | -0.97 |
| 13ER30-1-03 | **501** | 0.022134 | 0.000931 | 0.282487 | 0.000020 | -10.1 | **0.6** | 0.7 | 1080 | **1428** | -0.97 |
| 13ER30-1-07 | **501** | 0.022296 | 0.000880 | 0.282473 | 0.000012 | -10.6 | **0.2** | 0.4 | 1097 | **1457** | -0.97 |
| 13ER30-1-08 | **501** | 0.043985 | 0.001635 | 0.282484 | 0.000017 | -10.2 | **0.3** | 0.6 | 1105 | **1449** | -0.95 |
| ER13-1-01 | **737** | 0.045573 | 0.001653 | 0.282332 | 0.000044 | -15.6 | **-0.1** | 1.6 | 1322 | **1655** | -0.95 |
| ER13-1-02 | **737** | 0.044237 | 0.001515 | 0.282409 | 0.000014 | -12.8 | **2.7** | 0.5 | 1208 | **1479** | -0.95 |
| ER13-1-03 | **737** | 0.050989 | 0.001802 | 0.282423 | 0.000013 | -12.3 | **3.0** | 0.5 | 1197 | **1456** | -0.95 |
| ER13-1-04 | **737** | 0.033161 | 0.001246 | 0.282236 | 0.000016 | -19.0 | **-3.3** | 0.6 | 1442 | **1857** | -0.96 |
| ER13-1-05 | **737** | 0.044871 | 0.001608 | 0.282428 | 0.000015 | -12.2 | **3.3** | 0.5 | 1183 | **1439** | -0.95 |
| ER13-1-06 | **737** | 0.098516 | 0.003146 | 0.282536 | 0.000028 | -8.3 | **6.4** | 1.0 | 1074 | **1245** | -0.91 |
| ER13-1-07 | **737** | 0.051383 | 0.001783 | 0.282430 | 0.000014 | -12.1 | **3.3** | 0.5 | 1186 | **1440** | -0.95 |
| ER13-1-08 | **737** | 0.055495 | 0.001888 | 0.282429 | 0.000012 | -12.1 | **3.2** | 0.4 | 1191 | **1445** | -0.94 |
| ER13-1-09 | **737** | 0.045492 | 0.001612 | 0.282436 | 0.000015 | -11.9 | **3.6** | 0.5 | 1172 | **1421** | -0.95 |
| 12ER7-1-01 | **737** | 0.085806 | 0.002153 | 0.282545 | 0.000037 | -8.0 | **7.2** | 1.3 | 1032 | **1194** | -0.94 |
| 12ER7-1-02 | **737** | 0.105054 | 0.002653 | 0.282601 | 0.000034 | -6.0 | **8.9** | 1.2 | 964 | **1083** | -0.92 |
| 12ER7-1-03 | **737** | 0.075178 | 0.001958 | 0.282670 | 0.000037 | -3.6 | **11.7** | 1.3 | 846 | **906** | -0.94 |
| 12ER7-1-04 | **737** | 0.103477 | 0.002842 | 0.282641 | 0.000037 | -4.6 | **10.3** | 1.3 | 910 | **999** | -0.91 |
| 11ER24-1-01 | **851** | 0.027886 | 0.001073 | 0.282482 | 0.000013 | -10.3 | **8.0** | 0.5 | 1091 | **1234** | -0.97 |
| 11ER24-1-02 | **791** | 0.036060 | 0.001351 | 0.282462 | 0.000016 | -11.0 | **5.8** | 0.6 | 1127 | **1323** | -0.96 |
| 11ER24-1-03 | **762** | 0.047363 | 0.001781 | 0.282454 | 0.000027 | -11.2 | **4.7** | 1.0 | 1152 | **1372** | -0.95 |
| 11ER24-1-04 | **791** | 0.034024 | 0.001210 | 0.282506 | 0.000015 | -9.4 | **7.4** | 0.5 | 1061 | **1220** | -0.96 |
| 11ER24-1-05 | **791** | 0.045673 | 0.001557 | 0.282508 | 0.000014 | -9.3 | **7.3** | 0.5 | 1068 | **1227** | -0.95 |
| 11ER24-1-06 | **791** | 0.030466 | 0.001115 | 0.282463 | 0.000010 | -10.9 | **6.0** | 0.4 | 1119 | **1313** | -0.97 |
| 11ER24-1-07 | **851** | 0.018517 | 0.000742 | 0.282472 | 0.000010 | -10.6 | **7.8** | 0.4 | 1095 | **1244** | -0.98 |
| 11ER24-1-08 | **851** | 0.041529 | 0.001462 | 0.282491 | 0.000013 | -9.9 | **8.1** | 0.5 | 1089 | **1227** | -0.96 |
| 11ER24-1-09 | **762** | 0.013783 | 0.000604 | 0.282441 | 0.000038 | -11.7 | **4.8** | 1.3 | 1134 | **1363** | -0.98 |
| 11ER24-1-10 | **791** | 0.033125 | 0.001280 | 0.282509 | 0.000018 | -9.3 | **7.5** | 0.6 | 1059 | **1216** | -0.96 |
| 14ER17-1-01 | **791** | 0.044015 | 0.001768 | 0.282517 | 0.000019 | -9.0 | **7.5** | 0.7 | 1061 | **1214** | -0.95 |
| 14ER17-1-02 | **791** | 0.058751 | 0.002112 | 0.282533 | 0.000023 | -8.5 | **7.9** | 0.8 | 1048 | **1190** | -0.94 |
| 14ER17-1-03 | **791** | 0.034320 | 0.001417 | 0.282509 | 0.000019 | -9.3 | **7.4** | 0.7 | 1063 | **1220** | -0.96 |
| 14ER17-1-04 | **791** | 0.033895 | 0.001396 | 0.282539 | 0.000019 | -8.2 | **8.5** | 0.7 | 1019 | **1152** | -0.96 |
| ER24-1-01 | **792** | 0.050805 | 0.001942 | 0.282434 | 0.000029 | -12.0 | **4.5** | 1.0 | 1186 | **1405** | -0.94 |
| ER24-1-02 | **792** | 0.028524 | 0.001072 | 0.282427 | 0.000027 | -12.2 | **4.7** | 1.0 | 1168 | **1392** | -0.97 |
| ER24-1-03 | **792** | 0.026961 | 0.001011 | 0.282404 | 0.000023 | -13.0 | **4.0** | 0.8 | 1198 | **1441** | -0.97 |
| ER24-1-04 | **837** | 0.051523 | 0.001883 | 0.282485 | 0.000025 | -10.1 | **7.3** | 0.9 | 1110 | **1264** | -0.94 |
| ER24-1-05 | **837** | 0.037318 | 0.001406 | 0.282375 | 0.000019 | -14.0 | **3.7** | 0.7 | 1252 | **1493** | -0.96 |
| ER24-1-06 | **837** | 0.046008 | 0.001649 | 0.282441 | 0.000020 | -11.7 | **5.9** | 0.7 | 1166 | **1354** | -0.95 |
| ER24-1-07 | **792** | 0.052259 | 0.001914 | 0.282409 | 0.000021 | -12.8 | **3.7** | 0.7 | 1221 | **1460** | -0.94 |
| ER24-1-08 | **792** | 0.043275 | 0.001643 | 0.282417 | 0.000023 | -12.6 | **4.1** | 0.8 | 1200 | **1433** | -0.95 |
| ER24-1-09 | **837** | 0.049895 | 0.001849 | 0.282418 | 0.000021 | -12.5 | **5.0** | 0.7 | 1206 | **1413** | -0.94 |
| ER24-1-10 | **792** | 0.055136 | 0.001963 | 0.282436 | 0.000025 | -11.9 | **4.6** | 0.9 | 1183 | **1401** | -0.94 |
| ER24-1-11 | **792** | 0.043761 | 0.001637 | 0.282412 | 0.000024 | -12.7 | **3.9** | 0.9 | 1207 | **1444** | -0.95 |
| ER24-1-12 | **792** | 0.041210 | 0.001545 | 0.282406 | 0.000021 | -12.9 | **3.7** | 0.7 | 1213 | **1455** | -0.95 |
| 11ER23-1-01 | **850** | 0.032594 | 0.001059 | 0.282469 | 0.000010 | -10.7 | **7.5** | 0.4 | 1109 | **1263** | -0.97 |
| 11ER23-1-02 | **792** | 0.049925 | 0.001673 | 0.282456 | 0.000013 | -11.2 | **5.4** | 0.5 | 1146 | **1347** | -0.95 |
| 11ER23-1-03 | **850** | 0.041182 | 0.001389 | 0.282372 | 0.000009 | -14.1 | **3.9** | 0.3 | 1256 | **1492** | -0.96 |
| 11ER23-1-04 | **792** | 0.040345 | 0.001333 | 0.282432 | 0.000010 | -12.0 | **4.8** | 0.4 | 1169 | **1389** | -0.96 |
| 11ER23-1-05 | **850** | 0.032256 | 0.001078 | 0.282436 | 0.000014 | -11.9 | **6.3** | 0.5 | 1156 | **1337** | -0.97 |
| 11ER23-1-06 | **850** | 0.054536 | 0.001712 | 0.282459 | 0.000012 | -11.1 | **6.8** | 0.4 | 1143 | **1309** | -0.95 |
| 11ER23-1-07 | **850** | 0.041281 | 0.001418 | 0.282453 | 0.000025 | -11.3 | **6.7** | 0.9 | 1142 | **1312** | -0.96 |
| 11ER23-1-08 | **850** | 0.099911 | 0.003394 | 0.282522 | 0.000018 | -8.8 | **8.0** | 0.6 | 1102 | **1228** | -0.90 |
| 11ER23-1-09 | **792** | 0.039064 | 0.001338 | 0.282401 | 0.000024 | -13.1 | **3.7** | 0.9 | 1213 | **1459** | -0.96 |
| 11ER23-1-10 | **792** | 0.043650 | 0.001437 | 0.282475 | 0.000013 | -10.5 | **6.2** | 0.5 | 1111 | **1297** | -0.96 |
| 14ER13-1-01 | **793** | 0.046618 | 0.001847 | 0.282360 | 0.000022 | -14.6 | **2.0** | 0.8 | 1289 | **1567** | -0.94 |
| 14ER13-1-02 | **793** | 0.044892 | 0.001788 | 0.282362 | 0.000020 | -14.5 | **2.1** | 0.7 | 1284 | **1561** | -0.95 |
| 14ER13-1-03 | **793** | 0.040103 | 0.001613 | 0.282360 | 0.000020 | -14.6 | **2.1** | 0.7 | 1280 | **1559** | -0.95 |
| 14ER13-1-04 | **793** | 0.027661 | 0.001137 | 0.282340 | 0.000020 | -15.3 | **1.6** | 0.7 | 1292 | **1588** | -0.97 |
| 13ER41-1-01 | **794** | 0.037100 | 0.001342 | 0.282370 | 0.000027 | -14.2 | **2.6** | 1.0 | 1257 | **1527** | -0.96 |
| 13ER41-1-02 | **794** | 0.028488 | 0.001038 | 0.282263 | 0.000024 | -18.0 | **-1.0** | 0.9 | 1397 | **1756** | -0.97 |
| 13ER41-1-03 | **794** | 0.038222 | 0.001489 | 0.282379 | 0.000026 | -13.9 | **2.9** | 0.9 | 1249 | **1512** | -0.96 |
| 13ER41-1-04 | **794** | 0.031888 | 0.001161 | 0.282329 | 0.000021 | -15.7 | **1.3** | 0.7 | 1309 | **1613** | -0.97 |
| 13ER41-1-05 | **794** | 0.031103 | 0.001167 | 0.282302 | 0.000024 | -16.6 | **0.3** | 0.9 | 1347 | **1673** | -0.96 |
| 13ER41-1-06 | **794** | 0.036852 | 0.001413 | 0.282307 | 0.000025 | -16.4 | **0.3** | 0.9 | 1348 | **1670** | -0.96 |
| 13ER43-1-01 | **794** | 0.036470 | 0.001326 | 0.282332 | 0.000022 | -15.6 | **1.3** | 0.8 | 1311 | **1612** | -0.96 |
| 13ER43-1-02 | **794** | 0.017360 | 0.000659 | 0.282342 | 0.000023 | -15.2 | **2.0** | 0.8 | 1274 | **1568** | -0.98 |
| 13ER43-1-03 | **794** | 0.012876 | 0.000527 | 0.282299 | 0.000022 | -16.7 | **0.5** | 0.8 | 1328 | **1659** | -0.98 |
| 13ER43-1-04 | **794** | 0.021992 | 0.000833 | 0.282325 | 0.000023 | -15.8 | **1.3** | 0.8 | 1303 | **1611** | -0.97 |
| 13ER43-1-05 | **794** | 0.027936 | 0.001053 | 0.282286 | 0.000024 | -17.2 | **-0.2** | 0.8 | 1365 | **1706** | -0.97 |
| 13ER43-1-06 | **794** | 0.023833 | 0.000906 | 0.282285 | 0.000024 | -17.2 | **-0.2** | 0.9 | 1361 | **1703** | -0.97 |
| 13ER44-1-01 | **794** | 0.029196 | 0.001134 | 0.282286 | 0.000023 | -17.2 | **-0.2** | 0.8 | 1368 | **1708** | -0.97 |
| 13ER44-1-02 | **794** | 0.015668 | 0.000605 | 0.282298 | 0.000025 | -16.8 | **0.4** | 0.9 | 1333 | **1665** | -0.98 |
| 13ER44-1-03 | **794** | 0.016969 | 0.000647 | 0.282314 | 0.000025 | -16.2 | **1.0** | 0.9 | 1312 | **1629** | -0.98 |
| 13ER44-1-04 | **794** | 0.020126 | 0.000769 | 0.282316 | 0.000022 | -16.1 | **1.0** | 0.8 | 1313 | **1628** | -0.98 |
| 13ER44-1-05 | **794** | 0.018255 | 0.000684 | 0.282309 | 0.000026 | -16.4 | **0.8** | 0.9 | 1319 | **1641** | -0.98 |
| 13ER44-1-06 | **794** | 0.020967 | 0.000786 | 0.282345 | 0.000021 | -15.1 | **2.0** | 0.7 | 1273 | **1564** | -0.98 |
| 14ER11-1-01 | **795** | 0.019903 | 0.000827 | 0.282443 | 0.000015 | -11.6 | **5.5** | 0.5 | 1138 | **1346** | -0.98 |
| 14ER11-1-02 | **795** | 0.021030 | 0.000854 | 0.282462 | 0.000018 | -10.9 | **6.2** | 0.6 | 1112 | **1303** | -0.97 |
| 14ER11-1-03 | **795** | 0.019422 | 0.000798 | 0.282437 | 0.000018 | -11.8 | **5.3** | 0.6 | 1145 | **1358** | -0.98 |
| 14ER11-1-04 | **795** | 0.022558 | 0.000933 | 0.282454 | 0.000018 | -11.2 | **5.8** | 0.6 | 1126 | **1324** | -0.97 |
| 14ER11-1-05 | **795** | 0.024507 | 0.001016 | 0.282422 | 0.000018 | -12.4 | **4.7** | 0.6 | 1173 | **1398** | -0.97 |
| 14ER11-1-06 | **795** | 0.021396 | 0.000939 | 0.282450 | 0.000019 | -11.4 | **5.7** | 0.7 | 1132 | **1334** | -0.97 |
| 14ER11-1-07 | **795** | 0.028497 | 0.001158 | 0.282458 | 0.000019 | -11.1 | **5.8** | 0.7 | 1127 | **1324** | -0.97 |
| 14ER11-1-08 | **795** | 0.023574 | 0.000954 | 0.282434 | 0.000016 | -11.9 | **5.1** | 0.6 | 1154 | **1369** | -0.97 |
| 14ER11-1-09 | **795** | 0.088909 | 0.003514 | 0.282490 | 0.000023 | -10.0 | **5.7** | 0.8 | 1154 | **1330** | -0.89 |
| 14ER11-1-10 | **795** | 0.016945 | 0.000747 | 0.282438 | 0.000016 | -11.8 | **5.4** | 0.6 | 1142 | **1354** | -0.98 |
| 13ER12-1-01 | **846** | 0.051687 | 0.001888 | 0.282154 | 0.000032 | -21.9 | **-4.2** | 1.1 | 1584 | **1998** | -0.94 |
| 13ER12-1-02 | **846** | 0.055839 | 0.002110 | 0.282202 | 0.000027 | -20.2 | **-2.7** | 0.9 | 1525 | **1899** | -0.94 |
| 13ER12-1-03 | **846** | 0.058545 | 0.002156 | 0.282165 | 0.000025 | -21.5 | **-4.0** | 0.9 | 1581 | **1984** | -0.94 |
| 13ER13-1-01 | **846** | 0.050153 | 0.001846 | 0.282115 | 0.000022 | -23.2 | **-5.6** | 0.8 | 1638 | **2083** | -0.94 |
| 13ER13-1-02 | **846** | 0.060382 | 0.002194 | 0.282113 | 0.000022 | -23.3 | **-5.9** | 0.8 | 1657 | **2101** | -0.93 |
| 13ER13-1-03 | **846** | 0.054364 | 0.001881 | 0.282166 | 0.000019 | -21.4 | **-3.8** | 0.7 | 1566 | **1970** | -0.94 |
| 13ER13-1-04 | **846** | 0.069760 | 0.002347 | 0.282124 | 0.000021 | -22.9 | **-5.5** | 0.8 | 1647 | **2080** | -0.93 |
| 13ER13-1-05 | **846** | 0.072657 | 0.002734 | 0.282263 | 0.000029 | -18.0 | **-0.9** | 1.0 | 1463 | **1786** | -0.92 |
| 13ER13-1-06 | **846** | 0.056827 | 0.002037 | 0.282111 | 0.000020 | -23.4 | **-5.8** | 0.7 | 1652 | **2098** | -0.94 |
| 13ER13-1-07 | **846** | 0.043573 | 0.001519 | 0.282129 | 0.000023 | -22.7 | **-4.9** | 0.8 | 1604 | **2041** | -0.95 |
| 12ER28-1-01 | **850** | 0.063650 | 0.002539 | 0.282149 | 0.000020 | -22.0 | **-4.7** | 0.7 | 1620 | **2030** | -0.92 |
| 12ER28-1-02 | **850** | 0.046993 | 0.001915 | 0.282125 | 0.000018 | -22.9 | **-5.2** | 0.6 | 1627 | **2061** | -0.94 |
| 12ER28-1-03 | **850** | 0.062718 | 0.002507 | 0.282142 | 0.000018 | -22.3 | **-4.9** | 0.6 | 1628 | **2044** | -0.92 |
| 12ER28-1-04 | **850** | 0.060510 | 0.002364 | 0.282126 | 0.000018 | -22.8 | **-5.4** | 0.6 | 1645 | **2074** | -0.93 |
| 12ER28-1-05 | **850** | 0.055588 | 0.002230 | 0.282136 | 0.000018 | -22.5 | **-5.0** | 0.6 | 1625 | **2048** | -0.93 |
| 12ER28-1-06 | **850** | 0.047412 | 0.001924 | 0.282125 | 0.000016 | -22.9 | **-5.2** | 0.6 | 1627 | **2061** | -0.94 |
| ER7-1-01 | **851** | 0.031973 | 0.001131 | 0.282361 | 0.000015 | -14.5 | **3.6** | 0.5 | 1263 | **1507** | -0.97 |
| ER7-1-02 | **851** | 0.043259 | 0.001543 | 0.282423 | 0.000018 | -12.3 | **5.6** | 0.6 | 1189 | **1383** | -0.95 |
| ER7-1-03 | **851** | 0.040219 | 0.001405 | 0.282410 | 0.000021 | -12.8 | **5.2** | 0.7 | 1203 | **1407** | -0.96 |
| ER7-1-04 | **851** | 0.035432 | 0.001216 | 0.282416 | 0.000016 | -12.6 | **5.5** | 0.6 | 1188 | **1387** | -0.96 |
| ER7-1-05 | **851** | 0.017383 | 0.000645 | 0.282327 | 0.000018 | -15.7 | **2.7** | 0.6 | 1294 | **1565** | -0.98 |
| ER7-1-06 | **851** | 0.036064 | 0.001275 | 0.282393 | 0.000014 | -13.4 | **4.7** | 0.5 | 1222 | **1440** | -0.96 |
| 14ER3-6-01 | **1785** | 0.020079 | 0.000807 | 0.281534 | 0.000016 | -43.8 | **-5.0** | 0.6 | 2391 | **2758** | -0.98 |
| 14ER3-6-02 | **1785** | 0.008465 | 0.000377 | 0.281478 | 0.000014 | -45.7 | **-6.4** | 0.5 | 2439 | **2848** | -0.99 |
| 14ER3-6-03 | **1785** | 0.013796 | 0.000536 | 0.281530 | 0.000020 | -43.9 | **-4.8** | 0.7 | 2379 | **2746** | -0.98 |
| 14ER3-6-04 | **1785** | 0.015858 | 0.000698 | 0.281519 | 0.000021 | -44.3 | **-5.4** | 0.7 | 2404 | **2782** | -0.98 |
| 14ER3-6-05 | **1785** | 0.012455 | 0.000504 | 0.281517 | 0.000018 | -44.4 | **-5.2** | 0.6 | 2395 | **2773** | -0.98 |
| 14ER3-6-06 | **1785** | 0.018192 | 0.000797 | 0.281515 | 0.000017 | -44.5 | **-5.6** | 0.6 | 2416 | **2799** | -0.98 |
| 14ER3-1-01 | **1860** | 0.015174 | 0.000693 | 0.281497 | 0.000022 | -45.1 | **-4.5** | 0.8 | 2434 | **2784** | -0.98 |
| 14ER3-1-02 | **1860** | 0.020481 | 0.000909 | 0.281527 | 0.000020 | -44.0 | **-3.7** | 0.7 | 2407 | **2737** | -0.97 |
| 14ER3-1-03 | **1860** | 0.012194 | 0.000529 | 0.281504 | 0.000022 | -44.8 | **-4.0** | 0.8 | 2414 | **2757** | -0.98 |
| 14ER3-1-04 | **1860** | 0.008976 | 0.000408 | 0.281267 | 0.000015 | -53.2 | **-12.3** | 0.5 | 2725 | **3264** | -0.99 |
| 14ER3-1-05 | **1860** | 0.010195 | 0.000435 | 0.281600 | 0.000022 | -41.4 | **-0.5** | 0.8 | 2278 | **2538** | -0.99 |
| 14ER3-1-06 | **1860** | 0.008552 | 0.000393 | 0.281484 | 0.000016 | -45.5 | **-4.6** | 0.6 | 2433 | **2790** | -0.99 |
| 14ER3-1-07 | **1860** | 0.010420 | 0.000484 | 0.281467 | 0.000017 | -46.1 | **-5.3** | 0.6 | 2461 | **2833** | -0.99 |
| 14ER3-1-08 | **1860** | 0.015590 | 0.000730 | 0.281474 | 0.000016 | -45.9 | **-5.4** | 0.6 | 2468 | **2839** | -0.98 |
| 14ER3-1-09 | **1860** | 0.007420 | 0.000349 | 0.281473 | 0.000016 | -45.9 | **-4.9** | 0.6 | 2444 | **2810** | -0.99 |
| 14ER3-1-10 | **1860** | 0.006218 | 0.000291 | 0.281467 | 0.000016 | -46.2 | **-5.1** | 0.6 | 2450 | **2820** | -0.99 |

**Supplementary Table S4**. The locations, ages, Hf/Nd isotopes and reference citations for the mafic igneous rocks in the Erguna Massif

| **Order** | **Sample** | **Latitude (N)** | **Longitude (E)** | **Location** | **Lithology** | **Age (Ma)** | **ε_Hf/Nd_(t)** | **T_DM1_ ages (Ma)** | **References** |
| --- | --- | --- | --- | --- | --- | --- | --- | --- | --- |
| 1 | 12ER21 | 50°09’40” | 120°11’58” | Southern Erguna | Basaltic andesite | **119** | 0.27–0.52 (Nd) | 741–795 | Unpublished |
| 2 | ER3 | 50°19’57” | 120°15’00” | Sanhe town | Basaltic andesite | **125** | 0.69–0.85 (Nd) | 806–818 | Unpublished |
| 3 | MZ21 | 49°26’42” | 117°02’31” | Chagantaolegai mountain | Basalt | **129** | 2.21–2.39 (Nd) | 689–691 | Unpublished |
| 4 | 13ER38 | 51°43’35” | 120°43’49” | Northern Moerdaoga | Basaltic andesite | **158** | 3.29–3.49 (Nd) | 1140–1237 | Unpublished |
| 5 | 13ER52 | 49°32’33” | 119°51’11” | Taohai Ranch | Basaltic andesite | **180** | 4.13–4.25 (Nd) | 686–695 | Ref.^47^ |
| 6 | ER18 | 50°44’02” | 120°11’57” | Shanghulin basin | Trachybasalt | **183** | 0.44–3.56 (Hf) | 714–814 | Ref.^47^ |
| 7 | 13ER20 | 51°23’27” | 121°03’07” | Northern Moerdaoga | Basaltic andesite | **193** | -1.45–2.66 (Hf) | 741–901 | Ref.^47^ |
| 8 | 12ER30 | 52°25’09” | 122°34’00” | Northern Mangui | Gabbro-diorite | **200** | 2.90–5.89 (Hf) | 617–736 | Ref.^41^ |
| 9 | 13ER8 | 51°40’17” | 121°32’07” | Western Alongshan | Gabbro | **204** | -1.92–1.31 (Hf) | 826–955 | Ref.^41^ |
| 10 | 13ER34 | 52°02’37” | 120°51’48” | Northern Moerdaoga | Gabbro | **205** | -2.86–1.88 (Hf) | 779–963 | Ref.^41^ |
| 11 | 1665-1 | – | – | Northern Qiqian | Gabbro-diorite | **213** | -0.38–2.72 (Hf) | 751–873 | Ref.^48^ |
| 12 | 13ER13-6 | 52°03’26” | 122°05’33” | Northeastern Mangui | Gabbro | **227** | 2.18–8.81 (Hf) | 541–850 | Unpublished |
| 13 | 15XL01 | 51°46’21” | 125°44’49” | Northeastern Xinglong | Gabbro | **447** | 6.42–13.33 (Hf) | 531–841 | Ref.^49^ |
| 14 | 14ER6 | 52°21’43” | 124°47’44” | Tahe | Gabbro | **463** | 2.70–4.49 (Hf) | 890–980 | Unpublished |
| 15 | ER25/11ER6 | 51°00’23” | 120°03’00” | Shanghulin–Enhe highway | Gabbro | **465** | -0.27–3.89 (Hf) | 928–1091 | Ref.^44^ |
| 16 | 1649-1 | 52°49’30” | 120°33’43” | Liangjianfang | Gabbro | **466** | 2.99–4.98 (Hf) | 877–956 | Ref.^50^ |
| 17 | 14ER12 | 52°29’44” | 123°44’15” | Southern Pangu | Gabbro | **483** | 1.86–4.19 (Hf) | 927–1012 | Unpublished |
| 18 | ER27/11ER19 | 51°18’09” | 119°53’03” | Shiwei–Jiuka road | Gabbro | **792** | 2.48–5.41 (Hf) | 1161–1267 | Ref.^36^ |

**References**

1. Tang, J. et al. Geochronology and geochemistry of Neoproterozoic magmatism in the Erguna Massif, NE China: Petrogenesis and implications for the breakup of the Rodinia supercontinent. *Precambrian Res*. **224**, 597–611, https://doi.org/10.1016/j.precamres.2012.10.019 (2013).
2. Tang, J., Xu, W. L., Wang, F., Zhao, S. & Wang, W. Early Mesozoic southwards subduction history of the Mongol-Okhotsk oceanic plate: Evidence from geochronology and geochemistry of Early Mesozoic intrusive rocks in the Erguna massif, NE China. *Gondwana Res*. **31**, 218–240, https://doi.org/10.1016/j.gr.2014.12.010 (2016).
3. Zhao, S., Xu, W. L., Wang, W., Tang, J. & Zhang, Y. H. Geochronology and geochemistry of Middle-Late Ordovician granites and gabbros in the Erguna region, NE China: Implications for the tectonic evolution of the Erguna Massif. *J. Earth Sci-China*. **25**(5), 841–853, https://doi.org/10.1007/s12583-014-0476-9 (2014).
4. Wang, W., Tang, J., Xu, W. L. & Wang, F. Geochronology and geochemistry of Early Jurassic volcanic rocks in the Erguna Massif, northeast China: Petrogenesis and implications for the tectonic evolution of the Mongol–Okhotsk suture belt. *Lithos*. **218****–219**, 73–86, https://doi.org/10.1016/j.lithos.2015.01.012 (2015).
5. Gou, J., Sun, D. Y., Ren, Y. S., Hou, X. G. & Yang, D. G. Geochemical and Hf isotopic compositions of Late Triassic-Early Jurassic intrusions of the Erguna Block, Northeast China: Petrogenesis and tectonic implications. *Int. Geol. Rev*. **59**(3), 347–367, https://doi.org/10.1080/00206814.2016.1266448 (2017).
6. Feng, Z. Q. et al. Silurian magmatism on the eastern margin of the Erguna Block, NE China: Evolution of the northern Great Xing'an Range. *Gondwana Res*. **61**, 46–62, https://doi.org/10.1016/j.gr.2018.04.011 (2018).
7. Gou, J., Sun, D. Y., Mao, A. Q., Yang, D. G. & Tang, Z. Y. Geochronology and geochemistry of Ordovician plutons in the Erguna Block (NE China): further insights into the tectonic evolution of the Xing'an-Mongolia Orogenic Belt. *Int. Geol. Rev.* **61**(8), 936–955, https://doi.org/10.1080/00206814.2018.1479890 (2019).
